# Supplementary material for: Acceptance of and Adherence to a Four-Dose RTS,S/AS01 Schedule: Findings from a Longitudinal Qualitative Evaluation Study for the Malaria Vaccine Implementation Programme
Source: Vaccines (Basel). 2023 Dec 1;11(12):1801. doi: 10.3390/vaccines11121801 (PMC10747521; doi:10.3390/vaccines11121801)
Supplement: Supplementary file 1 [file vaccines-11-01801-s001.zip › Suppl 2_HUS Study Protocol_21Sep2018.pdf]

The Dynamics of Healthcare Utilization in the Context of RTS,S/AS01 Vaccine Introduction in Ghana, Kenya, and Malawi

A qualitative longitudinal study

Study Protocol
version: September 21, 2018

Scott Gordon,[1] Project Director

Principal Investigators

Jessica Price,[1] HUS Study Lead
Margaret Gyapong,[2] Ghana Team Lead
Jenny Hill,[3] Kenya Team Lead
Nicola Desmond,[4] Malawi Team Lead

Study Teams

HUS Global
Orianne Berraud[1]
Juliette Arnaud[1]

Ghana Team
Evelyn Korkor Anash
Seth Owusu Agyei
Kofi Awusabo-Asare
Ellis Owusu-Dabo
Phedelia Doegah
Arti Singh
Samuel Fosu Gyasi
Alison Krentel

Kenya Team
George Okello, co-PI[6]
Simon Kariuki, co-PI[6]
Feiko ter Kuile[3]
Jayne Webster[7]
Meghna Desai[5]
Nelli Westercamp[5]
Brent Wolff[5]
Mohamed Jalloh[5]
Caroline Jones[8]

Malawi Team
Kate Gooding
Linda Nyondo-Mipando
Anja Terlouw
Neil French

[1] PATH, Seattle and Geneva
[2] University of Health and Allied Sciences, Ghana
[3] Liverpool School of Tropical Medicine
[4] Malawi-Liverpool-Welcome Trust Clinical Research Programme
[5] US Centers for Disease Control and Prevention (CDC), Center for Global Health
[6] Kenya Medical Research Institute, Centre for Global Health Research (KEMRI), Kenya
[7] Disease Control Department, London School of Hygiene & Tropical Medicine (LSHTM)
[8] Kemri Welcome Trust Research Programme (KWRTP), Kenya

ABBREVIATIONS

ACT	Artemisinin-based combination therapy
BCG	Bacille Calmette-Guérin (Tuberculosis) Vaccine
DHTM	District Health Management Team
DPT-HepB-Hib (penta)	Diphtheria and tetanus toxoids and whole-cell pertussis vaccine – Hepatitis B vaccine - *Haemophilus influenzae* type b
EPI	Expanded Programme on Immunization
FGD	Focus Group Discussion
HH	Household
HSA	Health Surveillance Assistant
HUS	The Dynamics of Healthcare Utilization in the Context of RTS,S/AS01 Vaccine Introduction
IDI	Individual interviews (including in-depth and semi-structured)
LLIN	Long-lasting insecticide-treated net
IMCI	Integrated management of childhood illnesses
ITN	Insecticide-treated nets
IPTp	Intermittent Preventive Treatment in pregnancy
IPV	Inactivated Poliovirus Vaccine
LSHTM	London School of Hygiene and Tropical Medicine
LSTM	Liverpool School of Tropical Medicine
MCH	Maternal and child health services
MLW	Malawi Liverpool-Wellcome Trust Clinical Research Programme
MenA	Meningititis A Vaccine
(M)R	Measles Rubella Vaccine
MVIP	Malaria Vaccine Implementation Programme
OPV	Oral Polio Vaccine
PCG	Primary Child Caregiver
PCV	Pneumococcal Conjugate Vaccine
PfPr	*Plasmodium falicparum* parasite prevalence
QPR	Qualitative Panel Research
Rota	Rotavirus Vaccine
UHAS	University of Health and Allied Sciences
VitA	Vitamin A supplementation
WHO	World Health Organization
YF	Yellow Fever Vaccine

Table of Contents

1. Background & Rationale 1
   1.1. RTS,S/AS01: Phase 3 Outcomes 1
      1.1.1. Efficacy 1
      1.1.2. Safety 1
      1.1.3. Impact 2
   1.2. Malaria Vaccine Implementation Programme (MVIP) 2
      1.2.1. MVIP Objectives & the Healthcare Utilization Study (HUS) 2
      1.2.2. MVIP Countries 3
      1.2.3. HUS Research Partners 4
2. HUS Purpose & Conceptual Foundations 4
   2.1. Overall Purpose & Research Questions 4
   2.2. RTS,S Uptake Logic Model 6
   2.3. Critical Behavioral Outcomes 6
   2.4. Ecological Framework 8
   2.5. Qualitative Panel Research 8
3. Methods 9
   3.1. Sampling 10
      3.1.1. Community Sites 11
      3.1.2. Study Groups: Overview 12
      3.1.3. Primary Child Caregivers (PCGs) 12
      3.1.4. Health Leaders, Program Mangers, & Providers 16
      3.1.5. Community Leaders, Opinion Shapers, & Others 17
      3.1.6. Iteratively-derived Samples 18
   3.2. Data Collection Procedures 19
      3.2.1. Ethnographic Immersion 19
      3.2.2. PCG Interviews 19
      3.2.3. Health Leaders, Managers, & Providers Interviews & FGDs 20
      3.2.4. Community Members Interviews & FGDs 21
4. Data Management 22
   4.1. Record Keeping 22
   4.2. Transcription & Translation 23
   4.3. Categorized & Coded Data 23
   4.4. Quantitative Data 23
   4.5. Backup & Archiving 23
   4.6. Confidentiality & Ethics 24
5. Data Analysis 24
   5.1. Categorizing through Thematic Analysis 24
   5.2. Connecting Themes Within Cases, Across Time, & Across Datasets 25

5.3. Causal Explanation 26
5.4. Coding Consistency Within & Across Research Sites 26
5.5. Exemplary Quotes 26
5.6. Validating Interpretations & Comparing (Sub)-samples 26
5.7. Country- & Global-Level Analysis Roles 27

6. Study Timing & Management 27

6.1. Fieldwork Timing 27
6.2. Coordination & Roles 27
6.3. Staff Training 30
6.4. Crisis Communication 30

7. Ethical Procedures 31

7.1. Informed consent 32
7.2. Risks 34
7.3. Benefits 34
7.4. Confidentiality 34
7.5. Compensation 34

8. Dissemination & Future Data Use 35

8.1. Dissemination 35
8.2. Future Data Use 35

Annex 1: HUS Research Partners 37
Annex 2: Ghana Background 38
Annex 3: Kenya Background 44
Annex 4: Malawi Background 51
Annex 5: Participant Observation Guide 56
Annex 6: Primary Caregiver Profile Sheet 59
Annex 7: Primary Caregiver Interview Guides 61
Annex 8: Health Provider Profile Sheet and FGD Guide 75
Annex 9: Community Leader/Member R1 Profile Sheet & Interview/FGD Guides 79
Annex 10: PCG Interview Tracking Log 81
Annex 11: Health Leader & Manager Interview Tracking Log 82
Annex 12: Health Provider Focus Group Tracking Log 83
Annex 13: Community Leader Tracking Log 84
Annex 14: Male Household Head Focus Group Tracking Log 85
Annex 15: Female Elder Focus Group and Individual Interview Tracking Log 86
Annex 16: Transcription Guide 87
Annex 17: Initial Ordering-Theme Code List 89
Annex 18: HUS Partner Roles & Responsibilities Matrix 90
REFERENCES 93

1. Background & Rationale

The World Health Organization (WHO) estimates a 50% reduction in malaria deaths since 2000 [1]. These reductions are attributed to the combination of vector control measures – including long-lasting insecticidal nets (LLINs), insecticide-treated nets (ITNs), and indoor residual spraying – and intermittent preventive treatment with antimalarial drugs in pregnant women and children. Despite considerable scale-up of these prevention interventions, malaria due to *Plasmodium falciparum* continues to be a major cause of morbidity and mortality. In 2016, WHO estimated that 216 million malaria episodes caused 445,000 deaths, the majority in young children in sub-Saharan Africa [2]. Access to LLINs, ITNs, and malaria medications remain limited in many communities [3-5]. Furthermore, insecticide and malaria drug resistance are growing concerns [6, 7]. New prevention tools are needed to advance malaria control efforts further.

RTS,S/AS01 (RTS,S), the world's first malaria vaccine,[1] is one such tool. RTS,S has been evaluated in a Phase 3 trial at 11 sites in seven sub-Sharan African countries [8, 9]. While Phase 3 results are promising, questions concerning the safety, efficacy, and feasibility of RTS,S introduction remain. The present study is part of a larger, multi-method evaluation designed to answer these questions.

1.1. RTS,S/AS01: Phase 3 Outcomes

1.1.1. Efficacy

RTS,S Phase 3 trials were conducted in children in two age groups (5-17 months and 6-18 weeks), comparing the control arm and groups receiving a three-dose and four-dose schedule [10]. As summarized in Table 1, vaccine efficacy declined over time in both age groups. Provision of a 4th dose in the six months following the 3rd dose improved efficacy substantially, particularly in the older age group. Vaccine efficacy was especially high in high-transmission areas. Compared to the control group, reductions in malaria-related hospital admissions, cases of severe anemia, and blood transfusion were also found and were greater in children who received four RTS,S doses.

1.1.2. Safety

Overall, no unexpected or concerning adverse events were observed in the Phase 3 trial [10, 11]. However, an increase in meningitis cases in the 5-17 month old group was reported. The relationship of these cases to RTS,S has not been established. Also, largely due to unexpectedly low female mortality in the control arm, all-cause mortality in female children was two-fold higher in the RTS,S arm compared to the control arm.

[1] GlaxoSmithKline (GSK) is the vaccine manufacturer and has led the development of RTS,S/AS01 over a 30-year period. A Phase 3 clinical trial was conducted between 2009 and 2014 through a partnership with GSK, the PATH Malaria Initiative (with support from the Bill & Melinda Gates Foundation), and a network of African research sites in seven countries.

Table 1: RTS,S Phase 3 Efficacy Findings [10]

Malaria incidents	Months following last dose	5-17 months old	6-18 weeks old
All episodes of clinical malaria	12 months	51.3%	32.9%
	18 months	45.7%	26.6%
	Trial end*: 3 doses	26.2%	18.2%
	Trial end*: 4 doses	39.0%	26.7%
All episodes of severe malaria	12 months	44.5%	38.5%
	18 months	37.5%	17.4%
	Trial end*: 3 doses	-2.3%	16.0%
	Trial end*: 4 doses	31.5%	26.7%
Malaria-related hospitalization	Trial end*: 3 doses	12.1%	13.2%
	Trial end*: 4 doses	37.2%	27.1%
Severe anemia	Trial end*: 3 doses	20.6%	12.8%
	Trial end*: 4 doses	31.5%	20.5%

* 48 months follow-up for 5-17 month old group and 38 month follow-up for 6-18 week old group.

1.1.3. Impact

By the end of the four-year trial period it was estimated that 1,774 malaria cases per 1,000 vaccines were averted among children ages 5-17 months who received all four RTS,S doses. Impact in high-transmission areas was estimated to be much higher: 6,565 cases averted per 1,000 vaccines [11].

In sum, the RTS,S Phase 3 trial showed that the vaccine can provide meaningful public health benefits when delivered alongside currently available prevention interventions.

1.2. Malaria Vaccine Implementation Programme (MVIP)

1.2.1. MVIP Objectives & the Healthcare Utilization Study (HUS)

Following a thorough review of data from the RTS,S Phase 3 trial by two independent WHO advisory groups — the Strategic Advisory Group of Experts on Immunization and Malaria Policy Advisory Committee — the WHO published a position paper calling for a pilot introduction of RTS,S in moderate-to-high transmission settings, accompanied by rigorous evaluations to address remaining concerns about the vaccine's safety and efficacy and the feasibility of providing a four-dose schedule [12]. The Malaria Vaccine Implementation Programme (MVIP) was established to conduct these evaluations [13]. Together, MVIP evaluation studies are designed to:

1. Further characterize vaccine safety in the context of routine immunization programs
2. Evaluate the vaccine's impact on mortality
3. Assess the programmatic feasibility of delivering a four-dose schedule requiring new immunization contacts in the context of health service delivery

The Dynamics of Healthcare Utilization in the Context of RTS,S/AS01 Vaccine Introduction (HUS) is a component of MVIP objective #3 concerning programmatic feasibility.

Table 2: Sample Vaccination Schedule Including RTS,S*

Vaccine / Age	Birth	Age in Weeks			Age in Months							
		6	10	14	5	6	7	9	12	18	22	24
BCG	X											
OPV	X											
DPT-HepB-Hib (penta)		X	X	X								
PCV		X	X	X								
Rota		X	X									
IPV				X								
MenA										X		
(M)R								X		X		
YF								X				
VitA						X			X	X		X
RTS,S:												
Ghana						X	X	X				X
Kenya						X	X	X				X
Malawi					X	X	X				X	

* Sample vaccine schedule from Ghana

Several critical questions are subsumed in MVIP objective #3: Can and how will delivery of four RTS,S doses be accommodated in typical, routine service delivery conditions and schedules (Table 2)? Are child caregivers motivated and able to bring children for all four doses, especially children in the second year of life and in light of partial protection? Does the uptake of RTS,S influence the dis/continuation or adoption of other malaria prevention and treatment behaviors? If so, how and why? How is partial protection conceptualized and understood both by vaccine clients and vaccine providers? How do these conceptions affect providers' support of the vaccine and communication about it with their clients? How do they influence clients' decisions to adopt RTS,S and to adhere to the four-dose schedule?

Complementing quantitative evidence gathered from health records, WHO Post Introduction Evaluations, and household surveys, the HUS will generate qualitative insight to respond to these and other critical questions specified below. WHO is overall lead of MVIP and is managing the quantitative evaluations. PATH is leading the HUS.

1.2.2. MVIP Countries

Following a call for expressions of interest issued by WHO in December 2015, Ghana, Kenya and Malawi were selected to participate in the pilot implementation of RTS,S. These countries were chosen based on their high coverage of LLINs, well-functioning malaria and immunization programmes, experience introducing new vaccines, and a high malaria burden even after scale-up of LLINs. Additionally, these countries are linked into existing infrastructure for immunization and malaria research, and they participated in the Phase 3 RTS,S vaccine trial.

As part of MVIP, RTS,S will be delivered in selected regions in each country by the countries' Expanded Programme on Immunization (EPI) programmes. National malaria control

programmes will ensure that existing WHO-recommended prevention tools, such as LLINs and artemisinin-based combination therapies (ACTs), continue to be deployed on a wide scale. The pilot is tentatively due to be launched in Ghana in September 2018, in Kenya January or February 2019, and in Malawi in October 2018.

1.2.3. HUS Research Partners

PATH released a call for proposals in 2017 to identify research partners to carry out the HUS in the three countries. The selected partners are shown in Annex 1. All the research teams have strong ties to Ministry of Health (MOH) programs and have significant experience conducting quantitative and qualitative health studies in their respective countries. In Kenya and Malawi, some of the HUS research partners were involved in the Phase 3 RTS,S trials and also have a role in MVIP's cross-sectional household survey.

2. HUS Purpose & Conceptual Foundations

2.1. Overall Purpose & Research Questions

The overall purpose of the HUS, as part of MVIP objective #3 focused on feasibility, is to inform RTS,S-related decisions, to clarify issues and options for effective introduction of RTS,S, and to identify ways to improve its provision and related health communications.

Using qualitative methods to systematically collect detailed accounts of people's experiences and perspectives, the HUS seeks in-depth understanding of contextual and behavioral factors impeding or facilitating RTS,S uptake. It builds on formative research exploring issues important for RTS,S introduction [14, 15], including in two MVIP countries: Ghana [16] and Kenya [17]. The HUS will address the following questions:

1. **Delivery and integration**: *How is RTS,S promoted, introduced, and delivered on the ground and what are the dynamics that shape these processes?*

   Principally through interviews with health providers working in vaccination, maternal and child health (MCH), and integrated management of childhood illnesses (IMCI) services, we will explore:

- provider perceptions about and understanding of RTS,S, including adverse events
- how the vaccine is being promoted
- how providers communicate RTS,S partial protection and messages about RTS,S four doses to their clients
- challenges and facilitators providing RTS,S and integrating its delivery with existing EPI, malaria, and MCH/IMCI services
- how and why providers' perceptions, attitudes and experiences related to RTS,S change over time

   Perspectives from health program managers and policy makers will supplement data collected from service providers, focusing on topics listed above as well as policy-level and planning issues.

2. **Community reception**: *How do community leaders, opinion shapers, community health workers and the general public learn about RTS,S, and how do they understand and use this information?*

   Through participant observation, individual interviews, and focus group discussions (FGDs) with various community groups we will explore:

- different communication channels through which communities learn about RTS,S
- what community leaders/members take away from their exposure to RTS,S messaging and how they, in turn, talk about RTS,S and promote or discourage uptake
- how community leaders/members (re)interpret RTS,S messages, particularly as relates to partial protection and the need to continue other malaria prevention behaviors
- how community leaders'/members' direct and indirect experiences with the vaccine and febrile/malaria episodes influence and change their perceptions about RTS,S
- how and why different community leaders/members encourage or discourage RTS,S uptake
- how and why community leaders'/members' perceptions and attitudes about RTS,S change over time

3. **Uptake and consequences**: *What socio-cultural and experiential factors influence RTS,S adoption and adherence? How and why? In what ways does RTS,S uptake impact other health behaviors?*

   Mainly through interviews with primary caregivers (PCGs) of RTS,S-eligible children (see Text Box 1, page 6) we will explore:

- *Malaria prevention and treatment*:
  - perceptions about malaria symptoms, risk, etiology and treatment efficacy
  - current malaria prevention and treatment behaviors for RTS,S-eligible and other children in the household
  - personal and household malaria experiences, probing severity, means of diagnosis, treatment resort and costs
  - how and why PCGs' perceptions, behaviors, and experiences related to malaria and RTS,S change over time
- *Immunization*:
  - perceptions about how immunizations work, their risks, and benefits
  - current vaccination status for RTS,S-eligible and other children (see Text Box 1, page 6) in the household
  - personal and household immunization experiences, probing satisfaction with and trust in the health service and vaccinations
  - how and why PCGs' perceptions, behaviors, and experiences related to immunization and RTS,S change over time
- *RTS,S*:
  - how PCGs learn about RTS,S and how they process and act on what they learn
  - PCG adoption of RTS,S (see section 2.3, page 6), probing for motivations/influences and description of experiences in the clinical encounter

- PCG adherence to the RTS,S four-dose schedule (see section 2.3 below), probing for motivations and influences and description of experiences in the clinical encounter
    - how and why PCGs' perceptions, behaviors, and experiences related to RTS,S adoption and adherence change over time
- *Interactions with the health system*:
    - PCG experiences in clinical encounters, with a focus on immunization services
    - provider-patient communication dynamics
    - PCG trust of health child health services

2.2. RTS,S Uptake Logic Model

The HUS design is predicated upon several assumptions about conditions and factors in the service delivery, household, and broader social environments that will lead to RTS,S adoption and adherence.[2] These assumptions are made explicit in the RTS,S Uptake Logic Model shown in Figure 1. The RTS,S Uptake Logic Model represents the study's overarching conceptual framework to structure data collection and analysis procedures. While allowing for inductive discovery through open-ended inquiry, this structure will help to keep our inquiry directed at specific MVIP knowledge needs and to maintain consistency across all three countries.

2.3. Critical Behavioral Outcomes

Alongside the textual data captured in ethnographic fieldnotes and in qualitative interviews, the study will track five critical behavioral outcomes in a sample of primary child caregivers:

i. **RTS,S Adoption**, defined as receipt of the first dose of the vaccine in the RTS,S-eligible child in the study household, indicated by receipt of dose 1 delivered by an EPI service provider[3]

ii. **RTS,S Adherence**, defined as receipt of two to three doses (partial adherence) of all four doses (full adherence) for the RTS,S-eligible child in the study household, indicated by receipt of doses by EPI service providers

iii. **Malaria Prevention**, defined as the use of an LLIN o ITN ("all the time," "some of the time," or "never") with the RTS,S-eligible child and other children ≤5 years old in the study household, indicated by PCG interview responses

Text Box 1: RTS,S Eligible and Other Children – definitions

RTS,S eligible child = a child whose caregiver has been enrolled in the study and who is at the appropriate age to receive RTS,S corresponding to the country's protocol:

- Dose 1: 5 or 6 months
- Dose 2: 6 or 7 months
- Dose 3: 7 to 9 months
- Dose 4: 22 to 24 months

Other children = all other children under the age of five years old living in the selected PCG's household

---

[2] Associated malaria prevention and treatment behaviors are subsumed in the Logic Model's references to partial protection. Although not explicitly indicated in the Logic Model, the HUS will also examine behaviors related to non-RTS,S immunization.

[3] We are not interested in determining whether or not a dose is "valid"; we are interested only in documenting if the RTS,S-eligible child receives the vaccine and when the dose(s) were received.

Figure 1: RTS,S Uptake Logic Model

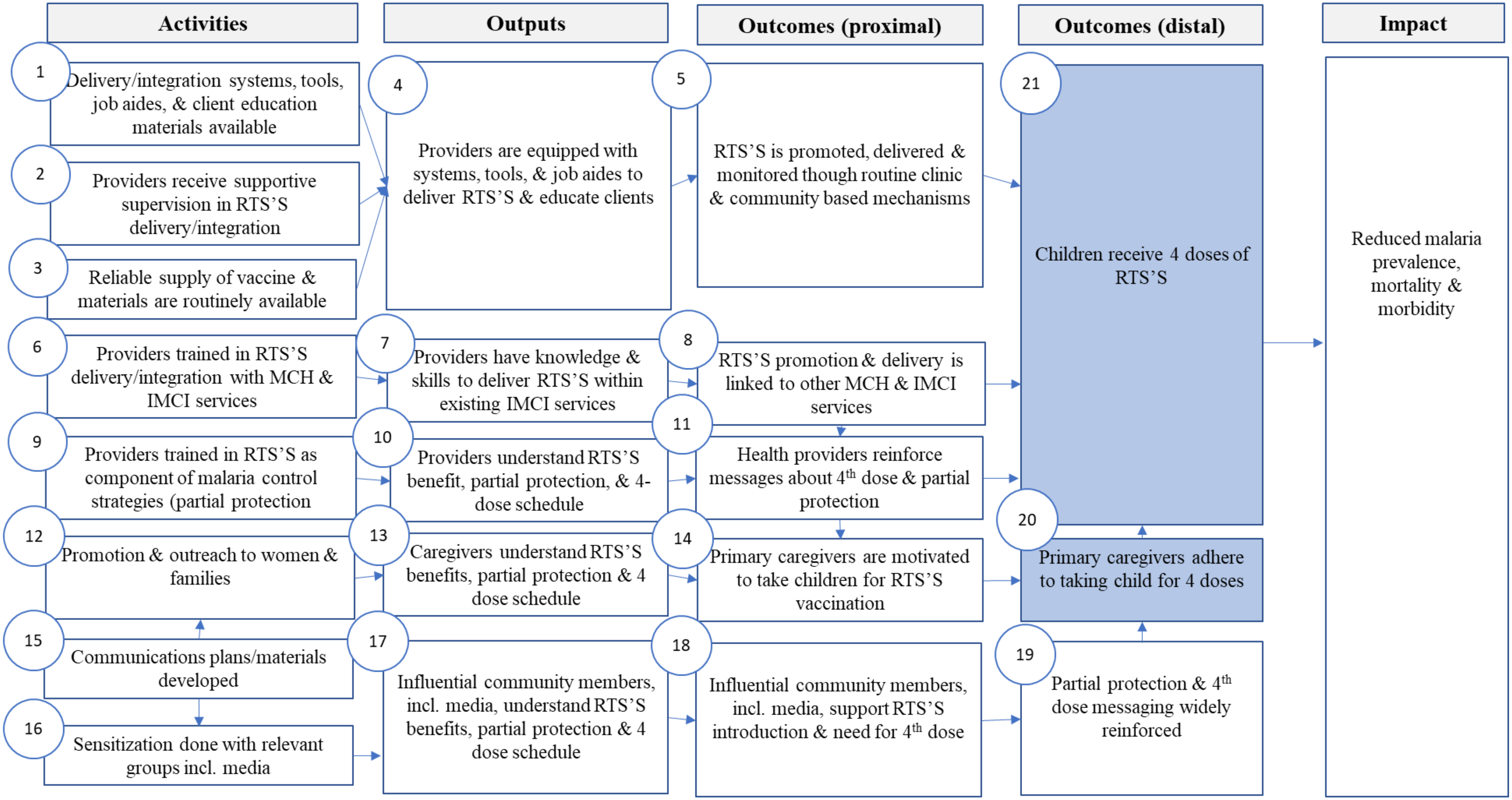

iv. **Malaria Care-Seeking**, defined as patterns/ timeliness of self-care and resort to popular, traditional, and biomedical sector services (see Text Box 2) in cases of fever or suspected malaria in the RTS,S-eligible child and other children ≤5 years old in the study household

v. **Routine Immunization Use**, defined as uptake of DPT3, MCV1, MCV2 and timeliness of receipt per national immunization schedule in the RTS,S-eligible child and other children in the study household

In conjunction with qualitative interviews, tracking these behavioral outcomes will enable us to understand individual PCGs' decision-making and behavioral trajectories and to empirically define and categorize behavioral pathways for description purposes and for qualitive comparison analyses.

Text Box 2: Resort to Care in Plural Health Systems – definitions

Self-care = treatment of a child determined by the PCG/other household members, including home remedies or pharmaceutical products purchased without prescription or consultation of a medical professional

Popular sector = resort to drug vendors and home remedy peddlers recognized neither by the formal/professional medical sector nor as a traditional practitioner inculcated or mentored in the traditional healing arts and practices

Biomedical sector = resort to MOH-recognized and accredited practitioners and service delivery facilities

2.4. Ecological Framework

An ecological model is built into the HUS study design. In public health and policy research, ecological approaches emphasize the interplay and inter-dependency between individual, interpersonal, organizational, and policy levels [18]. This framework has been used in formative research supporting vaccine introductions [19, 20] and is reflected in the HUS's data collection at multiple levels:

**Level:**	**Sample:**
Individual	Primary caregivers of RTS,S-eligible children
Interpersonal	Male household heads and female elders
Community	Local and religious leaders, opinion shapers
Institutional	Vaccine and IMCI service providers
Policy	Health sector leaders and program managers

Intentional examination of the inter-connections between these levels will shed light on contextual and dynamic factors shaping RTS,S delivery and uptake, helping to explain how and why its introduction in a particular community is succeeding or faltering.

2.5. Qualitative Panel Research

We draw on qualitative panel research (QPR) methods [21] in our data collection and analysis approaches to understand RTS,S introduction and uptake as a process shaped by changing contexts over time. In lieu of focusing on static factors and endpoints, QPR's longitudinal approach offers a powerful way for researchers to explore and chart dynamic processes as they occur. The method is inherently suitable for research on health interventions, such as RTS,S introduction, requiring prolonged and/or repeated involvement by patients and providers.

Table 3: Summary of HUS Fieldwork Timing Linked to RTS,S Introduction

Round 1		Round 2	Round 3
**Lead up to initial RTS,S delivery**	**Soon after dose 1**	**Mid-way between doses 3 and 4**	**Soon after dose 4**
	**5-6 months old**	**≈17 months old**	**22-24 months old**
Ethnographic immersion, individual interviews, and focus group discussions		Ethnographic immersion, individual interviews, and focus group discussion	Ethnographic immersion, individual interviews, and focus group discussions

Spanning one full, two-year RTS,S delivery cycle, HUS data collection is planned to occur in three rounds (Table 3 above): soon after the vaccine is first introduced and dose 1 is delivered (Round 1), mid-way between the delivery of doses 3 and 4 (Round 2), and soon after the fourth dose is provided (Round 3).

In addition to documenting the evolving context of RTS,S introduction over time, HUS findings will be used to inform communications and delivery strategies used in the introduction process. After each data collection round rapid analyses will be conducted in each country to share preliminary insights with national EPI programs, malaria programs, and the MVIP team, highlighting important perceptions, attitudes, behaviors and emergent issues.

3. Methods

The essential purpose of MVIP, including the HUS's contribution to the evaluation, is to produce evidence that will inform global recommendations on RTS,S introduction. Generalizing from the HUS's collective, cross-country findings is inherent in this purpose. Contrary to the frequent characterization of qualitative research as ungeneralizable, analytic and case-to-case generalization is widely held as a goal of qualitative studies [22-26]. Qualitative meta-syntheses [25] and multisite approaches [27] emerged specifically to enhance generalizability of qualitative findings. In addition to general guidance for high quality qualitative research [28-30],[4] our methods and analysis procedures also take into account specific recommendations for multi-site/multi-case qualitative assessments [22, 25, 27].[5]

Balancing the need for methodological comparability with site-specific (or case-specific) concerns is a major challenge in both qualitative meta-syntheses and multisite studies. And so it is for the HUS as well. While we apply the same or similar methods across the three countries, we also allow country-specific variation to fit different contexts and research partner preferences. Across the three countries we apply strong methodological consistency in three areas:

[4] Key quality criteria include: (1) clarity and transparency in methodological logic and procedure; (2) systematicity and consistency in approach, especially in multisite studies; (3) procedural rigor in sampling, data collection, and analysis; (4) sample representativeness, including a clear rationale for purposive criteria and commitment to utilizing these criteria through to data analysis; (5) transferability/generalizability and relevance of study findings; and (6) reflexivity, referring both to iterative processes and awareness of researcher influence on outcomes.

[5] Ensuring cross-site and procedural comparability, maintaining a manageable project scale, and achieving a balance of site-specific versus combined reporting/analysis are emphasized for multisite research and meta-synthesis.

i. we will address the same research questions, focusing on the same interview topics and using same interview guides whenever possible
ii. we will use the same sampling strategy to select study sites (HUS communities)
iii. will use the same sampling and interviewing procedures used in primary child caregivers

While there is significant methodological overlap in the other study groups (including health professionals and various community members), research partners have proposed slightly different selection and data collection strategies for these groups. Alongside country-specific background information, Annexes 2-4 provide details on the different approaches proposed. As we work as a team to refine our procedures and tools in preparation for the study's launch, we will share approaches and adopt, whenever possible, the same or similar approaches and tools across the three countries.

3.1. Sampling

To facilitate data pooling and comparison of findings across countries, research sites, and sub-groups, we utilize a parallel sampling strategy [22, 23] in all three countries. This stratified sampling frame is intended to maximize contextual variation [31] at the country level while applying a systematic approach to the selection of HUS study sites in all three countries (Figure 2).

Figure 2: Sampling Strategy at the Country Level

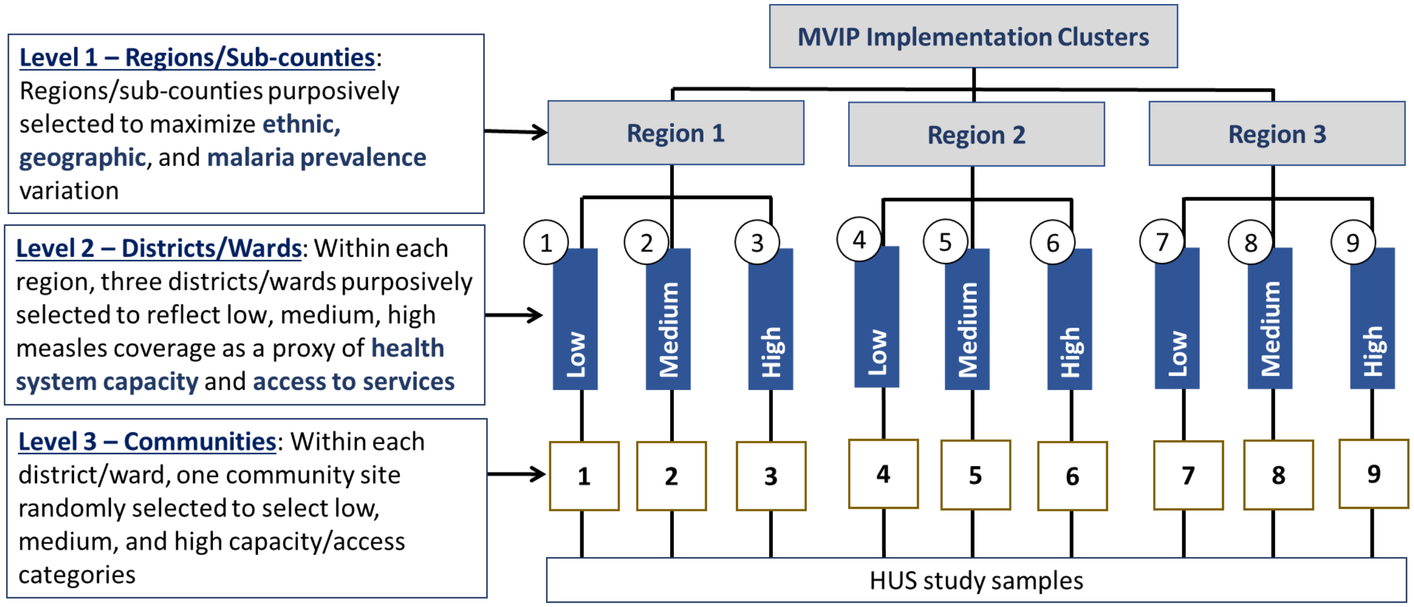

HUS study sites, shown as Level 3 in Figure 2, will be individual communities, each representing relatively homogenous social units. Except for interviews with health leaders and managers working at national, regional and district levels, participant recruitment, interviewing, and ethnographic immersion will occur in the communities selected for inclusion in the HUS.

3.1.1. Community Sites

Definition
Conceptualized in their own right as cases to be empirically described and compared within and across the three countries, HUS communities are central to the study design. In all three countries, community sites will parallel geographic areas covered by primary health care services.[6,7,8] These areas refer to the smallest administrative unit or collection of units aligned with services provided by at least one health facility and supported by community outreach teams. We expect community population sizes to range from approximately 2,000-5,000. Communities which are not served by an MOH-affiliated health facility will be excluded from the study. Private and religious facilities that are recognized by the MOH as part of their delivery structures and national plans will be included.

Sample Size
Herriott and Firestone [27] include the number of sites studied in multisite qualitative research as a major "design issue," citing a tension between having many sites to enhance generalizability while ensuring that enough study resources (time, funding) are available for proper analysis for each site and for cross-site/case comparison. Reflecting our best judgement of a community sample that is neither too large to permit "deep, case-oriented analysis" nor too small to reveal "new and richly textured understanding of experience" [23, 31, 32 p 183], the HUS will include nine community sites/country for a total of 27 communities across all three countries.

Selection Procedure
As depicted in Figure 2, community sites in the HUS will be selected from MVIP clusters where RTS,S is being introduced. Within the MVIP clusters, three administrative regions (Level 1) will be intentionally selected to capture socio-cultural, geographic, and malaria prevalence variation found in the clusters.

At the sub-regional level (Level 2) we will use measles coverage rates as a proxy indicator of health system capacity and access to services. Measles coverage is defined by Gavi, The Vaccine Alliance as a direct measure of "immunization and health system strength," since it measures the ability of preventive health services to reach infants later in the first year of life.[9] Within each Level 1 region we will select three districts/wards to represent "low," "medium," and "high" measles coverage. Districts will be selected in consultation with country MOHs, taking into account Level 1 criteria as well as other programmatic and contextual factors relevant to EPI programmes and MVIP implementation.

Within each Level 2 district/ward selected, one Level 3 community will be randomly selected. Level 3 communities in each district/ward will thus include one community with relatively "low" health system capacity and access, one with "medium" health system capacity and access, and one with "high" health system capacity and access, as indicated by variable measles coverage rates. Definitions and values of "low," "medium," and "high" measles coverage rates at the

---

[6] https://dhsprogram.com/pubs/pdf/SPA6/02chapter02.pdf
[7] http://kmhfl.health.go.ke/#/home
[8] http://www.aho.afro.who.int/profiles_information/index.php/Malawi:Analytical_summary_-_Service_delivery
[9] http://www.gavi.org/library/gavi-documents/stratey/definition_of_performance_indicators-for-GAVI-strategy-2011-2015

district level will be determined after reviewing available data and consulting with country EPI teams.

3.1.2. Study Groups: Overview

Reflecting the HUS ecological approach, in each community site we will conduct individual interviews of varying depth (IDIs) and focus group discussions (FGD) in multiple study groups: primary child caregivers; health leaders, managers, and providers; and community leaders and members. Some individuals and groups will be interviewed only once, while others will be interviewed at two more data collection rounds.

Table 4 on the following page lists the HUS's different study groups by country and data collection round, indicating the following for each group:

1. Sample type – cohort of cross-sectional
2. Interview type – IDI or FGD
3. The target and maximum sample size for each group, specifying individuals who:
   - are initially **recruited** into the study
   - **replace** individuals initially recruited in cohort samples (e.g., health workers who move to other jobs), if needed
   - are **added iteratively** based on emergent issues and study findings

For FGDs, the number of groups and individuals/group is also indicated in Table 5 (next pag).

3.1.3. Primary Child Caregivers (PCGs)

Cohort & Cross-Sectional Samples

In each HUS community, two samples of PCGs of RTS,S-eligible children (see Text Box 2, page 6) will be recruited. One cohort sample of 5 individuals/community and 45/country will be followed for 22-24 months to collect data at three points in time during one full cycle of RTS,S doses, resulting in three in-depth interviews for each PCG in the cohort sample.

The first interview with the PCGs in the cohort sample will occur soon after dose 1 is initially provided (Round 1); the second interview will be held mid-way between doses 3 and 4 (Round 2); and the final interview will happen soon after dose 4 is provided (Round 3) (see Table 4).

Table 5: PCG Cohort and Cross-sectional Samples by Data Collection Round

Data collection round	Round 1	Round 2	Round 3
**Point in RTS,S schedule**	**Soon after dose 1**	**Mid-way between doses 3 & 4**	**Soon after dose 4**
**Child's age**	**5-7 months**	**≈17 months**	**22-24 months**
Target Sample/Country	***N***	***N***	***N***
Cohort	45	45*	45*
Cross-sectional			45
Total/country	***45***	***45***	***90***

* Same individuals from previous round(s)

Table 5: HUS Study Groups, Sample and Interview Types, and Sample Sizes by Country and Data Collection Round

Level	Country	Groups	Sample & Interview Type		Detail	Sample Size							
						Round 1		Round 2		Round 3		Total	
						Target	Max	Target	Max	Target	Max	Target	Max
National/Regional	Ghana	Vaccine impl sub-committee Chairs	x-sect	IDI	recruited:	5	5					5	5
		Regional Health Service leaders	x-sect	IDI	recruited:	5	5	5	5	5	5	15	15
	Kenya	National malaria, EPI, & MVIP mgrs	cohort	IDI	recruited:	7	7			7	7	7	10
					replaced:						3		
		County malaria, EPI, & MVIP mgrs	cohort	IDI	recruited:	5	5			5	5	10	12
					replaced:						2		
					added iteratively:					5	5		
	Malawi	National malaria, EPI, & PMI mgrs	cohort	IDI	recruited:	3	3			3		3	5
					replacement:						2		
District & Service Delivery	Ghana	District Health Management Teams	cohort	FGD	groups (1/district):	3	3			3	3	9	9
					individuals/group:	≤10	≤10			≤10	≤10	≤10	≤10
					total individuals:	30	30			30	30	30	33
					replaced (1 ind/group):						3		
		Sub-district Health Teams (** some communities will fall into the same sub-district)	cohort	FGD	groups (varies**):	6	6			6	6	9	9
					individuals/group:	≤10	≤10			≤10	≤10	≤10	≤10
					total individuals:	60	60			60	60	60	66
					replaced (1 ind/group):						6		
		Community Health Nurses	cohort	FGD	groups (1/community):	9	9			9	9	9	9
					individuals/group:	≤10	≤10			≤10	≤10	≤10	≤10
					total individuals:	90	90			90	90	90	99
					replaced (1 ind/group):						9		
	Kenya	Subcounty health mgt team staff	cohort	IDI	recruited:	7	7	7	7	7	7	7	12
					added iteratively:			3	3	2	2		
		MCH/IMCI health center staff	cohort	FGD	groups (1/community):	9	9	9	9	9	9	9	9
					individuals/group:	≤10	≤10	≤10	≤10	≤10	≤10	≤10	≤10
					total individuals:	90	90	90	90	90	90	90	108
					replaced (1 ind/group):				9		9		
	Malawi	EPI, malaria, env. health district mgrs	cohort	IDI	recruited:	12	12			12		12	15
					replaced:						3		
		Under-five clinic health center staff	cohort	FGD	groups (1/community):	9	9			9	9	9	9
					individuals/group:	≤12	≤12			≤12	≤12	≤12	≤12
					total individuals:	108	108			108	108	108	117
					replaced (1 ind/group):						9		

Table 5: HUS Study Groups, Sample and Interview Types, and Sample Sizes by Country and Data Collection Round (cont)

Level	Country	Groups	Sample & Interview Type		Unit	Sample Size							
						Round 1		Round 2		Round 3		Total	
						Target	Max	Target	Max	Target	Max	Target	Max
Community Groups	Ghana	Leaders, opinion shapers	x-sect	IDI	recruited:	18	18			18	18	36	36
		Male household heads	x-sect	FGD	groups (1/community):	9	0			9	9	18	18
					individuals/group:	≤10	≤10			≤10	≤10	≤10	≤10
					total individuals:	90	90			90	90	180	180
		Female elders	x-sect	FGD	groups (1/community):	9	0			9	9	18	18
					individuals/group:	≤10	≤10			≤10	≤10	≤10	≤10
					total individuals:	90	90			90	90	180	180
		Traditional healers, drug vendors, etc.	x-sect	IDI	recruited:	18	18			18	18	36	36
	Kenya	Gatekeepers, opinion shapers	x-sect	FGD	groups (1/community):	9	9			9	9	18	18
					individuals/group:	≤8	≤8			≤8	≤8	≤8	≤8
					total individuals:	72	72			72	72	144	144
		Male household heads	x-sect	FGD	groups (1/community):	9	9			9	9	18	18
					individuals/group:	≤7	≤7			≤7	≤7	≤7	≤7
					total individuals:	63	63			63	63	126	126
		Female elders (iterative sample)	x-sect	FGD	groups (1/community):			9	9	9	9	18	18
					individuals/group:			≤8	≤8	≤8	≤8	≤8	≤8
					total individuals:			72	72	72	72	144	144
		Traditional healers (iterative sample)	x-sect	IDI	added iteratively:			18	18	18	18	36	36
	Malawi	Village heads, religious leaders	cohort	IDI	recruited:	18	18			18	22	18	22
		Male household heads	x-sect	FGD	groups (1/community):	9	9			9	9	18	18
					individuals/group:	≤12	≤12			≤12	≤12	≤12	≤12
					total individuals:	108	108			108	108	216	216
		Female elders	x-sect	FGD	groups (1/community):	9	9			9	9	18	18
					individuals/group:	≤12	≤12			≤12	≤12	≤12	≤12
					total individuals:	108	108			108	108	216	216
		Traditional healers, drug vendors	cohort	IDI	recruited:	18	18			18	22	18	22
Individual	Ghana	Primary child caregivers	cohort	IDI	recruited:	45	63	45	63	45	63	45	63
			x-sect	IDI	recruited:					45	45	45	45
			iterative	IDI	added iteratively (2/comm):				18		18		18
					Ghana total	*45*	*63*	*45*	*81*	*90*	*126*	*90*	*126*
	Kenya	Primary child caregivers	cohort	IDI	recruited:	45	63	45	63	45	63	45	63
			x-sect	IDI	recruited:					45	45	45	45
			iterative	IDI	added iteratively (2/comm):				18		18		18
					Kenya total	*45*	*63*	*45*	*81*	*90*	*126*	*90*	*126*
	Malawi	Primary child caregivers	Cohort	IDI	recruited:	45	63	45	63	45	63	45	63
			x-sect	IDI	recruited:					45	45	45	45
			iterative	IDI	added iteratively (2/comm):				18		18		18
					Malawi total	*45*	*63*	*45*	*81*	*90*	*126*	*90*	*126*
					**HUS total**	**135**	**189**	**135**	**243**	**270**	**378**	**270**	**378**

A separate cross-sectional sample of PCGs with children old enough to have received the 4th dose (22-24 months) will be recruited at Round 3. Five PCGs/community and 45/country will be recruited into the cross-sectional sample.

Combining cohort and cross-sectional samples, our target PCG sample size is 10/community, 90/country, and 270 across the three countries (Table 6). A sample of size of 10 PCGs/community falls within recommended ranges (≥6 and ≤10 individuals) for qualitative phenomenological studies to achieve adequate information redundancy while remaining a manageable size [33].

Table 6: Target PCG Sample Size Per Country

District	District 1			District 2			District 3			Total
**Community**	**1**	**2**	**3**	**4**	**5**	**6**	**7**	**8**	**9**	
Cohort	5	5	5	5	5	5	5	5	5	**45**
Cross-sectional	5	5	5	5	5	5	5	5	5	**45**
**Total/community**	**10**	**10**	**10**	**10**	**10**	**10**	**10**	**10**	**10**	**90**
Total/country	***10***	***20***	***30***	***40***	***50***	***60***	***70***	***80***	***90***	

In anticipation of individuals dropping out or who are lost-to-follow-up, we will recruit seven PCGs/community into the cohort sample to achieve our target of having at least 45 PCGs/country who complete interviews at all three data collection rounds. For the cross-sectional sample, we will recruit PCGs into the study until we reach our target sample of five/community and 45/country.

PCG Selection Procedure

PCGs will be identified from “eligible households” in the community, defined as households with at least one child who meets RTS,S-eligibility criteria at the time of study enrollment, i.e., 5 to 6 months old for the cohort sample and 22 to 24 months old for the cross-sectional sample depending on the country’s RTS,S schedule (see Annexes 2-4). Eligible households will be identified using WHO’s EPI Sampling Technique,[10] involving selection of starter and subsequent households. In each community we will randomly select the starter household and follow the EPI Sampling Technique to identify subsequent households.

Study staff will approach each selected household to determine if an RTS,S-eligible child is living in the household. If yes, the researchers will consult adult household members to identify the child’s caregiver. PCGs who are ≥15 years old will be invited to participate in the study; PCGs younger than 15 years old will be excluded from the study. Only one PCG/household will be recruited for the study.

If multiple RTS,S-eligible children are living in the household, the researchers will apply an agreed-upon random selection procedure to determine which PCG will be invited to participate in the study. PCGs who express interest in participating in the study will be given time (1+ days), if needed, to confirm her/his decision. After this time, if the PCG agrees to participate, s/he will be enrolled per the procedure outlined in the next section. If s/he declines to participate, researchers will invite another PCG from within household (if multiple RTS,S-eligible children

[10] EPI Coverage Survey, Expanded Programme on Immunization, WHO (pp 15-18).

live there) or move to the next household following the EPI Sampling Technique. If there are no RTS,S-eligible children living in the household, the researchers will follow the EPI Sampling Technique to select the next household and repeat the procedure.

In both the cohort and cross-sectional samples, PCGs who do not have children of RTS,S-eligible age and PCGs who are <15 years old will be excluded from the study.

Cohort Sample Follow-up
Whenever possible, at the time of enrollment researchers will obtain a cell phone contact to schedule follow-up interviews. At the second interview, participants will confirm or update their cell phone contact. Prior to the follow-up site visit, study staff will attempt to reach participants through a phone call or text message to establish a date and time for the interview. If after repeated attempts a PCG cannot be reached by phone, the research team will proceed with alternative strategies to reach her/him, including a home visit. In some rural areas where cell phone ownership is sparse, the phone contact may be the number of a local research assistant or health worker who can liaise with the PCG. When a cell phone contact is not available, locally-placed research staff will follow up with the PCG in person.

3.1.4. Health Leaders, Program Mangers, & Providers

The intention of interviews with health personnel is to understand issues and challenges around RTS,S delivery occurring in the formal, biomedical sector. Therefore, samples of health personnel will include individuals working in this sector. Recognizing, however, the plural nature of health systems and health seeking [34], popular (e.g., drug vendors) and traditional (e.g., traditional healers) sector perspectives will be captured through community samples.

Although sampling strategies with health leaders and managers will vary slightly across countries to reflect differences in health system structures (see Annexes 2-4), in all the countries health personnel representing different levels and roles in the health system will be included in the study (Table 7). Though not anticipated, we will exclude health leaders under the age of 18 years old. Other than the age exclusion, no health leaders meeting the criteria below will be excluded from the study.

Table 7: Level and Role of Health Personnel Selected by Country

Level	Ghana	Kenya	Malawi
National	All five chairs of Ghana’s vaccine implementation subcommittees	National malaria, EPI, and MVIP program managers	National malaria, EPI, and President’s Malaria Initiative managers
Regional	From each region:<br>1) health service directors<br>2) community health officers/nurse supervisor<br>3) disease control officers<br>4) health information officers<br>5) malaria focal points	From each county: malaria, EPI, and MVIP program managers	

Level	Ghana	Kenya	Malawi
District/ ward	1) District Health Management Teams<br>2) Subdistrict Health Teams	Subcounty health management team	District EPI, malaria, and environmental health managers
Facility	Community Health Nurses	MCH/IMCI health center staff	Under-five clinic health center staff

At the national, subnational and district levels, all individuals serving in the roles specified in Table 7 will be invited to participate in the study. At the facility level, all staff working the community's health center(s) in the service areas indicated will be invited to participate in FGDs. In the selected study sites, except for individual under the age of 18 years old, no health staff working at the subnational and district level meeting these criteria will be excluded from the study. In primary health facilities in the community sites, no health providers meeting these inclusion criteria will be excluded from the study.

Appropriate approvals from national, regional, district and facility level managers and supervisors will be secured prior to inviting any health personnel to participate in individual interviews or FGDs. The target number of participants in each role by country is shown in Table 4 and included in Annexes 2-4.

3.1.5. Community Leaders, Opinion Shapers, & Others

Community leaders, male household heads, female elders and various other community members will be interviewed in each country in the course of the HUS. With slight differences in approach, the selection of community participants will be informed principally by community entry activities and ethnographic engagement prior to and throughout the study period.

Table 8: Community Members to be Interviewed by Country

Member	Ghana	Kenya	Malawi
**Leaders, opinion-shapers** (village elders, chiefs, head teachers, community health volunteers, pastors)	Identified through ethnographic fieldwork and approached after building rapport.	Identified through community entry activities with local authorities and traditional leaders.	Identified through ethnographic fieldwork and approached after building rapport.
**Male household heads**	Identified and invited to attend a HUS FGD by community leaders.	Identified and invited to attend a HUS FGD by community leaders.	Identified by local research staff, health workers or through ethnographic engagement.
	Intentionally selected to represent different ages and from a range of social, economic and caregiving positions (e.g. integration in social networks, household economic status and number of children).		
**Female elders** (women with grandchildren)	Identified and invited to attend a	Identified through community health volunteers.**	Identified by local research staff, health workers or through

Member	Ghana	Kenya	Malawi
	HUS FGD by community leaders.		ethnographic engagement.
	Intentionally selected to represent a range of social background, household economic status, and number of children.		
**Others** (traditional healers, pharmacists and drug vendors, local NGO staff)	Identified through community entry activities and ethnographic field work.	Identified through ethnographic fieldwork and approached after building rapport.**	Identified through ethnographic fieldwork and approached after building rapport.

** Interviews are contingent on findings in Rounds 1 and 2.

Country teams will observe appropriate cultural, social and political protocol before reaching out to prospective study participants. Details on the target number of community participants, interview timing, and interview format are provided in Table 4 and Annexes 2-4.

In all community samples, individuals under the age of 18 years old will be excluded from the study. Male household heads who are unmarried, living alone or have no children will be excluded from the study. Female elders who do not have grandchildren will be excluded from the study. Other than age, no explicit exclusion criteria will be applied to other various community members that may be identified through ethnographic field work.

3.1.6. Iteratively-derived Samples

The HUS's mandate to produce generalizable findings to inform global policy requires us to maintain strong systematicity and consistency in sampling and data collection procedures across the countries. At the same time, iterative approaches that encourage data collection strategies based on emergent findings is an important strength of qualitative research. It is especially pertinent to the HUS given the study's explicit interest in observing change over time after RTS,S is introduced.

Through ongoing analysis we anticipate discovering phenomena that merit deeper exploration. Examples of issues that could necessitate interviewing more individuals or the same individuals multiple times include:

- Rumors about RTS,S or negative events occurring in a community that may affect vaccination-seeking
- Experience of RTS,S-related adverse events following immunization (real or perceived) that may hinder vaccine uptake
- Specific issues in a healthcare setting that impair delivery of the vaccine
- Individual-level factors in households or PCGs that appear to facilitate/obstruct vaccine uptake and that require fuller understanding

Having the flexibility to collect data in additional samples and/or interviews will strengthen the HUS's contribution to MVIP.

3.2. Data Collection Procedures

The procedures described below and observation, interview, and FGD guides included in Annex 5-9 will be collaboratively reviewed and further refined prior to start of data collection. The interview formats and topics will not change. Some questions in the guides may be deleted, improved upon, or added based on careful review and feedback received from colleagues in country MOHs, WHO, and partner organizations.

3.2.1. Ethnographic Immersion

Ethnographic methods complement formal interviews and other data collection techniques with insights gained through direct personal involvement in the community, [35]. Rather than maintaining distance through formal procedures, the value of ethnography derives from the researcher's informal engagement, as participant, in people's everyday worlds. Ethnographic data come from insider experience from the researcher actively participating in interpersonal encounters and social events. Unlike, however, ordinary participation in social life, the ethnographic researcher engages in social activities with heightened awareness, intentionally observing and documenting details about interactions and context [36]. This dual participant-observer purpose is the cornerstone of ethnographic inquiry.

In the HUS, research staff will conduct participant observation at various events, settings, and community gatherings pertinent to RTS,S introduction as well as through informal conversations in natural social settings. This approach of simply "being there" will allow research team members to immerse themselves in community life and to gain a feel for local concerns and attitudes that directly or indirectly relate to RTS,S and its uptake.

Ethnographic immersion will be particularly important during the period when RTS,S is first introduced to the communities. Over the four weeks before vaccine introduction, research teams will observe and participate in events such as health staff induction, planning meetings, and community mobilization activities. To monitor how RTS,S messaging is initially being received, the first interviews with health providers and community representatives will be conducted near the time of these events. Intensive immersion is planned for data collection Rounds 2 and 3 but will occur throughout the study period.

Country teams will follow the Participant Observation & Fieldnote Guide shown in Annex 5.

3.2.2. PCG Interviews

Informed Consent

Interviews with PCGs will be scheduled after obtaining the caregiver's informed consent. Informed consent will be obtained prior to each interview with caregivers enrolled in the cohort sample.

In the consenting process, participants will be informed that interviews will be audio-recorded in full. Because it is crucial that we accurately capture the details of caregiver perceptions, experiences, and decision-making rationale in their own words, a caregiver's refusal to be audio-recorded will be treated as refusal to participate in the study.

Interview Place, Duration, & Language

Each interview will last approximately one to 1.5 hours and will be held in a private place at the PCG's home or at another private location preferred by and convenient for the participant. Interviews will be conducted in the local language or English per the participant's preference.

PCG Profiles
At the start of each interview, research assistants will collect data on socio-demographic characteristics of the participant, her/his household, and immunization status and bed net use for the RTS,S-eligible child and, as possible, other children living in the household (see Text Box 1). The PCG Profile Sheet shown in Annex 6 will be used for collecting this data.

To obtain information on the children's vaccination status, interviewers will ask to see the children's vaccination cards. If the PCG cannot produce a vaccination card for the RTS,S eligible-child, the research team will determine the child's immunization history at a later date by examining vaccination registries kept by the EPI program or health facility.

In addition to asking open-ended questions about malaria prevention practices used with the RTS,S-eligible child and other children in the household, the interviewer will ask to see where the children sleep. If bed nets are not visible, s/he will probe to see if bed nets are set up nightly and will ask to see where they are kept during the day.

A new PCG Profile Sheet will be completed at each encounter with cohort participants.

In-depth Interview Questions
PCG IDIs will focus on the topics listed on pages 5-6 using the interview PCG Interview Guides shown in Annex 7. Whenever possible, we will use question-types and probes that invite participants to recount their personal experiences, strategies and rationales underlying attitudes, decisions, and actions. Questions in cohort and cross-sectional participants will be similar, but the latter necessarily more retrospective in nature.

3.2.3. Health Leaders, Managers, & Providers Interviews & FGDs

Health professionals working at various levels in the health system will be interviewed in all three countries covering the research questions listed on page 4. Interviews and FGDs will specifically address the topics outlined in Table 9.

Table 9: Interview Topics with Health Leaders, Managers, and Providers

<table>
<tr><th>Leaders</th><th>Managers/Supervisors</th><th>Providers</th></tr>
<tr><td colspan="3">Appropriate to individuals'/groups' role in the health system, elicit perspectives in relation to:
• RTS,S provision around the six health system building blocks: human resources, financing, supply chain management, governance, and health information systems
• Challenges, experiences, and progress in introducing RTS,S
• RTS,S efficacy, safety, and potential impact</td></tr>
<tr><td>Additionally explore:
• Policy considerations
• Planning, timing issues</td><td>Additionally explore:
• Program-specific concerns and challenges
• Planning, timing issues</td><td>Additionally explore:
• Service delivery and integration challenges and</td></tr>
</table>

Leaders	Managers/Supervisors	Providers
		recommendations for improvements<br>• Client communication, focusing on partial protection<br>• Client willingness/ability to come for all four doses
IDI	IDI or small groups	FGD
≈30 min/interview	≈30 min/interview	≈1 hour/FGD

Health leaders and managers will be interviewed individually while service providers will be asked to participate in FGDs. Both individual interviews and FGDs will be audio-recorded for later transcription.

Basic information about each provider participating in the study, including her/his professional role and years of experience, will be recorded followed by open-ended questions addressing topics listed in Table 9. FGDs will be led by a skilled moderator with an observer/note-taker recording group dynamics and interview highlights to supplement audio-recordings and transcriptions. Health leaders and managers will be interviewed by the country PI, co-PI or another senior member of the research team.

Annexes 8 is FGD guide to be used with health providers. Health leader and manager interview guides will be broadly based on this guide but modified to reflect different leaders/managers positions in the health sector.

3.2.4. Community Members Interviews & FGDs

Community members will be interviewed in all three countries covering the research questions listed on pages 4-5. Interviews and FGDs will address the topics outlined in Table 10.

Table 10: Interview Topics with Community Members

Leaders, Opinion-shapers	Male Household Heads	Female Elders
Appropriate to the individual/group being interviewed, elicit perspectives in relation to:<br>• Malaria, probing perceived risk, severity, prevention and treatment<br>• Vaccination, probing perceived value and satisfaction with services<br>• RTS,S, probing awareness, communication channels, popular interest/acceptance, and rumors		
Additionally elicit:<br>• Suggestions to enhance RTS,S communications and implementation	Additionally explore:<br>• Malaria- and vaccine-related experiences in their households<br>• Men's role in determining child health decisions and behaviors (prevention	Additionally explore:<br>• Malaria- and vaccine-related experiences in their households<br>• Elder women's role in advising younger mothers on child health matters

Leaders, Opinion-shapers	Male Household Heads	Female Elders
	actions, treatment- and vaccination-seeking), generally and specifically related to malaria, vaccination, and RTS,S	and the specific advice they provide in relation to malaria prevention and treatment, child vaccination, and RTS,S
IDI or FGD	FGD	IDI or FGD
≈30 min/interview or FGD	≈1 hour/FGD	≈1 hour/interview or FGD

Our interest in interviewing community leaders, such as village heads, chiefs, head teachers, community health volunteers and religious leaders, stems both from their influential role in shaping community attitudes and from insight they can provide into issues of concern in their communities.

Both playing an important role at the interpersonal level shaping child health decisions and actions within the household, male household heads and female elders will be interviewed to explore these influences in depth. Male household heads and female elders will not be selected from the same households as the PCGs.

Other community members to be interviewed, such as traditional healers, local NGO staff, pharmacists and drug vendors, will be determined once the study teams have gained deeper understanding of community-specific contexts and issues through ethnographic observations and interviews. Interviews with these individuals will address the core topics listed in Table 10 as well as specific issues relevant to their social roles.

Individual interviews and FGDs with community members will be audio-recorded for later transcription.

Basic information about each individual participating in the study will be recorded followed by open-ended questions. FGDs will be led by a skilled moderator with an observer/note-taker recording group dynamics and interview highlights to supplement audio-recordings and transcriptions.

See Annex 9 for community member interview guides.

4. Data Management

4.1. Record Keeping

Linked record keeping systems at the country- and global-level will be set up to keep track of HUS datasets and data collection progress. Data logs will record details on the country; sample and participant/FGD group unique IDs; interview type, place and date; transcription, translation, and quality check status; and audio-, transcription file names. Country teams will maintain the following data logs:

- PCG interviews (Annex 10)
- Individual health provider interviews (Annex 11)

- Health provider focus groups (Annex 12)
- Individual community leader/member interviews (Annex 13)
- Community leader/member focus groups (Annex 14)

The HUS global team will collate country logs into master logs to keep track of study progress and to produce summaries of the number and types of interviews completed.

4.2. Transcription & Translation

Audio-recorded interviews will be transcribed by the country teams following the transcription template shown in Annex 15. Interviews conducted in the local language will be transcribed in the local language and subsequently translated and transcribed into English. Whenever possible, the interviewers themselves will transcribe/translate audio-recordings as soon as feasible after the interview takes place, ideally within 72 hours of the interview. Translation from local language into English will be literal when meanings are equivalent. When, however, linguistic structures, concepts, and terms are divergent, the translation procedure will be to capture as accurately as possible the intended meaning of the respondent.

Translation quality checks will be built in to the data management process but especially intensive after Round 1 interviews. Designed as on-the-job training, after Round 1 data collection, at least one full interview will be reviewed for translation quality for each interviewer. This initial translation quality check will include all translator-transcriber research staff and involve reading the English translation while listening to the audio-recorded interview. Translation inaccuracies will be discussed and, as needed, the English translation modified. The collective results of Round 1 translation checks will be shared with all transcriber-translators and used to guide subsequent translation practices.

Routine transcription quality checks will involve a study investigator reading the full transcript for clarity and anonymity. When needed, researchers will return to the audio-recordings to clarify passages and modify the English translations in the transcripts when appropriate.

4.3. Categorized & Coded Data

To facilitate data quality checks and inter-rater reliability procedures, coded and thematically grouped textual data will be shared between the country and global teams using NVivo, a qualitative data analysis program. Country codes will be compiled routinely and shared to update the HUS master code list.

4.4. Quantitative Data

Data on socio-demographic and other variables derived from close-ended questions will be entered into an Excel spreadsheet along with details on the participant/FGD group unique IDs and transcript file names and numbers. At the country level, entered data will be verified for accuracy at least once prior to sharing with the HUS global team.

4.5. Backup & Archiving

Audio-files will be named and numbered following an agreed-upon file naming convention and archived by country research teams. At the end of each interview day, audio-files will be

uploaded and saved to study computer with a backup copies saved to a study computer and an external drive. Also using an agreed-upon naming convention, final English-translated transcripts, along with associated cover sheets, will be archived both by country teams and by the HUS global team. Quantitative datasets will be similarly named, filed, and archived.

Section 8 describes steps to data sharing through open access data repositories.

4.6. Confidentiality & Ethics

Informed consent procedures will include a description of intended data use, sharing, and archiving. Research assistants will anonymize the transcripts in the process of transcribing the interviews. As noted above, routine transcript quality control measures will include checking for anonymity. Transcripts and quantitative data will be identified through unique participant identification numbers, de-linked from personal identifiers and contact information. Files linking identification numbers and personal information will be kept under lock and key and accessible only to key study staff in charge of managing data collection and participant follow up.

5. Data Analysis

Beyond description of context, attitudes, beliefs, and behaviors, the HUS is interested in causal explanation of community acceptance and uptake of RTS,S. To this end we follow realist evaluation principles and practical strategies to identify causal influences in qualitative data [37]. The RTS,S Uptake Logic Model (page 7) depicts key assumptions around factors and relationships important for RTS,S introduction and provides a conceptual roadmap guiding our analytical steps and focus.

The steps below to organize, interrogate, and interpret our findings only represent the start of data analysis. Further elaboration and refinement of the procedures, overall and for the different study groups, will be data-driven and determined through collaborative exchanges and iterative data engagement.

5.1. Categorizing through Thematic Analysis

Broad data ordering

An initial "horizontal" reading of the interviews will allow us to structure the data across cases into broad categories reflecting the study's principal areas of interest. Annex 16 summarizes these initial data-ordering themes.

Open-coding & reduction

Subsequent open coding within these categories will focus on: (i) clarifying sub-themes/sub-codes (e.g., negative vs positive social support), (ii) inductively identifying additional themes (descriptive, conceptual, or temporal in nature), and (iii) isolating critical events recounted by study participants.

Critical events will include pivotal experiences, incidents, and interactions that influence participants' attitudes about, understanding of, and behaviors related to RTS,S uptake. Isolating critical events as a thematic category will allow us to subsequently investigate the events in depth – to understand the circumstances and context of the event, to observe how the event plays

out over time, and to identify consequent attitudinal and behavioral shifts important to RTS,S. Critical events themselves will be coded and grouped according to their nature.

As our understanding of the data matures and topical themes and critical events are identified, the data will be (re)grouped and systematically condensed into broader meaning units [38, 39]. Meaning units derived through the deductive, inductive and iterative steps summarized here provide building blocks for additional analytic steps to connect, validate, and compare data(sets) across time and across study (sub)samples.

5.2. Connecting Themes Within Cases, Across Time, & Across Datasets

Diachronic case construction: individuals, groups, and communities

Whereas thematic analysis breaks stories, experiences, and events apart into conceptually bounded meaning units, vertical reading of the data within cases and across time is an integrative exercise leading to more complete diachronic pictures [40, 41]. From the data, we will construct behavioral and contextual trajectories of change for individuals, groups, and communities:

- PCGs – Case portraits will be developed from interview data collected in fieldwork Rounds 1, 2, and 3 for PCGs in the cohort sample. Chronological pathways based on retrospective accounts will be constructed for PCGs enrolled in the cross-sectional sample.
- Groups – Case portraits for health providers and other community groups will track thematic changes revealed in the interviews over the course of the study.
- Communities – Portraits to characterize and describe the community reaction to RTS,S as the vaccine is introduced and integrated into the health service mix will be constructed by linking themes and significant events captured in ethnographic fieldnotes and interview transcripts.

  Linked themes and events from different study groups and data sources will help to reveal important interactions at work in the community. This process of linking themes and events represents a key step in analysis of ecological connections and community systems.

Typologies of change: pen portraits and case profiles

From textual and quantitative data, highly condensed “pen portraits” [42]will capture the chronological highlights of each case (individuals, groups, communities). Pen portraits will help us to grasp core themes and emergent patterns in the data and to keep track of multiple case chronologies in the sample. For example, RTS,S dose adherence patterns for PCGs will be tracked and annotated with short descriptors of context and rationales underlying the observed pattern.

Linked to the pen portraits will be fuller descriptive case profiles organized by fieldwork rounds (or chronologically-ordered retrospective accounts). Descriptive case profiles will preserve many details of how a case unfolds. Thematic headings of chronologically-ordered data in these profiles will help us create “through lines” in the accounts, which can be pulled out for detailed examination [43, 44].

Ordered data in pen portraits and case profiles will facilitate further data reduction and cross-case comparison, finally resulting in the creation of typologies of change. Typologies of change will include individual behavioral pathways related to key variables of interest in the study as well as broader contextual changes observed at the group and community level.

5.3. Causal Explanation

Closely linked to the thematic, within-case, and temporal analyses described above, we will follow a procedure to explicitly identify mechanisms linking RTS,S introduction to the observed outcomes in our study. Our approach will follow realist evaluations used to inform health policy [45, 46]. The process used by Van Belle et al [21] illustrates:

- Step 1: assess the behavioral results of the intervention
  – RTS,S adoption, adherence, malaria prevention behaviors, and diachronic typologies of change
- Step 2: systematically examine the data for causal mechanisms associated with the observed results
  – following open-coding, consensus, and reduction procedures described above
- Step 3: ascertain and describe the influence of contextual factors that facilitate or impede intervention uptake
  – following open-coding, consensus, and reduction procedures described above

5.4. Coding Consistency Within & Across Research Sites

To facilitate project management and promote consistency across country sites, a global analysis team will be comprised of at least one team member from each country and PATH's HUS PI and data manager. This coding team will develop within-country and across-country procedures to determine inter-rater reliability agreement. In-country team members will ensure that inter-rater reliability procedures are followed and will participate in across-country deliberations. At the global level PATH staff will coordinate across-country deliberations and will compile, update and share revised code definitions and lists.

5.5. Exemplary Quotes

Throughout all stages of data analysis, teams will identify text units that exemplify various themes, typologies, and mechanisms highlighted in the study and code them as **exemplary** in NVivo. Exemplary quotes audio-coded in Nvivo allowing for easy retrieval and translation accuracy checks prior to use in presentations and publications.

5.6. Validating Interpretations & Comparing (Sub)-samples

Inadequate attention to discrepant evidence (e.g., cases that do not fit researchers' impressions and interpretations of the data) represents a common threat to validity in qualitative studies [37]. To avoid this, we will assess the presence/absence of key themes, behavioral variables, mechanisms, contextual factors, and empirically-derived typologies across cases to determine overall patterns in the data. "Quantitizing" textual data to determine and visualize patterns [47] will help validate our interpretations and compare findings across sub-samples, communities and countries. Purposively selected sub-samples will be systematically compared [28], also aided by this quantitizing procedure.

5.7. Country- & Global-Level Analysis Roles

Table 11 below summarizes data analysis roles at the country- and global levels.

Table 11: Country-level & Global-level Data Analysis Roles

Step/Purpose	Country-level	Global-level
Thematic analysis	• Conduct initial data ordering<br>• Conduct inductive coding within themes<br>• Generate exhaustive, reduced code lists<br>• Share code files w/ HUS-global	• Provide initial theme list<br>• Compile code lists<br>• Generate, update, and share master code lists
Connecting analysis	• Create pen portraits<br>• Create detailed case profiles<br>• Categorize into typologies of change<br>• Share coded files w/ HUS-global	• Compile typologies of change<br>• Generate, update, and share master list of typologies
Causal explanation	• Assess behavioral outcomes<br>• Code mechanisms and contexts<br>• Share coded outcome-mechanism-context data	• Compile outcome-mechanism-context data<br>• Generate, update, and share master code list
Consistency	• Ensure country-level inter-rater reliability procedures are followed and documented<br>• Participate in global procedures	• Coordinate and document across-country inter-rater reliability procedures<br>• Adjust and share definitions/code lists as needed
Exemplary quotes	• Identify exemplary quotes through textual and audio-codes<br>• Ensure translation accuracy checked prior to use in presentation and publication	• Ensure translation accuracy checked prior to use in presentation and publication
Validating & comparing	• Determine presence/absence of agreed-upon themes and variables	• Coordinate process to determine themes, variables to quantitize<br>• Maintain master datafile

6. Study Timing & Management

6.1. Fieldwork Timing

The RTS,S introduction dates remain uncertain. However, planned introduction dates are September 2018 for Ghana, October 2018 for Malawi, and January or February 2019 for Kenya. Table 12 shows a timely for study preparation, data collection, and analysis by country. We will adjust this timeline to actual launch dates as needed.

6.2. Coordination & Roles

This HUS protocol embraces recommended best practices for managing large-scale qualitative studies, including a research design and procedures that maintain a strong focus on the project's research questions; methodical and consistent data collection procedures; and iterative team-

based coding and analysis [48, 49]. Successful implementation of these practices will require strong coordination and frequent team communication [50].

Timescales and milestones
HUS global will maintain and share a master workplan for the team indicating key milestones, responsible persons, and timeline. We will use PATH's project management tools to facilitate the work planning process.

Face-to-face communication
In addition to holding at least one all-partner workshop annually, we will ensure face-to-face communication between country and HUS global team members through strategically timed site visits (e.g., the first round of data analysis). We will maximize face-to-face communication by identifying opportunities to meet around scheduled travel and at conferences that multiple HUS team members are likely to attend.

Team-based coding
HUS global and country investigators will ensure in-depth training of research assistants before and during data coding. These sessions will ensure both wide participation in the analysis process and a high level of consistency across countries. The structure and process to ensure coding consisting across countries and sites is described in section 5.4 above.

Frequent, ongoing communication and standing quarterly meetings
Via email, Skype, and phone, the HUS global team will ensure frequent communication and updates with country PIs and teams to ensure timely progress, clarify/develop study procedures, identify and resolve bottlenecks, and address country-specific issues. Additionally, we will hold standing quarterly meetings with individual country teams and with all HUS partners.

Annex 17 summarizes roles of key staff on PATH's and the research partner teams.

Table 12: HUS Study Timeline and Anticipated RTS,S Introduction Dates by Country

	Main Activities	1	2	3	4	5	6	7	8	9	10	11	12	13	14	15	16	17	18	19	20	21	22	23	24	25	26	27	28	29	30	31	32	33	34	35
		Project Month																																		
Pre-study	Fieldwork prep, staff training																																			
Pre-study	Pre-study ethnographic obs.																																			
Pre-study	Ongoing ethnographic obs.																																			
Pre-study	Dose 1																																			
Round 1	Formal R1 data collection																																			
Round 1	Doses 2 & 3																																			
Round 1	R1 data preparation																																			
Round 1	R1 preliminary analysis																																			
Round 1	R1 insights workshop/report																																			
Round 2	Formal R2 data collection																																			
Round 2	R2 data preparation																																			
Round 2	R2 preliminary analysis																																			
Round 2	R2 insights workshop/report																																			
	Dose 4																																			
Round 3	Formal R3 data collection																																			
Round 3	R3 data preparation																																			
Round 3	R3 preliminary analysis																																			
Round 3	R3 insights workshop/report																																			
Final analysis and write-up																																				

Anticipated RTS,S Introduction Dates (as of 9/21/2018)

Ghana: early February 2019
Kenya: April 2019
Malawi: late March 2019, following planned pre-launch in late February/early March

6.3. Staff Training

Prior to conducting interviews or observations in the field, country research teams will conduct an HUS training for research assistants, which will cover the following topics:

- Malaria and immunization, including basic epidemiological and coverage data for the country as well as the country's EPI and malaria program strategies and goals
- RTS,S, including its public health potential, dose schedule, and partial protection benefits
- MVIP implementation and how the HUS fits in
- HUS study objectives and overall design
- Communications, including the national crisis communication plan, HUS crisis communication procedures, procedures to field questions about RTS,S and to refer participant to health professionals
- Interviewing skills
- HUS study procedures
- Research ethics, incorporating e-certification into the training and covering HUS-specific procedures and including a session focused on ethics in the context of ethnographic engagement

When available, PATH staff will participate in research staff training, including members of the HUS global team and/or PATH country office staff (vaccine program focal points and communications officers). A pre- and post-test training evaluation will be administered to assess effectiveness of the training and to identify supplemental training needs.

6.4. Crisis Communication

HUS researchers are likely to encounter vaccine safety-related events in the course of conducting interviews and observations in community sites. WHO defines a vaccine safety-related event as "any event that can negatively affect a vaccination programme."[11] Two safety event categories are pertinent to HUS:

- **Real or perceived adverse events** following immunization (AEFI), which can lead to a loss of confidence in the vaccine and the vaccination program

  In interviews with PCGs we will collect information on real of perceived AEFIs related to RTS,S vaccination. These findings (including frequency, distribution, and nature) will be reported to national EPI, health communications, and MVIP managers following each data collection round. Interviewers will be trained to refer participants to health services when AEFIs are reported in the interview process.

  Any descriptions of very serious AEFIs believed to be associated with RTS,S that research staff hear about in interviews or elsewhere will be escalated up per the escalation flow outlined in Table 13.

[11] WHO presentation on Vaccine Safety Events: Managing the Response

- **Negative media reports or rumors**, which may be factual, partly factual, anecdotal or completely untrue, and may be reported in the media, interpersonally, or through social media

  Stories and rumors that receive little or no public attention, that do not play on emotions and fears, and that are not plausible are defined by WHO as "low" impact media reports and rumors. We will report findings on these stories and rumors following each data collection round, summarizing their nature, frequency, and distribution.

  Stories and rumors that appear to be getting public attention, that trigger emotional fears, and that appear to be plausible will be reported following the escalation flow shown in Table 13.

Table 13: Escalated RTS,S Safety-related Event Reporting Flow

Who reports?	Reports incident to?	What information is reported?
Study staff	Site supervisor	• Date<br>• Location<br>• Nature<br>• Context<br>• Specific concerns raised, actions taken<br>• How the event was observed (e.g., in an interview, participant observation)
Site supervisor	Country PI and study manager	
Country PI or manager	Simultaneously to the following PATH staff:<br>• In-country focal point<br>• In-country communications officer<br>• HUS PI<br>• HUS study coordinator	
PATH in-country focal point or communications officer	As appropriate to:<br>• Country RTS,S Crisis Communications Team<br>• Country RTS,S Technical Safety Team<br>• MVIP manager	

To prepare and support HUS study staff in identifying and reporting on RTS,S safety events, HUS staff in each country will be invited to participate in the crisis communication training (around August 21018) and will be provided the final MVIP Crisis Communications Plan (June-July 2018) and any updates made to these plans. Crisis communications will be included in initial and refresher training with interviewers and other field-based study staff, supported by PATH communications staff if possible.

7. Ethical Procedures

HUS principal investigators, co-investigators, and data managers will have completed ethics training for research on human subjects prior to project start up. Research staff training will incorporate e-certification of ethics training on research with human subjects and will cover specific procedures to be used in HUS study groups. No research activities with research participants will begin until we receive final Certification of Approval from PATH's Research

Ethics Committee and all other country and institutional ethics committees that need to approve this protocol prior to data collection.

7.1. Informed consent

General procedure

Informed consent will be obtained from participants prior to or at the time of enrollment into the study. Most often, consenting will occur at the place of the interview and will be conducted by the research staff conducting the interview or FGD. Consenting will take place in a private setting, including only the research staff and the participant and witness (when present).

The consenting procedure will include a verbal description of the purpose of the HUS, the nature of questions that will be asked, the approximate time it will take to complete the interview or FGD, how the data will be collected (i.e., audio-recorded and note-taking), and how the data will be used, stored, and shared. Individuals will also be informed that they can ask questions about the study and the process at any time before, during, or after the interview.

Prospective participants will be given time to read the consent form and pose questions. Consent forms will be read aloud to prospective participants if they prefer.

Consent will be obtained in the language of choice by the participant. Primary caregivers included in the cohort sample will be re-consented at each interview. Written consent will be obtained whenever possible, indicated by signature on a separate signature page.

Participants will be provided a copy of the consent form and the signed signature page. Signature pages kept by the research team will be under lock and key accessible only to the country PI, co-PIs, and key management-level staff.

Individuals in cohort samples

Individuals participating either in individual interviews of FGDs multiple times will be reconsented prior to each interview or FGD.

Non-literate participants

Non-literate participants will be consented in the presence of a witness chosen by them. Prior to proceeding with the consent process, the research assistant will verbally explain the role of the consent witness in the presence of both the witness and prospective study participant. The research assistant will clarify the witness's role to help ensure full comprehension of the study and its procedures by the prospective participant and will emphasize that the final decision to participate in the study or not rests with the participant. The consent form will then be read aloud, and the witness will also be allowed time to read the form. Participants and witnesses will both be given an opportunity to pose questions.

The participant's consent will be indicated by signature-thumbprint on the signature page alongside the witness's name and signature.

PCG minors
For PCGs who are minors, we will obtain parental consent and the PCG's assent. If the selected household is a child-headed household, we will observe national regulations for mature or emancipated minors.

Ethnographic fieldwork
In carrying out ethnographic fieldwork we will observe ethical guidance for anthropological research embraced by the American Anthropological Association and the European Commission, DG Research and Innovation. [12] While respecting the norms and principles of research on human subjects, these institutions also recognize the value of ethnographic research and the need to obtain consent from participants in the least disruptive manner possible in naturalistic social encounters. The steps described below are intended to achieve an appropriate balance between providing participants enough information about the study for them to freely choose to participate and fostering trust and rapport between researcher and participant:

- Public announcements through community leader communications and community meetings: In advance of and throughout the course of the study community leaders will be fully briefed on the study objectives, procedures, duration, and status. We will encourage community leaders to share basic information about the study with the larger community and request to attend community meetings to announce the study and address community member questions. Communications about the HUS will include mention of study staff placed in the community to observe pertinent events and interactions and to engage community members in open conversations about child health, malaria, and the new vaccine.

- Public announcements associated with RTS,S launch communications: Collaborating with EPI, health communications, and MVIP colleagues on planning committees and technical working groups, HUS partners will work to incorporate messaging about the HUS study into RTS,S health provider training sessions and into general public sensitization and communications' campaigns. Whenever possible, a HUS staff will participate in these events to present the study and to address any questions. Communications about the HUS will include mention of study staff placed in the community to observe events and interactions and to engage community members in open conversations about child health, malaria, and the new vaccine.

- Consent through ongoing engagement and transparency: Ethics training for study staff will include a session focused on ethical considerations and practices suitable for ethnographic methods. In this session, we will engage trainees in a process of developing guidelines for and practicing naturalistic ways to inform participants in ethnographic encounters about the study. Key information to be conveyed will include that HUS study staff are: (i) informally observing and interviewing people in the community to learn about their views on child health, malaria, and the new RTS,S vaccine; (ii) recording information about the different viewpoints they hear, but are not recording any

[12] http://www.americananthro.org/ParticipateAndAdvocate/Content.aspx?ItemNumber=1652 (accessed 7 August 2018); http://ec.europa.eu/research/participants/data/ref/h2020/other/hi/ethics-guide-ethnog-anthrop_en.pdf

identifying information about individuals or groups; and (iii) being transparent about the study to allow people to choose if they wish to share and participate or not.

7.2. Risks

Participation in the HUS poses minimal risk to research participants, though some individuals may experience discomfort answering certain questions. A participant may not have complied with a recommended health behavior (such as a mother not using a bed net where her child sleeps) or fear that they do not fully understand a health message received (such as a health provider not fully recalling partial protection messaging), thus causing her/him to feel embarrassed during the interview.

In obtaining informed consent we will acknowledge the potential of this discomfort and emphasize that individuals are free to decline answering any question they choose or to withdraw from the study at any time they choose, without negative consequence to them.

7.3. Benefits

HUS findings will provide important information in support of decisions and strategies in vaccine, malaria, and health communications' programs.

The communities involved in this study stand to benefit from evidence-based recommendations and strategies informed by HUS findings.

7.4. Confidentiality

Personal identifiers will be de-linked from study identifiers and datasets. Lists linking study identification numbers to names and contacts will be kept under lock and key accessible only to the PI and key study management staff who need to coordinate follow-up visits. In the process of transcription, identifying information will be removed. Only anonymized datasets will be shared.

In the consenting process for FGDs, we will include a statement indicating that their identity will be known by other discussants and that the researchers cannot guarantee that others in the group will respect confidentiality. We will ask all participants in the FGD to keep all comments made during the discussion confidential and not discuss what happened during the FGD outside of the meeting. This message will be restated prior to starting the FGD.

7.5. Compensation

Modest compensation may be provided to participants to reimburse them, as needed, for any costs incurred (e.g., bus fare) to participate in the study. Consistent with research norms in the individual countries and in compliance with national ethics guidelines, the following compensation is proposed for the countries:

Table 14: Interview Compensation Proposed by Countries

Group	Ghana	Kenya	Malawi
PCGs	Transport costs*<br>Snack	Transport costs*<br>Bath bucket	Transport costs*<br>$10/interview**
Health personnel	Transport costs*<br>Snack (w/ providers)	Transport costs*	Transport costs*
Community leaders	Transport costs*<br>Snack	Transport costs*	Transport costs*<br>$10/interview**
Male household heads	Transport costs*<br>Snack	Transport costs*<br>Bath bucket	Transport costs*<br>$10/FGD**
Female elders	Transport costs*<br>Snack	Transport costs*<br>Bath bucket	Transport costs*<br>$10/FGD**
Others	Transport costs*<br>Snack	Transport costs*<br>Bath bucket	Transport costs*<br>$10/interview**

* Reimbursed costs
** $10/interview is required by Malawi's national ethics committee

8. Dissemination & Future Data Use

8.1. Dissemination

Select findings and highlights will be shared throughout the course of the study with the countries' EPI programs, malaria programs, and health communications teams, allowing them to use findings to improve delivery and refine messaging. We also will share findings intermittently with the wider MVIP team, calling attention to any issues important for their investigations.

Final results will be presented in a close-of-project PATH report and shared in various forms by country teams and PATH staff at national, regional, and international conferences and through publication in peer review journals.

In the final field visit to communities, preliminary highlights from the study will be shared with local leaders and community members. Community leaders will be alerted to the study's final write-up and publication.

8.2. Future Data Use

Data storage and use by research partners
Anonymized data will be stored by HUS research partners for further analysis and publication for a period indefinitely. MVIP partners, including PATH, HUS research partners, country MOHs, and the WHO will have full access to the anonymized transcripts and coded data and will be encouraged to lead country-specific or global (re/additional)analysis and publications. Publications by MVIP partners will not require additional ethical review. If an MVIP partner wishes to lead a comparative analysis using HUS data from other countries, we will request that the appropriate country PI(s) and PATH HUS staff have an opportunity to contribute to or review the manuscripts prior to publication.

Open access data storage and use
Per funder requirements, we will make anonymized data open access using a health science or social science data repository, such as the Open Science Framework (https://osf.io/), the Harvard Dataverse (https://dataverse.org/), or the Open ICPSR (https://www.openicpsr.org/openicpsr/). The repository will be selected after careful review of repository options and their different platforms, requirements, and user bases.

Information about specific community sites will be removed from open-access datasets. Individual participants and FGDs will be identified exclusively by unique study ID numbers. As described in Section 4, transcripts will be anonymized and quality-checked for anonymity prior to coding, archiving, or depositing in a repository. One repository will include data from all three countries, accessible in country-specific files.

Terms of future data use for non-MVIP researchers will include:

- A requirement for ethical review by the researcher's IRB/ethics committee and by country ethics committees prior to publishing or printing findings or new analyses.
- PATH review of presentations and publications using HUS data.
- Proper reference to and citation of the study.

Annex 1: HUS Research Partners

Ghana Partners

Prime Institution:	University of Health and Allied Sciences
Partnering Institutions:	University of Cape Coast<br>The Kwame Nkrumah University of Science and Technology<br>University of Energy and Natural Resources
Principal Investigator	Dr. Margaret Gyapong
Team Members	Evelyn Korkor Anash<br>Seth Owusu Agyei<br>Kofi Awusabo-Asare<br>Ellis Owusu-Dabo<br>Phedelia Doegah<br>Arti Singh<br>Samuel Fosu Gyasi<br>Alison Krentel

Kenya Partners

Prime Institution:	Liverpool School of Tropical Medicine
Partnering Institutions:	London School of Hygiene and Tropical Medicine<br>Kenya Medical Research Institute<br>US Centers for Disease Control and Prevention<br>KEMRI Welcome Trust Research Programme (KWTRP), Kenya
Principal Investigator	Dr. Jenny Hill
Team Members	Feiko ter Kuile<br>Jayne Webster<br>Meghna Desai<br>Nelli Westercamp<br>Brent Wolff<br>Simon Kariuki<br>George Okello<br>Caroline Jones

Malawi Partners

Prime Institution:	Malawi-Liverpool-Welcome Trust Clinical Research Programme
Partnering Institutions:	University of Malawi, College of Medicine<br>Liverpool School of Tropical Medicine
Principal Investigator	Dr. Nicola Desmond
Team Members	Kate Gooding<br>Linda Nyondo-Mipando<br>Anja Terlouw<br>Neil French

Annex 2: Ghana Background

1. MVIP & HUS Regions

In Ghana, MVIP will be implemented in Volta, Central, and Brong Ahafo regions, where the number of malaria cases is very high. According to the Ghana Health Service, 2016 Annual Report,[13] all three regions report rates of malaria above the national average of 363 cases/1,000 population. All three regions also include districts with the highest number of un-immunized children.

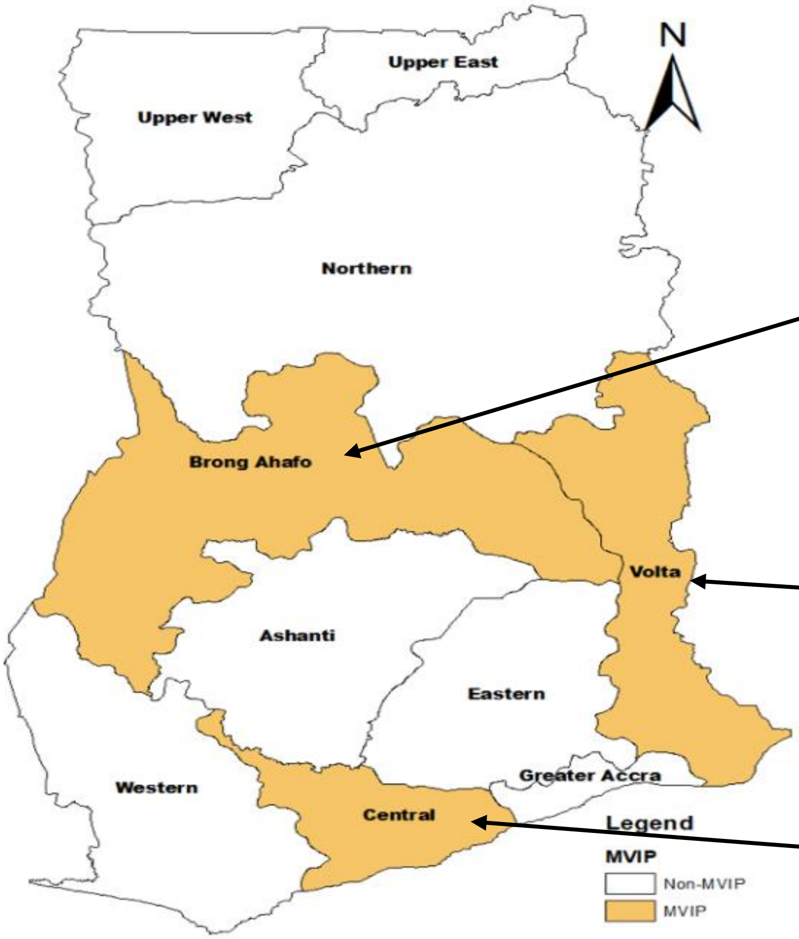

Brong Ahafo

districts: 27
health facilities: 568
Total population: 2,728,484
Primary languages:

- Twi
- Akan
- Mo
- Hausa

Volta

districts: 25
health facilities: 519
Total population: 2,491,284
Primary languages: Ewe

Central

districts: 20
health facilities: 356
Total population: 2,486,904
Primary languages:

- Akan
- Fante

The three regions each have at least 20 districts, similar population sizes, and a large number of health facilities. Selection of districts to be included in the HUS will be undertaken in collaboration with Ghana's EPI Programme. In addition to English, study languages are will include two regional languages: Akan and Ewe.

[13] Ghana Health Service, 2016 Annual Report (https://www.ghanahealthservice.org/downloads/GHS_ANNUAL_REPORT_2016_n.pdf)

Volta Region

The Volta Region is located in the Eastern part of the country and is divided into three natural geographical belts namely the southern, middle and the northern belts. The majority of people in the region live in the rural areas (66.3%); “overbank” settlements along the Volta Lake and the lagoons in the southern parts of the country are especially difficult to access. Over 70% of the population speak Ewe and the main economic activities are fishing and farming.

Brong Ahafo Region

The Brong Ahafo Region (BAR) is the second largest region in Ghana covering 39,557 square kilometres. BAR has 27 administrative districts, with Sunyani as the regional capital. Situated in the forest savannah transitional zone in the middle belt of Ghana, more than half of the population lives in rural communities. The predominant ethnic group in the region is Akan, except for Pru, Sene and Kintampo North where the dominant ethnic groups are Guan, Gurma, and Grusi respectively. Principal livelihoods in BAR including farming and small-scale mining.

Central Region

The Central Region derives its name for the Central Province of the Gold Coast. The current regional capital, Cape Coast, was the capital of the Gold Coast up to 1877, when it was moved to Accra. Nineteen districts in the region spread across two broad ecological zones, coastal savannah and interior forested areas. In the coastal savannah districts, the predominant livelihood activities are fishing and farming, while in the forest areas farming dominates followed by some mining. Predominantly Fante speaking, the cosmopolitan nature of Central Region makes it a cultural melting pot. Forty-seven percent of the population lives in urban areas.

2. Health System Structure

Ghana’s health service delivery is organized at three levels – national, regional and district. The district level is further divided into a number of sub-districts and also incorporates a community-

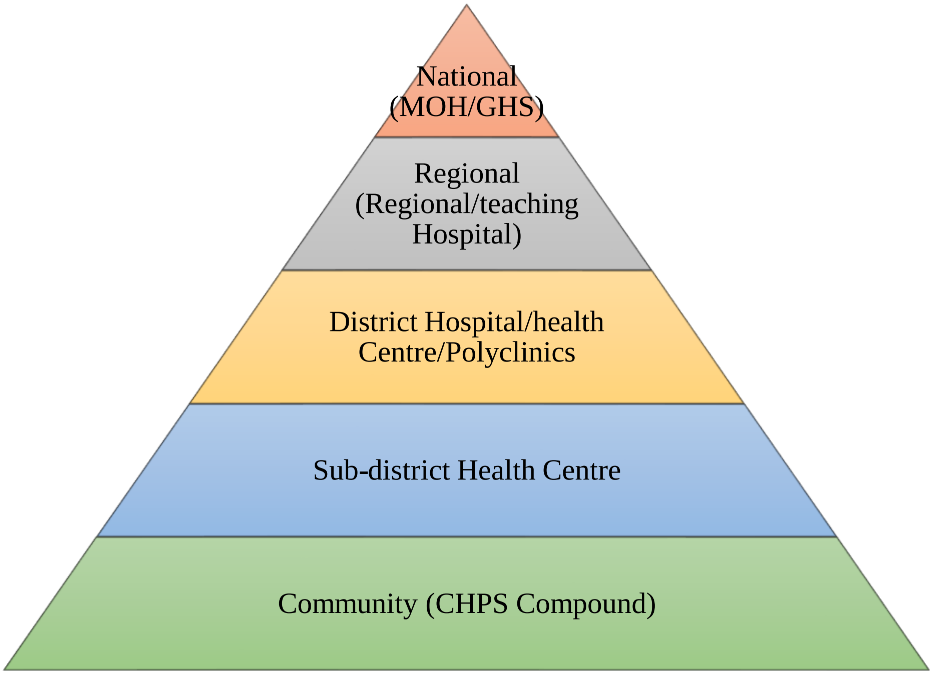

level health delivery system. Depicted in the pyramid below, Ghana's public health services are delivered through a hierarchy of hospitals, health centers, maternity homes, clinics, and Community-based Health Planning and Service units. Civil society organizations also play a considerable role in delivering health services in Ghana, particularly in community mobilization and community-based immunization. Ghana is making progressive improvements in the health status of the population, in spite of being confronted with the double burden of non-communicable and infectious disease across all ages and sexes. Non-communicable diseases are becoming the major causes of morbidity and mortality alongside the existing and emerging communicable diseases.

2.1. Malaria Burden & Control

Malaria is perennial in all regions of Ghana, except in the country's three northern regions (Upper East, Upper West and Northern Regions). Approximately 60% of Ghanaian children live in areas where the *Plasmodium falciparum* parasite prevalence (PfPr) is greater than 30%; throughout the country, PfPr is higher than 10%. The MOH estimates that around 774,000 infants currently survive beyond the neonatal period annually. In the proposed MVIP regions, the estimated total number of children expected to survive the neonatal period of 120,000.

Ghana malaria control efforts have increased dramatically over the past 10 years. Insecticide treated net use increased from 10% in 2005 to 55% in 2014. Rapid diagnostic testing and ACT use has also increased. Testing is required prior to treatment with ACTs at all health facilities and at community dispensary shops. MOH 2016 data show the proportion of febrile children who took any ACTs to be around 41%. Seasonal malaria chemoprophylaxis is being piloted in the Upper West Region and expected to be scaled up to Upper East and Northern regions, where malaria burden is seasonal.

Significant progress made in implementing key malaria control efforts positions Ghana well for evaluating the impact of RTS,S in the context of routine service delivery and continuation of existing control efforts.

Ghana has chosen to administer four doses of RTS,S vaccine using a schedule of 6, 7, 9 and 24 months of age. The minimum interval between all vaccine doses is 1 month.

3. Sampling, Data Collection, Data Management & Ethical Procedures

Overall, the Ghana HUS team will follow the sampling, data collection, and ethical procedures spelled out in this protocol. Specifically, procedures described earlier in this document in relation to the activities listed below will be used in Ghana:

1. The overall sampling strategy to select regions, districts, and community sites
2. The sampling strategy to select households and PCGs
3. Interview, FGD, and observation guides included in other annexes
4. Ethics procedures to ensure:
    a. Informed consent
    b. Voluntary participation
    c. Privacy and confidentiality when recruiting, interviewing, and in study transcripts
5. Interviewing and FGD moderation procedures, including audio-taping and note-taking

6. Data quality control, management, and filing procedures
7. Data analysis steps and procedures including participating in the HUS global analysis team to ensure coding consistency across countries

To reflect Ghana's health structure and particular features of Ghanaian community life and social organization, some Ghana-specific approaches will be used in selecting health personnel and community members. These approaches are summarized in Tables A2.1 and A2.2 below.

3.1. Pictorial Diaries to Track Adverse Events

In addition to open-ended interviews with PCGs as described in this protocol, the Ghana team will also ask the caregivers to document any adverse events that occur during the week following immunization in pictorial diaries. Adapted from other prior research in Ghana conducted by a HUS team member [51], these diaries prompt child caregivers to effectively document and communicate what happened to their child and what actions they took in response the events. Caregivers will be asked to indicate adverse events likely to occur or of special interest following RTS,S vaccination based on visual representations of events in the diaries. This method of collecting information facilitates consistent and reliable collection of information from participants, including from non-literate participants. Ghana will adapt diaries used in other research and interventions and pretest the adapted diary prior to using it with the caregivers.

This method for obtaining caregiver reports on adverse events will be shared with other HUS research partners to determine if the method can be adopted by all three countries and to come to specify how it can best be integrated into PCG interviews.

Table A2.1: Summary of Ghana's Data Collection Strategy with Health Personnel

LEVEL	NATIONAL	SUB-NATIONAL	DISTRICT/SUB-DISTRICT	SERVICE DELIVERY
**Group**	Vaccine Implementation Subcommitee Chairs	Regional-level:<br>1. Directors of Health Services<br>2. Community Health Supervisors<br>3. Disease Control Officers<br>4. Health Information Officers<br>5. Malaria Focal Points	Staff on District Health Management Teams (DHTMs) and staff on Sub-District Health Teams	Community Health Nurses who deliver primary health care serivces.
**Selection**	All five chairs will be invited to be interviewed	All five regional staff in each HUS region will be invited to be interviewed	All available team members from DHMTs and from sub-district management teams will be invited to participate in FGDs	DHTMs and Sub-District health teams will be consulted to identify the Community Health Nurses who should be included in the study.
**Method**	One-on-one, in-depth interviews	Short, one-on-one, in-depth interviews	FGDs with the team members	One FGD cohort/community (≤10 individuals/group)
**Purpose/ Topics**	Focus on gaining an understanding of the RTS,S implementation plans, in preparation for Round 1 data collection and cover select, high-level topics listed in Table 9.	Cover topics listed in Table 9 from the various perspectives of individuals filling these five positions, focusing on: regional experiences with RTS,S introduction, delivery strategies and how these change over time and differ across the districts, challenges encountered and actions taken, and impacts on and interactions with EPI and malaria programmes.	Cover topics listed in Table 9 from the various perspectives of individuals serving different functions on these management teams, focusing on district/sub-district level experiences with RTS,S introduction, delivery strategies and how these change over time and differ across the districts, challenges encountered and actions taken, and impacts on and interactions with EPI and malaria programmes.	Cover topics listed in Table 9, focusing provider perspectives on RTS,S, malaria management, and community perceptions. Specific attention will be on any observed changes between the previous immunization sessions and the current one with RTS,S. Challenges in delivering the vaccine and integrating RTS,S delivery with other care will also be explored, as will provider suggestions for improvement.
**Frequency**	Once, prior to Round 1 fieldwork	For each participant, at least once across the three data collection rounds. Additional interviews will be requested with individuals as needed.	FGDs with team members will be held in Rounds 1 and 3	FGDs with the same nurses will be held Rounds 1 and 3
**Language**	English	English	English	English
**Participant Observation**	Ghana research team members will attend the various committee meetings (communication, training and service delivery, data management, logistics and waste management, surveillance and safety monitoring) to understand implementation plans and emergent issues			

Table A2.2: Summary of Ghana's Data Collection Strategy with Community Members

Group	**Community Leaders & Other Opinion-Shapers** (e.g., village elders, chiefs, head teachers, community health volunteers)	**Male Household Heads**	**Female Elders**	**Others** (e.g., traditional healers, pharmacists/drug vendors, local NGO/CBO staff)
**Selection**	The study team will carry out community entry activities which involve seeking the necessary permissions from local authorities and traditional leaders of the communities. These local authorities and traditional leaders will be asked to participate in the study and to suggest other important opinion shapers we may wish to approach and invite to be interviewed.	Male household heads will be identified and invited to attend focus groups (FGs) by community leaders. Community leaders will be asked to consider a mix of characteristics (religion, profession, age) when proposing men for the FGDs.	Same as for male household heads.	Various other individuals will be identified in the course of conducting entry activities, interviews, and participant observation.
**Method**	Short, individual interviews with two different individuals in each community in Rounds 1 and 3	FGDs held in Rounds 1 and 3 including different discussants (≤10 ind/group) in each Round	Same as for male household heads.	Short, individual interviews with two different individuals in each community in Rounds 1 and 3
**Purpose/ Topics**	These short interviews will elicit community leaders' views and perspectives on community acceptance of RTS,S, specifically exploring issues of concern and rumors. They also cover participants' suggestions to enhance RTS,S communications and implementation in their communities.	FGDs with men will elicit their views on malaria burden and management, child health issues and RTS,S, and community perceptions and traditional practices around these broad topics.	Female elders in Ghana have great influence over child care practices in the home as they teach young women how things should be done. In addition topics covered in interviews/FGDs in other groups, FGDs with older women will probe topics around this important social role.	These short interview will focus on topic covered in interviews/FGDs in other groups and will also include questions specific to their social position/role (e.g., local NGO staff may be asked to describe if and how they are participating in community health efforts for RTS,S).
**Frequency**	Each participant will be interviewed one time	Each discussant will participate in only one FGD	Same as for male household heads.	Each participant will be interviewed one time
**Language**	English or local language	Local language	Local language	Local language

Annex 3: Kenya Background

1. MVIP Regions

Kenya is one of seven African countries included in the RTS,S Phase 3 trial. There were three clinical trial sites in Kenya: KEMRI/CDC Research and Public Health Collaboration in Siaya, KEMRI/WRP in Kombewa, and KEMRI/Wellcome Trust in Kilifi. Other ongoing RTS,S clinical trials include the Phase 3 trial in Kombewa and the delayed fractional dose trial in Siaya.

MVIP will be implemented in counties in malaria endemic zones in Western Kenya. Largely rural areas with small towns and a few larger cities, the main economic activities in Western Kenya are farming, fishing and some industry. The predominant ethnic groups are Luhya and Luo. HUS sites will be intentionally selected to approximate ethnic representation in Luhya- and Luo-dominated areas, and to achieve a range of rural-to-urban geographic regions.

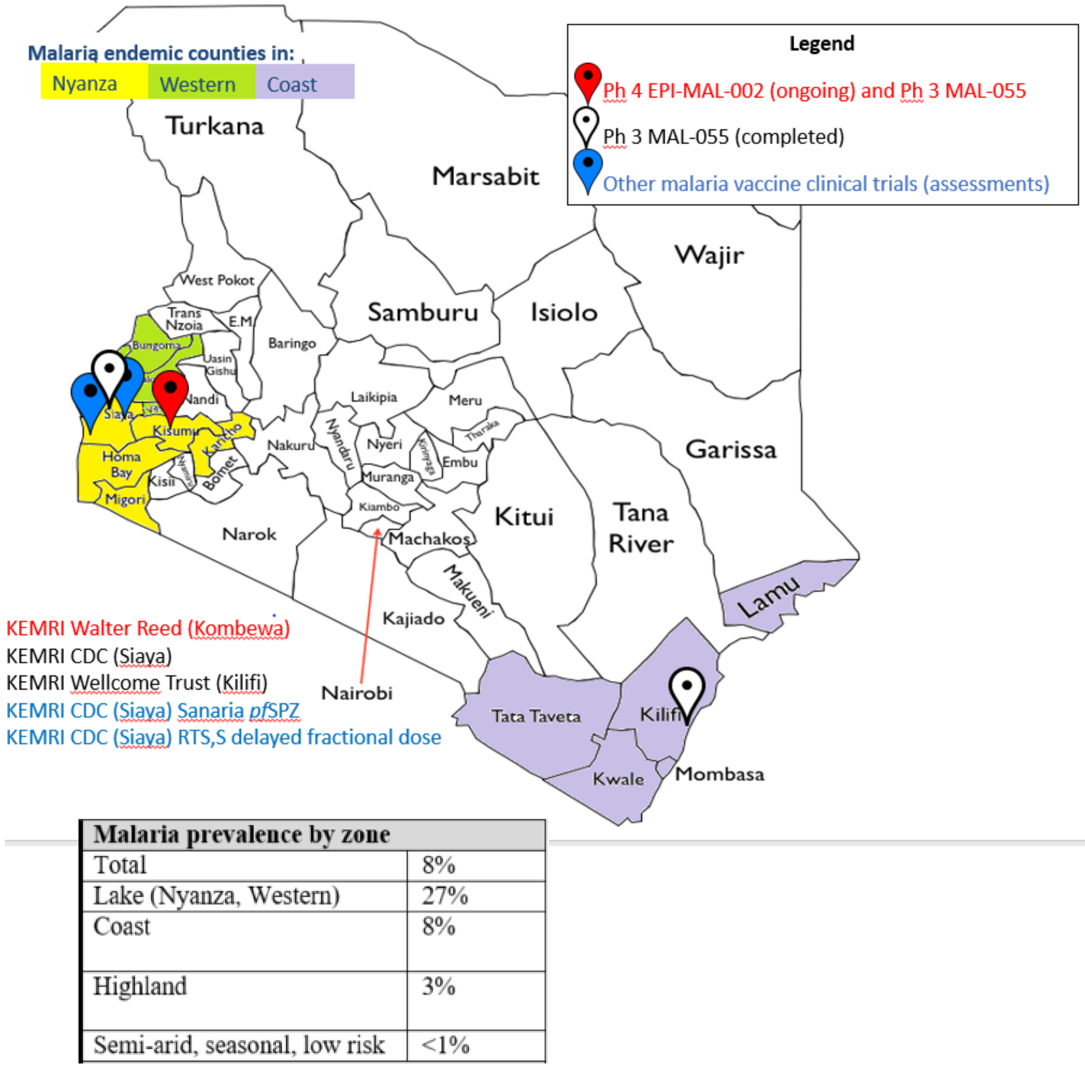

Malaria prevalence by zone	
Total	8%
Lake (Nyanza, Western)	27%
Coast	8%
Highland	3%
Semi-arid, seasonal, low risk	<1%

2. Kenya's Health System[14]

2.1. Service Delivery Structure

Kenya's national health system is organized around two major levels of governance: national and county governments. The National Vaccines and Immunization Programme and the National Malaria Control Programme both fall under the national government. Management of health facilities is largely devolved to county governments based on a multi-tier delivery system:

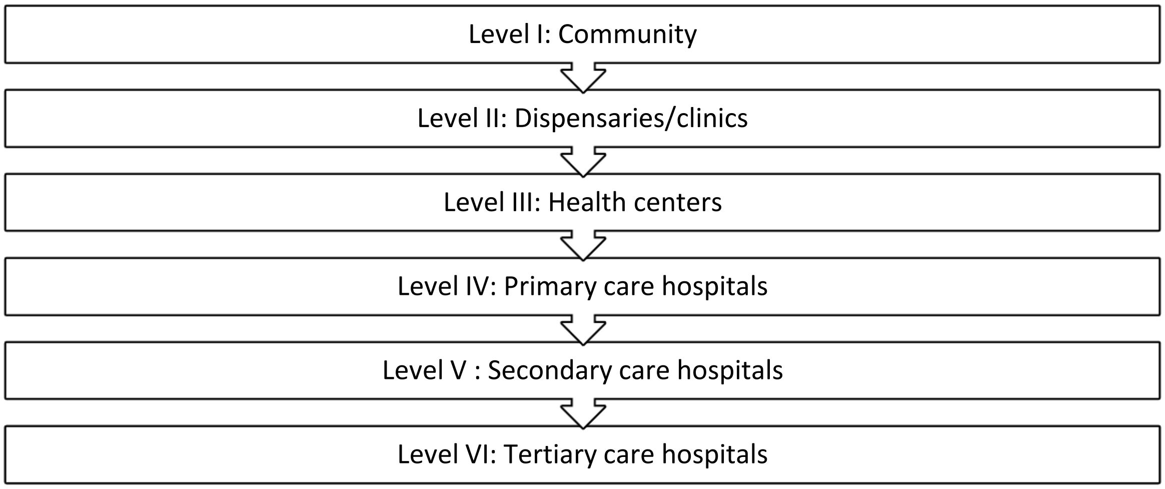

Malaria diagnosis and treatment and immunization services are offered in all health facilities in Kenya. The emphasis is on using the lower health facilities to avoid congestion at the higher-level facilities. However, should a patient/client present to any facility, they will be offered services.

HUS community sites will parallel the health center level, Level III, of Kenya's healthcare delivery system.

2.2. Malaria Burden, Treatment, & Control

National malaria prevalence in Kenya has reduced from 11% to 8%, with the highest reduction reported in the Lake endemic zone where substantial malaria control resources are dedicated and where MVIP will be implemented.

As malaria intervention coverage in Kenya has increased, under-five mortality and infant mortality have declined (see Table A3.1 and Figure A3.1).

[14] Information in this background section comes from the Kenya Country Profile for RTS,S Introduction prepared by PATH (version: March 2017)

Table A3.1: Demographic, Child Mortality, Malaria Case Management Indicators, 2016

**Total population** (2016)	47,251,289	**Malaria case management for children < 5 years**	
**Birth cohort** (2016)	1,586,294	Treatment-seeking	72%
**Surviving infants** to age 1 (2016)	1,508,163	Blood test	39%
**Infant mortality rate** (2015) (deaths <1 year per 1,000 births)	36/1,000	Took ACT	25%
**Child mortality rate** (2015) (deaths <5 years per 1,000 births)	49/1,000	Government facility	70%
**Number of counties**	47	Private facility	25%
**Development status**	Lower middle income (LMIC)	Faith-based health facility	3%
**Gross national income** per capita	USD $1,340	**Long lasting insecticide treated net (LLIN)** usage by children < 5	56%
**GDP** per capita:	USD $29,101		

The rapid reduction in under-five mortality in Kenya from 2003 to 2014 coincided with the rapid scale-up of insecticide-treated net distribution to pregnant women, children under-five, and the general population in the country's malaria risk areas.

Figure A3.1: Malaria Control Coverage Trends and Under-Five Mortality, 2003-2015

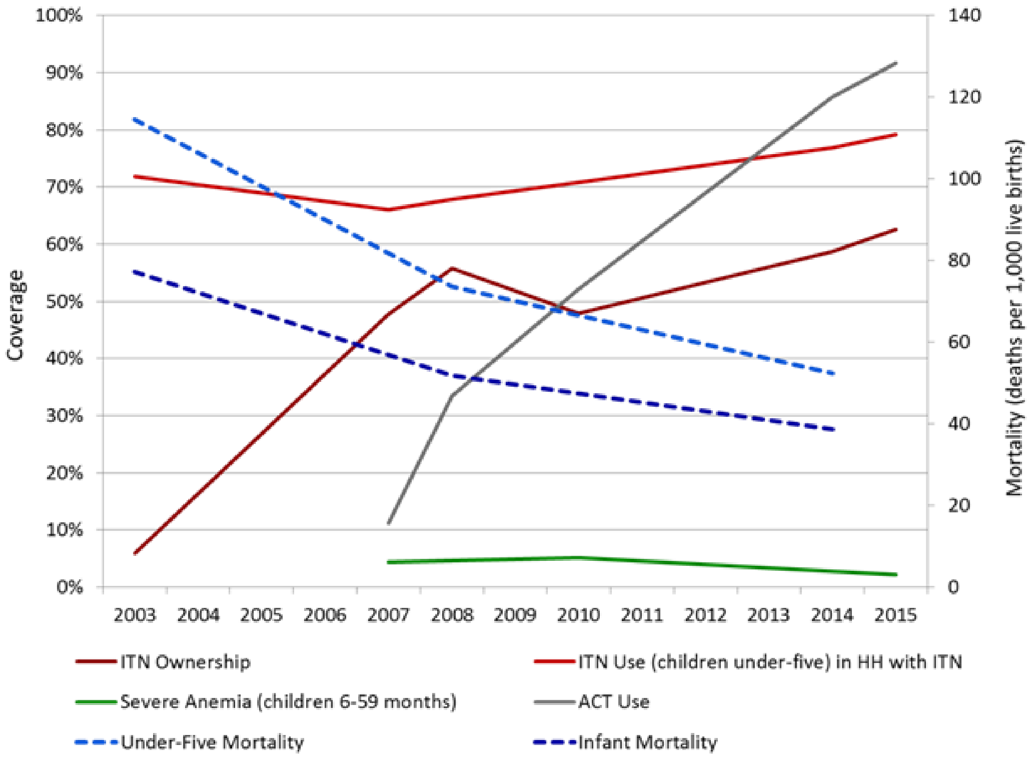

Patients presenting with suspected malaria to any public or select faith-based health facility in Kenya are entitled to free diagnosis and treatment. Health workers are highly encouraged to test all suspected cases, as well as anyone with a history of concurrent fever, using either a malaria Rapid Diagnostic Test or microscopy. Health workers are instructed to treat positive cases with an age-appropriate artemisinin-based combination therapy.

All cases of severe malaria are treated with pre-referral injectable Artesunate and sent to an inpatient facility.

Intermittent Preventive Treatment in pregnancy (IPTp)with Sulphadoxine/Pyrimethamine is prescribed in high transmission areas, and long-lasting insecticide treated nets are distributed to all pregnant women during antenatal care visits. The IPTp policy is at least three doses started immediately and administered one month apart.

2.3. Immunization Services & Coverage

The main strategy for delivery of immunizations shown below is through fixed health facilities, Monday through Friday.

Age	Antigen
Birth	Bacillus Calmette–Guérin (BCG) vaccine<br>Oral polio vaccine (OPV)
6 weeks	Diphtheria, Tetanus, Pertussis & Haemophilus influenza type B & Hepatitis B (DTP-Hib-hepatitis B1)<br>OPV<br>Pneumococcal conjugate vaccine (PCV)<br>Rotavirus vaccine
10 weeks	DTP-Hib-hepatitis B2,<br>OPV<br>PCV<br>Rotavirus vaccine
14 weeks	DTP-Hib-hepatitis B3<br>OPV<br>PCV<br>Rotavirus vaccine<br>Inactivated polio vaccine (IPV)
6 months	Vitamin A
9 months	Measles vaccine<br>Yellow fever vaccine*
18 months	Measles vaccine, second dose
9–11 years (girls)	Human papillomavirus (HPV) demonstration project**

*Yellow fever vaccine only in Elgeyo-Marakwet and Baringo counties in Rift Valley Province.
**HPV demo projects only in Kitui county in Eastern Province.

District health offices may augment fixed point service delivery, however, with outreach services to address the vaccination needs of specially disadvantaged populations. Outreach immunization services can be held on any day of the week, including weekends, to cater to parents, especially mothers, whose only free time may be on weekends. According to Kenya's immunization policy, outreach immunization activities should normally be delivered to the targeted community as part of an integrated package of health services, and all outputs must be documented.

Facility- and outreach-based vaccine delivery in Kenya has led to high coverage rates overall:

Kenya 2015 immunization coverage rates[15]											
BCG	DTP1	DTP3	HEPB3	HIB3	MCV1	MCV2	PAB	PCV3	POL3	ROTAC	YFV
87%	96%	89%	89%	89%	75%	28%	80%	75%	89%	66%	1

Kenya has chosen to administer four doses of RTS,S vaccine using a schedule of 6, 7, 9 and 24 months of age. The minimum interval between all vaccine doses is 1 month.

3. Sampling, Data Collection, Data Management & Ethical Procedures

Overall, the Kenya HUS team will follow the sampling, data collection, and ethical procedures spelled out in this protocol. Specifically, procedures described earlier in this document in relation to the activities listed below will be used in Kenya:

1. The overall sampling strategy to select regions, districts, and community sites
2. The sampling strategy to select households PCGs
3. Interview, FGD, and observation guides included in other annexes
4. Ethics procedures to ensure:
    a. Informed consent
    b. Voluntary participation
    c. Privacy and confidentiality when recruiting, interviewing, and in study transcripts
5. Interviewing and FGD moderation procedures, including audio-taping and note-taking
6. Data quality control, management, and filing procedures
7. Data analysis steps and procedures including participating in the HUS global analysis team to ensure coding consistency across countries

To reflect Kenya's health structure and particular features of Kenyan community life and social organization, some Kenya-specific approaches will be used in selecting health personnel and community members. These approaches are summarized in Tables A3.2 and A3.3 below.

The pictorial diaries to track adverse events proposed by Ghana and described in Annex 2 may be adopted for use in Kenya. This will be determined at a later date.

[15] WHO (2016). WHO vaccine-preventable diseases: monitoring system. 2016 global summary. WHO UNICEF estimates time series for Kenya (KEN) [Online] Available at http://apps.who.int/immunization_monitoring/globalsummary/estimates?c=KEN. Accessed on August 22, 2016

Table A3.2: Summary of Kenya's Data Collection Strategy with Health Personnel

LEVEL	NATIONAL	SUB-NATIONAL	SERVICE DELIVERY
**Group**	National- and county-level malaria, EPI, and MVIP managers	Sub-county Health Management Team (HMT) members, including EPI and malaria program managers	Health workers from facilities located in HUS community sites.
**Selection**	Malaria and EPI programme managers (National and county levels) and the chair of the MVIP TWG (national level) will be invited for and interview by the Kenya PI. Other key stakeholders identified as playing a key role in MVIP during these interviews will also be contacted and invited for interview.	At each data collection round, two members from sub-county HMTs will be selected based on their availability and to reflect the key functions important for vaccine delivery and malaria control services. Sub-county managers selected for interviews will be those with first hand experience in the implementation of the vaccine.	In each district, research staff will consult the district health management team (DHTM) to identify the health workers who should be included in the study. A variety of health workers may be recommended by the DHTM, but in each district we will be sure to include providers working in vaccination and other IMCI services.
**Method**	One-on-one, in-depth interviews	Short, one-on-one or small group interviews	One FGD cohort/community (≤10 individuals/group)
**Purpose/ Topics**	Covering topics listed in Table 9, interviews with national and county health managers will focus on the six health system building blocks: HR, financing, supply chain mgt, governance, and HMIS. Additionally, they will focus on perceptions of the feasibility of integrating RTS,S at the start of delivery (Round 1) and after two years into RTS,S introduction (Round 3).	Covering topics listed in Table 9, these interviews will explore the process, experiences, and challenges of implementing the vaccine in the sub-counties in relation to the six health system building block. Follow up interviews will be conducted with some of the respondents interviewed in the first round of data collection, based on emerging themes during the initial round of data collection.	Provider perceptions of: 1) malaria risk; 2) RTS,S safety and efficacy; 3) the EPI programme and other factors enhancing/constraining implementation of vaccine in general; 4) the MVIP programme implementation strategy; 5) adaptations to their working practices that will be/were required to deliver RTS,S; 6) recommendations to improve delivery of RTS,S in their health facility; and 7) feasibility of implementing RTS,S at scale in the longer term.
**Frequency**	The same individuals, if possible, will be interviewed at Round 1 and Round 3.	For each participant, at least once across the three data collection rounds. Additional interviews will be requested with individuals as indicated by the data.	The same individuals will be asked to participate in discussions during Rounds 1, 2, and 3. As needed, selected staff who move away/into new jobs will be replaced by new staff filling the role.
**Language**	English	English	English
**Participant Observation**	Kenya research team members will attend the various committee meetings (communication, training and service delivery, data management, logistics and waste management, surveillance and safety monitoring) to understand implementation plans and emergent issues		

Table A3.3: Summary of Kenya's Data Collection Strategy with Community Members

Group	**Community Leaders & Other Opinion-Shapers** (e.g., village elders, chiefs, headteachers, community health volunteers, and pastors)	Male Household Heads	Female Elders	Traditional Healers
**Selection**	In each community, various community gatekeepers and opinion shapers will be identified during the ethnographic fieldwork. Identified individuals will be invited for FGDs by field research assistants.	Male household heads will be identified and invited to attend focus groups (FGs) by community leaders. Community leaders will be asked to consider a mix of characteristics (religion, profession, age) when proposing men for the FGDs.	Female elders will be identified with help from community health volunteers. FGDs with female elders is contingent on need as indicated in Rounds 1 and 2 data.	Traditional healers will be identified during the ethnographic phase of the study, and from interviews with primary care givers and other participants who will be interviewed in the first round of data collection. Interviews with traditional healers is contingent on need as indicated in Rounds 1 and 2 data.
**Method**	FGDs held in Rounds 1 and 3 including different discussants (≤8 ind/group) in each FGD	FGDs held in Rounds 1 and 3 including different discussants (≤7 ind/group) in each FGD	FGDs held in Rounds 2 and/or 3 depending on emergent findings (≤8 ind/group)	Short, individual interviews with two different individuals in each community in Rounds 1 and 3
**Purpose/ Topics**	FGDs will elicit community leaders' views and perspectives on community acceptance of RTS,S, specifically exploring issues of concern and rumors. They also cover participants' suggestions to enhance RTS,S communications and implementation in their communities.	FGDs with men will elicit their views on malaria burden and management, child health issues and RTS,S, and community perceptions and traditional practices around these broad topics.	Conducting FGDs with female elders will be determined based on preliminary data analysis. If pursued, FGDs will be designed to cover research questions outlined in the proposal and specifically will address their role in promoting and health decision-making in their households and communities.	Focus will be to understand their knowledge and perceptions of the RTS,S vaccine and programme.
**Frequency**	Each discussant will participate in only one FGD	Each discussant will participate in only one FGD	Each discussant will participant in only one FGD.	Each participant will be interviewed one time
**Language**	English or local language	Local language	Local language	English or local language

Annex 4: Malawi Background

1. MVIP Regions

MVIP sites in Malawi have yet to be determined. The Malawi HUS team remains in contact with MOH and MVIP managers in Malawi and is ready to plan HUS activities based on MVIP site selection.

2. Malawi's Health System[16]

2.1. Service Delivery Structure

The Malawi health service delivery system consists of tertiary, secondary, primary, and community-care levels. District and central hospitals provide secondary and tertiary care services, respectively, but also provide primary care to individuals within their catchment area. At the primary level, services are delivered through community initiatives, health posts, dispensaries, maternities, health centers, and community and rural hospitals. At the community level, health services are provided by community-based cadres, such as Health Surveillance Assistants (HSAs).

Access to health facilities is limited: only half of Malawians live within a five-kilometer radius of a health facility. In response, Malawi has more than 3,500 community health workers in hard-to-reach areas. In addition to other services through village health clinics, community health workers provide a packet integrated health services. HSAs are trained to assess, classify, and provide first-line treatment for selected childhood illnesses, including malaria, in addition to referral to the next level of care. Local community-based organizations also provide non-clinical malaria services such as behavior change communication, counseling, and net distribution.

The Malawi health system is highly decentralized with many programming decisions made at the district level and coordination and supervision done by the zonal level (there are five zones) and the majority of services planned and delivered at the primary care and community levels.

2.2. Malaria Burden, Treatment, & Control

Malaria is the number one cause of morbidity and mortality in Malawi, with an estimated 6 million cases each year representing about 40% of the burden of illness in all health facilities.[17] Across the country, malaria prevalence in children aged 6-59 months tends to be 30% or higher[18] and is the leading cause of morbidity and mortality in children under five years and among pregnant women.[19] According to the Health Management Information System (HMIS) in 2013, it is estimated that Malawi experiences about 4 million episodes of malaria annually.

---

[16] Information in this background section comes from the Malawi Country Profile for RTS,S Introduction prepared by PATH (version: November 9, 2016)
[17] Malawi Malaria Strategic Plan 2011-2015
[18] Indepth Network 2015
[19] Malawi Malaria Indicator Survey 2014

Malaria is endemic in more than 95% of the country. Transmission is perennial in most parts of the country and peaks after the start of the annual rains, which typically begin in November and last through April.[5]

Malaria is one of the main public health priorities within the country's Essential Health Package. Over the past five years, Malawi has made notable progress in malaria control through vector management (LLIN distribution and indoor residual spraying), diagnosis-based case management, and preventive treatment in pregnant women.

2.3. Immunization Services & Coverage

Malawi's EPI program has performed well since its establishment in 1979. In 1989, ten years later, the country attained the universal immunization goal when coverage was 80% and above for all antigens.

**Current vaccines**	**Coverage**	**Schedule**	**Strategy** (routine/campaign/mixed)
DTP3 coverage	89%	% of districts reporting DTP3 >80%	89%
Measles	88%	9 months	Routine
Rota2	81%	6,10 weeks	Routine
Vitamin A[9]	90%	At 6 months and every 6 months up to 59 months	Routine
PCV3<br>HPV1 pilot	89%<br>95%	6,10,14 weeks<br>2 doses	Routine<br>Pilot in Rumphi and Zomba districts
**Future planned vaccines**	**Year**	**Type of introduction**	**Strategy** (routine/campaign/mixed)
IPV	2016	At 14 weeks with OPV 3, PCV3 and DPT-HepB-Hib-3.	EPI routine
MCV2	2016	Grade 4 and 10 year-olds	EPI routine
HPV	2017	Girls at 0 and 6 months	TBD
Rubella-containing measles vaccine	2017	TBD	TBD

The high immunization coverage has since been sustained, except in the occurrence of a global vaccine shortage or a change in the recommended statistical proportion of children under one

year of age,[20] and the number of districts with more than 80% DPT-HepBHib3 coverage has been steadily increasing for the past five years.[21]

Malawi has chosen to administer four doses of RTS,S vaccine using a schedule of 5, 6, 7 and 22 months of age. The minimum interval between all vaccine doses is 1 month.

3. Sampling, Data Collection, Data Management & Ethical Procedures

Overall, the Malawi HUS team will follow the sampling, data collection, and ethical procedures spelled out in this protocol. Specifically, procedures described earlier in this document in relation to the activities listed below will be used in Malawi:

1. The overall sampling strategy to select regions, districts, and community sites
2. The sampling strategy to select households and PCGs
3. Interview, FGD, and observation guides included in other annexes
4. Ethics procedures to ensure:
    a. Informed consent
    b. Voluntary participation
    c. Privacy and confidentiality when recruiting, interviewing, and in study transcripts
5. Interviewing and FGD moderation procedures, including audio-taping and note-taking
6. Data quality control, management, and filing procedures
7. Data analysis steps and procedures including participating in the HUS global analysis team to ensure coding consistency across countries

To reflect Malawi's health structure and particular features of Malawian community life and social organization, some Ghana-specific approaches will be used in selecting health personnel and community members. These approaches are summarized in Tables A4.1 and A4.2 below.

The pictorial diaries to track adverse events proposed by Ghana and described in Annex 2 may be adopted for use in Kenya. This will be determined at a later date.

---

[20] Malawi Comprehensive Multi-Year Plan 2012-2016

[21] World Health Organization (WHO). WHO/IVB database, data reported to WHO by member states and WHO-UNICEF estimates of immunization coverage (WUENIC).

Table A4.1: Summary of Malawi's Data Collection Strategy with Health Personnel

LEVEL	NATIONAL	SUB-NATIONAL	SERVICE DELIVERY
**Group**	National Malaria Programme Director, EPI Programme Coordinator, and President's Malaria Initiative (major malaria funder) program manager	District-level staff, including:<br>1. EPI program coordinators<br>2. Malaria Programme Coord.<br>3. Environmental Health Coord.<br>4. District Health Managers	HSAs, nurses in under five clinics, environmental officers, and health center in-charge
**Selection**	All individuals holding these positions will be invited to an interview.	All individuals in each district included in the study will be invited to an interview.	The sample will be drawn from health centres responsible for healthcare provision in the selected communities (i.e., saff that manage health posts in the 9 villages).
**Method**	One-on-one, in-depth interviews	One-on-one or small groups	One FGD cohort/community (≤12 individuals/group)
**Purpose/ Topics**	Interviews will cover topics listed in Table 9, focusing on perceptions of the feasibility of integrating RTS,S at the start of delivery (Round 1) and after two years into RTS,S introduction (Round 3).	Covering topics listed in Table 9, these interviews will explore the process, experiences, and challenges of implementing the vaccine from the district perspective in relation to the six health system building block.	Covering topics listed in Table 10, discussion focus on experiences of RTS,S introduction, delivery strategies and how these have differed within the catchment area or over time, challenges encountered and action in response, impacts on other health programmes, including EPI and malaria control, and perceptions of community response.
**Frequency**	The same individuals, if possible, will be interviewed at Round 1 and Round 3.	The same individuals, if possible, will be interviewed at Round 1 and Round 3.	The same individuals will be asked to participate in discussions during Rounds 1 and 3.
**Language**	English	English	English
**Participant Observation**	Malawi research team members will attend the various committee meetings (communication, training and service delivery, data management, logistics and waste management, surveillance and safety monitoring) to understand implementation plans and emergent issues		

Table A4.2: Summary of Malawi's Data Collection Strategy with Community Members

Group	**Community Leaders & Other Opinion-Shapers** (e.g., village elders, chiefs, headteachers, community health volunteers, and pastors)	**Male Household Heads**	**Female Elders**	**Other** (e.g., traditional healers, pharmacists/drug vendors, local NGO/CBO staff)
**Selection**	In each community, various community gatekeepers and opinion shapers will be identified during the ethnographic fieldwork. Identified individuals will be invited for FGDs by field research assistants.	Participants will be identified through discussion with local research representatives where available, or health workers and other community contacts identified as part of the ethnographic observation. Individuals will be selected to represent a range of ages and other demographic criteria.	Same as for male household heads but selecting for women who have grandchildren.	Other health service providers including traditional healers, drug vendors, and local NGO staff will be identified through ethnographic engagement.
**Method**	One-on-one, in-depth interviews	FGDs held in Rounds 1 and 3 including different discussants (≤12 ind/group) in each FGD	FGDs held in Rounds 1 and 3 including different discussants (≤12 ind/group) in each FGD	One-on-one, in-depth interviews
**Purpose/ Topics**	Topics for these interviews will include community norms and behaviour regarding malaria and vaccination, perspectives on the RTS,S implementation, and formal and informal service provision for malaria, vaccination and other childhood illnesses.	FGDs will consider social and cultural norms, particularly regarding malaria and vaccinations, experiences with RTS,S, and contextual histories that may influence perceptions, (e.g., community experience of past vaccination, malaria or other health campaigns).	FGDs will consider social and cultural norms, particularly regarding malaria and vaccinations, experiences with RTS,S, and contextual histories that may influence perceptions, (e.g., community experience of past vaccination, malaria or other health campaigns).	Topics will include services and advice provided in cases of suspected malaria, advice regarding vaccination, and experiences of community health seeking behaviour, including changes over time.
**Frequency**	The same individuals will be interviewed in Rounds 1 and 3	Each discussant will participate in only one FGD	Each discussant will participant in only one FGD.	The same individuals will be interviewed in Rounds 1 and 3
**Language**	English or local language	Local language	Local language	English or local language

Annex 5: Participant Observation Guide

This document is intended to guide HUS research assistants involved in ethnographic fieldwork. It represents only a general framework for conducting observations, participating in community events, and writing up fieldnotes. Decisions about what events to attend, when and where to conduct observations, and specific issues to explore in depth should be determined based on local circumstances.

Events and Issues of Interest

1. How does the general public and caregivers of vaccine-age children learn about RTS,S?:
   - in what context?
   - what is said?
   - what is heard?
   - what questions are asked?
   - how are these questions answered?

   Where to observe: health education campaigns, peer-to-peer education sessions, group education sessions, health communication planning meetings

2. How do health providers learn about RTS,S?:
   - which providers?
   - in what context?
   - what is said?
   - what is heard?
   - what questions are asked?
   - are these questions answered?

   Where to observe: provider trainings and RTS,S orientation sessions (if possible)

3. What questions and concerns about RTS,S in the general population and among mothers of vaccine-age children persist?:
   - what are people saying about the vaccine?
   - what sources of 'evidence' are provided to support popular claims?
   - how do people engage with the professional medical sector for clarity?
   - how is RTS,S represented in local media (radio, newspaper)?

   Where to observe: vaccination clinic waiting rooms, promotion/health education events focused on RTS,S, and everyday spaces (markets, taxi-buses, etc.)

4. Specific issues of interest:
   - how are messages on RTS,S partial protection framed, popularly understood, and (re)interpreted by the general public?
   - what are people saying/understanding about the need for continued malaria prevention behaviors with RTS,S?
   - what are people saying/understanding about the need for additional vaccination visits for RTS,S?

Where to observe: vaccination clinic waiting rooms, promotion/health education events focused on RTS,S, everyday spaces (markets, taxi-buses, etc.)

Participant Observation

1. *Participate*: Freely engage in conversations and question-and-answer sessions as a fellow community member.

   Probe people's ideas, thought processes, and rationales. Do not let your notetaking interfere with your engagement with people you are talking to or the meeting you are attending. Allow yourself to fully participate and "be there."

2. *Observe*: Sometimes it is best simply to observe discussions and interactions. Use your best judgement to decide when you need to remain a distant observer or a participant in the dynamic. While capturing the broader context of the interactions you are observing, try to focus on a few key issues/topics that are especially pertinent to the HUS.

3. *Record*: It is often entirely appropriate to take notes openly as you observe and participate in an exchange. In these instances, be transparent about what you are doing and let people know that you are part of a team to document community responses to RTS,S introduction.

   If you cannot take notes during an interaction, try to jot down essentials to jar your memory for later write-up. Type up your observations as soon after the observation as possible.

4. *Ethics*: A core principle of ethical research with human subjects is that individuals are free to choose or to decline to participate in a study. In that ethnographic fieldwork relies on informal, naturalistic and often spontaneous interactions with people in everyday situations, the essential quality of ethnographic fieldwork runs contrary to the ethical principle of voluntary participation. In the HUS we address the issue through multiple public announcements about the ongoing study. To allow individuals to choose if they want to engage or not with the field worker it is important that they remain transparent about the fact that they are involved in a research activity and specifically that they are: (i) informally observing and interviewing people in the community to learn about their views on child health, malaria, and the new RTS,S vaccine and (ii) taking notes on the different viewpoints they hear, excluding any information about individuals or groups that would identify them.

Fieldnotes Suggestions

1. *Daily upkeep*: Type up your notes daily using a template to prompt you to capture key information about context, date, time, who was involved, and other important details about the exchange observed.

2. *Observation entries*: Use an entry system to distinguish between different events, incidents, conversations, and exchanges observed – each unique observation should be assigned an entry identifier and include contextual information.

3. *Focus on description*: In your notetaking, try to focus on simply describing what you have observed and participated in. Refrain from premature interpretation and making judgements. For instance:

   - *Descriptive*: A woman who appeared to be in her 30's asked a question about why she needed to keep using bed nets with her child if she takes her for all four doses; the health educator replied: "Because the vaccine helps to make malaria less severe, but it doesn't stop it from occurring." **The woman turned to her friend, raised an eyebrow as if she still had questions.**

   - *Judgmental*: A woman who appeared to be in her 30's asked a question about why she needed to keep using bed nets with her child if she takes her for all four doses; the health educator replied: "Because the vaccine helps to make malaria less severe, but it doesn't stop it from occurring." **The woman was unsatisfied with this response and clearly skeptical about getting her child vaccinated.**

Annex 6: Primary Caregiver Profile Sheet

Date data entered: ____________________ Date data verified: ____________________

Entered by: ____________________ Verified by: ____________________

HUS Primary Caregiver Profile Sheet	*Version: 16 August 2018*

Sample	1 = Cohort, R1	2 = Cohort, R2	3 = Cohort, R3	4 = Cross-sectional

Profile Sheet: Background & Socio-demographics

**Interviewer**		**Interview date**	____ Day ____ Month ____ Year
**Interview language**			

**Country**	1 = Ghana	2 = Kenya	3 = Malawi
**Community**	1 = Community name<br>2 = Community name<br>3 = Community name	4 = Community name<br>5 = Community name<br>6 = Community name	7 = Community name<br>8 = Community name<br>9 = Community name

**PCG ID#**	____ Country	____ Community	____ Group
			____ ____ ____ Individual
**Relation to RTS,S-eligible child**	1 = Mother<br>2 = Father	3 = Grandmother<br>4 = Sister/brother	5 = Aunt/Uncle<br>6 = Other

**RTS,S-eligible child**			
**Child's Name**		**Birth Date**	____ Day ____ Month ____ Year
**Child's sex**	1 = Male	2 = Female	

CAREGIVER & HOUSEHOLD PROFILE

**PCG's sex**	1 = Male	2 = Female	**Age (years)**		**Education (# years completed)**	

**Marital status**	1 = Currently married	2 = Widowed, divorced, separated	3 = Never married

**What is your principal income-earning occupation?**	**Some HH SES indicator (TBD)**
1 = formal employment	
2 = subsistence farming	
3 = small commerce	
4 = other	
5 = none	

**How many years have you lived in this community?**	________	

**Religion**	

**In addition to [RTS,S eligible child], how many other children do you have?**		**Of these other children, how many are under the age of 5 years old?**	

**Are you the head of the household?**	1 = yes<br>0 = no	

**What is your closest health center/clinic/post?**	
**How long does it take to get to [name of facility] from here?**	
**How do you typically get there?**	

Annex 7: Primary Caregiver Interview Guides

Section A: Warm Up

Thank you for your replies. I want to start the interview by asking you a couple of questions about children's health in your family and your community.

A1 Perceived Child Health Threats

A1.1 What would you say is the most serious health threat in your community for young children, children five years old or younger?

Probe: Why do you say this?
Has your child / any of your children experienced this?

A1.2 What is another very serious health threat in your community for young children?

Probe: Why do you say this?
Has your child / any of your children experienced this?

A1.3 Can you tell me about one more very serious health threat for young children that you would include?

Probe: Why do you say this?
Has your child / any of your children experienced this?

A2 General Perceptions about Child Health Services & Vaccines

A2.1 Thinking about your children's health, list for me all the reasons why you take your child/ren to the clinic/health center.

From all the reasons you just listed, what's the most important reason you take your child to the clinic/health center? And why is that?

A2.2 How do you learn about child health services?

Probe:

□ From who? / What context?

□ Who do you trust most to learn about health services for children? Why?

□ Can you describe to me a recent example of when you learned about a service available at your health facility?

A2.3 *If caregiver mentioned vaccines, ask*: I'd like you to tell me more about your views on child vaccinations. *Pause, then probe*: How would you describe the purpose of vaccinations for children?

If caregiver did <u>not</u> mention vaccines yet, ask: Tell me about your views on child vaccinations. *Pause, then probe*: How would you describe the purpose of vaccinations for children?

A2.4 When there's a new vaccine available, how do you learn about it? Can you give me a specific example?

A2.5 What are your main concerns about your child / ren getting a new vaccine?

Section B: Vaccination Service Experience

Thank you very much for sharing those thoughts with me. Now I'd like to hear about your experiences taking your child/ren for vaccination.

B1 Vaccination Uptake

B1.1 Since [RTS,S-eligible child] was born, have you taken her/him for vaccination?

<table>
<tr><td>1 = yes</td><td colspan="2">0 = no</td></tr>
<tr><td rowspan="2">Go to question B2.1 in the first (unshaded) column below</td><td colspan="2">B.1.2 What about another one of you children, have you taken one of them for vaccination?</td></tr>
<tr><td>1 = yes → Ask:
B.1.3 About when was the last time you took a child for vaccination?
B.1.4 How old was the child?
Go to question B2.1 in the first (unshaded) column below</td><td>0 = no → Go to question B2.1 in the second (shaded) column below</td></tr>
</table>

B2 Vaccination Visit Experience

**Has taken a child for vaccination**	**Has never taken a child for vaccination**
Thank you. I now want to ask you some questions about your most recent vaccination visit with [RTS,S-eligible child / your child]. Please take a moment to think about that visit.<br><br>B2.1 Please tell me about that experience beginning to end.<br><br>*Allow the caregiver to describe the experience fully and check off each topic listed below as s/he describes it. Then probe for information on topics not discussed or where more detail is needed/useful.*<br><br>□ *Service availability*: What do you feel about the convenience and availability of services?	No problem. I'd now like to ask you some questions about what you've heard from others about vaccination services. Please take a moment to think about things you've heard.<br><br>B2.1 Please tell me about all the things you've heard about vaccination visits. Describe anything you can think of.

Has taken a child for vaccination	Has **never** taken a child for vaccination
□ *Staff attitudes and behavior*: How would you describe staff attitudes. For example, the welcome you received and staff politeness.<br><br>□ *Physical environment*: What are your thoughts on the physical conditions of the service, for example, cleanliness and comfort?<br><br>□ *Provider competence*: What are your impressions about the providers' knowledge and skills? Why do you say this?	
B.2.2 Can you please tell me about health information your received from the providers during your visit.<br><br>*Allow the caregiver to describe information received fully and check off each topic listed below as s/he describes it. Then probe for information on topics not discussed or where more detail is needed/useful.*<br><br>□ *Vaccine benefits*: What did the providers tell you about the purpose of vaccinations for children?<br><br>□ *Schedule/return visit*: What did they say about bringing the child back? Please be specific.<br><br>B2.2.1 Did you have any questions about the vaccine that didn't get answered/addressed by the providers? What were they?<br><br>B2.2.2 Did the providers give you information about other child health issues? Which ones? Please describe them.	B2.2 SKIP
B2.3 Overall, how do you feel about the quality of care you and your child received during this visit? Why do you feel this way? Please be specific.	B.2.3 Has anything you've heard about vaccination services discouraged you

Has taken a child for vaccination	Has <u>never</u> taken a child for vaccination
B2.4 If you had to pick just one thing that would improve clients' experiences using vaccination services, what would that be? Why did you pick that?	from taking your own child for vaccination? Please tell me more?<br><br>B2.4 If you had to pick just one thing that would encourage you to use vaccination services, what would that be? Why did you pick that?

Section C: Child Health/Vaccination Decision-Making

Thank you for sharing your impressions with me. I'm going stay focused on vaccination for a few more questions.

C1 Child Health/Vaccination Decision-Making & Preparation

**Participant described a vaccination service experience.**	**Participant did not describe a vaccination service experience.**
C1.1 In relation to your recent vaccination visit that you just described, please tell me how it was decided to take the child for vaccination.<br><br>*Allow the caregiver to describe the decision-making process fully and check off each topic listed below as s/he describes it. Then probe for information on topics not discussed or where more detail is needed/useful.*<br><br>□ Who was involved in making the decision? How specifically?<br><br>□ Who made the final decision?<br><br>□ What issues were considered in the process? Why were these things considered?	C1.1 In your household, how is it decided to take a child for a health service?<br><br>*Allow the participant to describe the decision-making process fully then probe:*<br><br>Who is involved in making these decisions? How specifically?<br><br>What issues are considered in the process? Why were these things considered?
C1.2 Once the decision was made to take the child for vaccination, please tell me about how you prepared for the visit.<br><br>*Allow the caregiver to describe the preparation process fully and check off each topic listed below as s/he describes it. Then probe for information on topics not discussed or where more detail is needed/useful.*<br><br>□ What did you do to remember the date?	C1.2 SKIP

□ How did you manage the visit with work and other duties?<br><br>□ What did you have to do to get to the service? Did anyone help you get there? Please explain.<br><br>C1.3 Are there times when you've decided not to go, or you can't go, for vaccination?<br><br>1 = yes → Please tell me about such an instance. *Probe*: Why? What happened? Who was involved?<br><br>0 = no	C1.3 Can you please tell me the reasons that [RTS,S-eligible child] has not been vaccinated according to schedule.<br><br>What are your plans for taking her/him for vaccination in the future?

Section D: RTS,S Exposure

D1 RTS,S Exposure

D1.1 Have you heard about the new malaria vaccine called [RTS,S]?

1 = yes (have heard of RTS,S)	0 = no (have not heard of RTS,S)
D1.2 Where did you hear about [RTS,S]/what context?	D1.2 SKIP
D1.3 What have you heard specifically?<br><br>*Probe*: Who told you this? Where did you hear this?<br><br>What else have you heard about [RTS,S]? What are people in the community saying about [RTS,S]?	D.1.3 SKIP
D1.4 What questions do you, or other people in the community, have about the new malaria vaccine?	D1.4 SKIP
D1.5 What is the best way for people in the community to learn about this new vaccine?	D1.5 What is the best way for people in the community to learn about new child health services, including vaccines.
D1.6 Do you plan to take [RTS,S-eligible child] for their next scheduled [RTS,S] vaccination?<br><br>Why? / Why not?	D1.6 Would you be interested in a vaccine that could protect your child from malaria?<br><br>Why? / Why not?

Section E: Vaccination History & Perceived Adverse Events

Thank you for all the information you've shared with me so far.

E1 Vaccination History

Participant described a vaccination service experience.	*Participant did not describe a vaccination service experience.*
↓	↓
E1.1 I'd like to see [RTS,S-eligible child]'s card to write down some information.<br><br>*Complete the vaccination history table on the next page and ask all questions under 5.2.*<br><br>***Be sure to fill in participant ID number, interview date, and child's vaccination card number om the table.***	E1.1 Skip or: Do you plan on taking [RTS,S-eligible] child for vaccination? When?<br><br>*Go to Section 6.*

PCG ID#	_____ Country	_____ Community	______ Group	_____ _____ _____ Individual

Interview Date	______ Day	______ Month	______ Year

Card #:	

VACCINATION HISTORY

Card #:	Routine Doses Received	Date Doses Received	RTS,S dose given?	AEFI observed?
Birth		______ Day ______ Mo ______ Year		1 = yes 0 = no
Visit 1		______ Day ______ Mo ______ Year	1 = yes 0 = no	1 = yes 0 = no
Visit 2		______ Day ______ Mo ______ Year	1 = yes 0 = no	1 = yes 0 = no
Visit 3		______ Day ______ Mo ______ Year	1 = yes 0 = no	1 = yes 0 = no
Visit 4		______ Day ______ Mo ______ Year	1 = yes 0 = no	1 = yes 0 = no
Visit 5		______ Day ______ Mo ______ Year	1 = yes 0 = no	1 = yes 0 = no
Visit 6		______ Day ______ Mo ______ Year	1 = yes 0 = no	1 = yes 0 = no
Visit 7		______ Day ______ Mo ______ Year	1 = yes 0 = no	1 = yes 0 = no
Visit 8		______ Day ______ Mo ______ Year	1 = yes 0 = no	1 = yes 0 = no
Visit 9		______ Day ______ Mo ______ Year	1 = yes 0 = no	1 = yes 0 = no

E2 Perceived Benefits & Adverse Events

For each vaccination visit made ask all the questions under E2.1 below. To make sure that you cover each vaccination visit, in the table above put a check mark next to the visit once you finish all questions under E2.1.

Visit by visit, I'd like you to please tell me about both health benefits and adverse effects that [RTS,S-eligible child] had after getting vaccinated.

Let's start with the vaccinations s/he received at birth. / Okay, now let's talk about the [*Nth* visit].

E2.1 What did you learn about health benefits for [RTS,S-eligible child] from vaccinations received [at birth / during visit X]?

E2.2 Did you observe health issues (adverse events) caused by the birth vaccines in [RTS,S-eligible child]?

1 = yes	0 = no
E2.2.1 Please describe these to me.<br><br>E2.2.2 Why do you think these events were caused by the vaccinations?<br><br>Did the health provider tell you anything about possible effects from the vaccination? What did s/he tell you?<br><br>E2.2.3 What did you do in response to the event?<br><br>Why did you take these steps?<br><br>E2.2.4 How do side effects like the ones you've just describe influence your decision to vaccinate your child again?<br><br>How so? / Why not?	*Repeat questions E2.1 and E2.2 for the next vaccination visit made. When all visits have been covered, go to Section F.*

Section F: Malaria Perceptions & Behavior

I'm now going to switch topics from vaccination to malaria. I'd like to understand more about malaria in your community and household.

F1 Malaria Perceptions

F1.1 How would you describe the importance of malaria in your community?

Probe: Why do you say this?
Who in your community is most affected by malaria? How so? Why do you think this is?

F1.2 What about in your household/family? How would you describe the importance of malaria?

Probe: Why do you say this?
Who in your family is most affected by malaria? How so? Why do you think this is?

F2 Malaria Treatment Seeking

F2.1 Can you please tell me if [RTS,S-eligible child] has ever had malaria?

0 = no → That's good news. I'd like you to think about another one of your children (or close relative who is a young child) who recently had malaria. Take a minute to remember the most recent incident you can recall. *Give the caregiver a moment to think then ask question F2.2.*

1 = yes → I'm sorry to hear that. I want to learn more about her/his malaria episode, so please take a moment to remember it. *Give the caregiver a moment to think then ask question F2.2.*

F2.2 Please tell me about what happened, beginning to end.

Allow the participant to describe the episode fully and check off each topic listed below as s/he describes it. Then probe for information on topics not discussed or where more detail is needed/useful.

□ *Symptoms recognition*: What specifically made you first think there was a problem with your child/the child?

How old was the child?

□ *Malaria diagnosis*: How did you know / find out that your child/the child had malaria?

□ *Illness progression*: How did her/his symptoms change over time?

□ *Perceived severity*: How serious was the malaria episode in your child? Why do you say this? Please be specific.

F3 Malaria Treatment Seeking

F3.1 What steps were taken to help the child? Include all the things you tried at home, the clinic or elsewhere, starting with the very first thing you tried and then describe everything you did after that up to the last thing you did.

Allow the caregiver to describe the whole care-seeking course, jotting down each treatment action taken cited in the Treatment Action rows in the table below. Try to jot them down in the order they are cited.

**Treatment Actions**	
1	
2	
3	
4	
5	
6	

Thank you for this information. I'm going to read back to you all the things you said you did to treat your child's malaria.

Read the list of actions that you jotted down then ask: Did you do anything else?

Jot down any additional actions. Then, <u>for each treatment action</u> jotted down ask all questions under F3.2. To make sure that you cover each treatment action, put a checkmark next to it in the table above once you finish all questions under F3.2.

Now I'm going to ask you a few questions about each action you told me about.

F3.2 Let's start with the first thing you did. / Okay, now let's talk about [Treatment Action X].

□ Why did you do / take [Treatment Action X]?

□ Who was involved in [Treatment Action X]? How were they involved specifically?

□ What things did you have to purchase or pay for in order to do / take [Treatment Action X]? Think about all the costs, including transportation, drugs, consultation fees, and so on. You don't have to give me values, just list these things you had to pay for.

F3.3 At any time during the illness, did you lose wages/earnings in order to treat your child? Please tell me more about this.

F3.4 At any time during the illness, did you have to use savings, borrow money, or sell anything in order to treat your child? Please tell me more about this.

F3.5 How did your child get better from malaria?

F3.6 Approximately when did your child get better? *Try to get an approximate date.*

F4 Malaria Prevention Beliefs & Behaviors

F4.1 Thinking about the malaria episode you just described, how do you think it could have been prevented? Why? / How so?

How could you protect your child in the future?

F4.2 In your household, what do you do specially to protect young children from getting malaria? Please elaborate. Why do you do these things?

F4.3 Do you have bed nets in your household?

0 = no → Why not?.

1 = yes → Did [RTS,S-eligible child] sleep under a bed net last night?

We are now done with the interview. Thank you very much for all the information you've given me and for taking the time to share your experiences and views. Do you have any questions for me at this time?

Annex 8: Health Provider Profile Sheet and FGD Guide

**HUS Health Provider Profile Sheet & FGD Guide Round 1**	***Version: 16 August 2018***

**Provider Profile Sheet**

**FGD moderator**		**FGD date**	______ Day ______ Month ______ Year

**Country**	1 = Ghana	2 = Kenya	3 = Malawi
**Community**	1 = Community name<br>2 = Community name<br>3 = Community name	4 = Community name<br>5 = Community name<br>6 = Community name	7 = Community name<br>8 = Community name<br>9 = Community name

**FGD ID#**	____ Country	____ Community	____ Group	____ ____ ____ Group

**About the Participant's Facility**							
**Facility Name**							
**Facility Type**	1 = primary health center						
	2 = community health post						
	3 = other:____						
**Number of Staff (assigned to EPI)**							
**Vaccination Services**	*Circle all days that apply*						
Days offered at facility:	Mon	Tue	Wed	Thu	Fri	Sat	Sun
Outreach services offered:	1 = yes		2 = no				

**About the Participant**			
**Highest Degree Earned**			
**# of years in practice**			
**# years at present facility**			
**# years delivery vaccines**			
**Most recent EPI training**	Approximate date:_________________________________		
**Received RTS,S training**	1 = yes	2 = no	Date:______________

Section A: Warm Up (≈10 min)

Welcome, review of ethics and of the FGD's purpose, topics and process.

A1 Vaccination Benefits & Challenges

A1.1 I want to start off the discussion with a broad topic: the role of vaccinations in primary healthcare in COUNTRY. Can one of you please get us started by describing your understanding of the role of vaccinations in COUNTRY.

Who'd like to add to that comment/view? Anyone else?

A1.2 Clearly, there are a lot of benefits for community health from vaccinations, but what about the challenges of providing these services? From the perspective of the role you hold in the clinic, how would you describe the main challenges today in getting children in your community fully vaccinated?

Do others share this view? Can someone please elaborate? What other challenges can you tell me about?

A1.3 Now let's talk about a vaccination for malaria. How do you feel about now having a vaccine that helps prevent malaria?

From what you've learned about RTS,S, what do you think about its potential to help combat malaria in COUNTRY.

Please elaborate. Who wants to add to that?

Section B: Feedback on the RTS,S Launch (≈30-40 min)

B1 RTS,S Launch – fully open feedback

B1.1 Your facility recently started providing RTS,S to children. Let's talk about how the RTS,S introduction went. So, how did it go? What else? Anyone else?

Because its so important to learn from the actual experiences of providers giving the services, I want us to spend a little more time on this to make sure that we cover everything your can think of that went right as well as what needs to be improved.

B2 Provider Training

B2.1 Let's start from the beginning and how providers and the clinic prepared to start giving the vaccine. Tell me about the training your received?

- What major take-aways did you learn in the training?
- What questions did you still have about the vaccine and giving it to children? Do you still have these questions?
- After being trained, did you feel well prepared to start providing the vaccine? How so? / Why not?
- What could be improved in the training that would help providers feel more prepared to deliver RTS,S?

B3 Facility Preparations

B3.1 What steps were taken at the facility to get ready to provide RTS,S?

- *Probe*: New scheduling/additional client visits, supplies, job aides, client education materials.
- How did you learn about what was going on to introduce RTS,S?
- Did you feel adequately informed about what was happening and how the new vaccine would affect your work? Please elaborate.
- When it came to start providing the vaccine, was the clinic ready? How so? / Why not?
- Based on your experience, what would you say could be improved for health teams/clinics to be ready to deliver the new RTS,S vaccine?

B4 Delivery Experiences

B4.1 Now please tell me a bit more about your experiences actually delivering RTS,S, focusing on how well it's delivery was integrated into existing routines at the facility.

- How would you describe how well messaging about RTS,S was integrated with existing client education sessions?
  - What is needed to improve this?
- What about giving the injection itself, tell me about how smoothly – or not – RTS, S provision was integrated into your existing vaccination routines.
  - What can be done to improve this?
- Now let's talk about record keeping. Tell me about the added task of keeping track of RTS,S vaccinations provided.
  - What can be done to improve this?

B5 Four-dose Schedule Concerns

B5.1 When you think about the 4-dose RTS,S schedule, what are your major concerns about providing these? Please elaborate.

- What do you think would help ensure the greatest success in delivering all four doses to children?

Section C: Community & Client Response (≈15 min)

This is a good time to transition to a discussion about your clients' and the community's response to RTS,S.

C1 Client & Community Response

C1.1 Do you think the clients were ready to receive RTS,S? By this I mean, had they heard about it, were they interested in it, were people curious and did they have questions?

What gives you that impression?

C1.2 What kinds of questions are you hearing from clients? What about in the community, generally?

- How would you describe the community's expectations for the vaccine?
- What about their major concerns?
- As a health professional, do you feel prepared to provide complete and accurate information to people about RTS,S? If not, why not? What would help providers be more prepared?

Section D: Perceptions about RTS,S Partial Protection (≈15 min)

I'd like to finish our discussion with some questions about RTS,S efficacy. As we know, no vaccine is perfect. In the case of RTS,S, studies show that it is 40% effective if a child receives all four doses. I find this to be a little abstract….

D1 Perceptions about Partial Protection

D1.1 What does that even mean?, "40% effective with a four-dose schedule"?

D1.2 How do your clients and the community understand this idea of 40% effective?

- How do you describe it when talking to people about RTS,S?

D1.3 Do you feel that this level of protection is a problem for the success of the vaccine introduction? How so? / Why not?

Annex 9: Community Leader/Member R1 Profile Sheet & Interview/FGD Guides

COMMUNITY LEADERS

Background information

**Interviewer**		**Interview date**	______ Day ______ Month ______ Year
**Interview language**			

**Country**	1 = Ghana	2 = Kenya	3 = Malawi
**Community**	1 = Community name<br>2 = Community name<br>3 = Community name	4 = Community name<br>5 = Community name<br>6 = Community name	7 = Community name<br>8 = Community name<br>9 = Community name

**Community Leader ID#**	____ Country	____ Community	_____ Group	____ ____ ____ Individual

**Participant Information**	
**Organization:**	
**Role/title:**	
**Sex:**	1 = male 2 = female
**Age:**	
**Education**	__________ (years completed)

Interview guide

1. Please describe your role in health activities in your community.
    - How do you interact with/collaborate with the health communication team?
    - How do you interact with/collaborate with the health facility?
    - What is your role when a new service is introduced? Can you please describe an example?

2. How would you describe the impact of malaria on children's health in your community?
    - Why do you say this?
    - What are the major initiatives in the community to prevent malaria?
    - What role have you played in supporting these initiatives?
    - How do you feel about their effectiveness? What could lead to improvements?

3. What about vaccination services? Please describe your role in promoting vaccinations. Please use actual examples if possible.

4. Have you ever been involved informing or educating the public about a new vaccine that is being introduce? *If yes*: Please tell me about that experience.

5. Have you heard about the new malaria vaccine called [RTS,S]?
   If yes:
   - How did you learn about [RTS,S]?
   - What did you learn about the vaccine?

6. Have you been involved / will you be involved in the vaccine's promotion and introduction? Please describe your involvement.

7. What do you see as the vaccine's main advantages for child health in your community?

8. What about the challenges or problems with vaccine? How would you describe these?
   - What do you think would help overcome these challenges?

9. Are people in your community talking about [RTS,S]? What are they saying about it?
   - What questions have you heard from people about its:
     - Effectiveness preventing malaria?
     - Safety?
     - Number of shots needed?
     - Vaccination schedule?
   - What are people's main concerns about the vaccine?

10. What are your major concerns about the vaccine? Why is this a concern for you?

11. As you may know, for the vaccine to be most effective it requires mothers to bring their children for four vaccination shots, the last one when the child is two years old. What are the best ways to motivate, or help, mothers bring their kids for all four visits?

Annex 10: PCG Interview Tracking Log

Fields in Excel tracking log:

- Country
- Unique ID
- Community
- Round (for cohort sample)
    - Interviewed by
    - Date conducted
- Audiofile name
- Transcription/translation
    - Date completed
    - QC conducted by
    - QC date completed
    - Transcription file name
- Archive data
    - In-country
    - HUS Global

Annex 11: Health Leader & Manager Interview Tracking Log

Fields in Excel tracking log:

- Country
- Unique ID
- Round
- Interviews
    - Interviewed by
    - Date conducted
- Audiofile name
- Transcription/translation
    - Date completed
    - QC conducted by
    - QC date completed
    - Transcription file name
- Archive data
    - In-country
    - HUS Global

Annex 12: Health Provider Focus Group Tracking Log

Fields in Excel tracking log:

- Country
- Group Unique ID
- Community
- Round
- Discussion
    - Moderated by
    - Date conducted
    - # discussants
- Audiofile name
- Transcription/translation
    - Date completed
    - QC conducted by
    - QC date completed
    - Transcription file name
- Archive data
    - In-country
    - HUS Global

Annex 13: Community Leader Tracking Log

Fields in Excel tracking log:

INDIVIDUAL INTERVIEWS

- Country
- Unique ID
- Round
- Interviews
    - Interviewed by
    - Date conducted
- Audiofile name
- Transcription/translation
    - Date completed
    - QC conducted by
    - QC date completed
    - Transcription file name
- Archive data
    - In-country
    - HUS Global

FOCUS GROUP DISCUSSIONS

- Country
- Group Unique ID
- Community
- Round
- Discussion
    - Moderated by
    - Date conducted
    - # discussants
- Audiofile name
- Transcription/translation
    - Date completed
    - QC conducted by
    - QC date completed
    - Transcription file name
- Archive data
    - In-country
    - HUS Global

Annex 14: Male Household Head Focus Group Tracking Log

Fields in Excel tracking log:

- Country
- Group Unique ID
- Community
- Round
- Discussion
    - Moderated by
    - Date conducted
    - # discussants
- Audiofile name
- Transcription/translation
    - Date completed
    - QC conducted by
    - QC date completed
    - Transcription file name
- Archive data
    - In-country
    - HUS Global

Annex 15: Female Elder Focus Group and Individual Interview Tracking Log

Fields in Excel tracking log:

INDIVIDUAL INTERVIEWS

- Country
- Unique ID
- Round
- Interviews
  - Interviewed by
  - Date conducted
- Audiofile name
- Transcription/translation
  - Date completed
  - QC conducted by
  - QC date completed
  - Transcription file name
- Archive data
  - In-country
  - HUS Global

FOCUS GROUP DISCUSSIONS

- Country
- Group Unique ID
- Community
- Round
- Discussion
  - Moderated by
  - Date conducted
  - # discussants
- Audiofile name
- Transcription/translation
  - Date completed
  - QC conducted by
  - QC date completed
  - Transcription file name
- Archive data
  - In-country
  - HUS Global

Annex 16: Transcription Guide

RTS,S Healthcare Utilization Study Transcription Template for Individual Interviews

Overall Instructions
Begin the transcription from audio-taped interviews with the first interview question. Pleasantries, greetings, and informed consent processes do not need to be transcribed. Transcribe interview exchanges in full, including interviewer questions and probes and participant replies. Add details of the interview context (interruptions, background disturbance, etc.) to help the reader understand exchanges of meanings in the interview in brackets [. . .].

If the recording is not audible, indicate this in brackets [. . .].

Translation Instructions
Transcribe interviews that are conducted in English verbatim. Translate interviews conducted in a local language into English in the transcription process. Whenever possible, translate as the reply as literally as possible from the local language to English. However, if the intended meaning of the respondent is lost or distorted through literal translation, **translate for meaning**. If you are unsure how to translate a passage where literal translation is not accurate, consult with colleagues to get additional opinions and to come to consensus.

Anonymization Instructions
Remove all personal identifiers from the transcription in the process of transcribing. This includes names, places, jobs, events, etc. that can be linked to the individual interviewed. When personal details are removed, indicate this in brackets, e.g., [name of her child].

Transcript Formatting

**Country:**	
**Sample (e.g., PCG, provider):**	
**Unique ID:**	
**Fieldwork Round:**	
**Interview date:**	
**Interviewer:**	
**Translator-transcriber:**	
**Transcription date(s):**	

- At the top of each transcript include the completed table shown above
- Use 12 point font
- Apply one-inch margins on the left and two-inch margins on the right
- Paginate each transcript
- Insert a footer indicating the following: HUS interview with UNIQUE ID (individual or FGD#)
- Indent text for questions with hanging indent of .5
- Indicate interviewer questions with your initials

- Indicate respondent replies with “R” as shown below

II: Question #1 text here.

R: Reply to Q1 here.

II: Probe or clarifying question on Q1.

R: Reply to probe here.

Annex 17: Initial Ordering-Theme Code List

The broad themes below are provided as a starting point for examining textual data. They are not intended to be exclusive or complete, but rather to guide initial data ordering. Definitions will evolve and become more nuanced as the data dictate.

Text units can be assigned to multiple codes. The broad codes below should be adapted to reflect different perspectives of study samples, e.g., health provider issues and concerns versus caregiver and community issues and concerns.

Theme	Definition
EXPOSURE	Exposure to information about and promotion of RTS,S (sub-coding to distinguish initial from subsequent exposure; type, place, and content of the exposure, etc.)
BENEFIT	Perceived benefits of RTS,S (sub-coding to qualify benefits by type, importance, etc.)
DISADVANTAGE	Perceived disadvantages of RTS,S (sub-coding to qualify disadvantages by type, importance, etc.)
PARTIAL PROTECTION	Exposure to and understanding of messages around RTS,S partial protection (sub-coding to qualify completeness of messaging, likely/observed behavioral implications, etc.)
4TH DOSE	Exposure and reaction to messages around need for RTS,S 4th dose (sub-coding to qualify acceptance, behavioral implications, etc.)
ADOPTION	Expressed intentions/observed behaviors to promote or pursue RTS,S vaccination (sub-coding to qualify positive/negative intentions and reasons associated with the intentions)
ADHERENCE	Expressed intentions/observed behaviors to adhere to RTS,S dose schedule (sub-coding to qualify positive/negative intentions and reasons associated with the intentions)
PREVENTION	Expressed intentions/observed behaviors associated with non-RTS,S malaria prevention practices with the vaccine (sub-coding to qualify rationales, sources of information, etc.)
TREATMENT	Expressed intentions/observed behaviors associated with non-RTS,S malaria treatment seeking practices with the vaccine (sub-coding to qualify rationales, sources of information, etc.)
TRUST	Expressed trust RTS,S efficacy, messaging, service delivery (sub-coding to qualify nature and implications of expressed trust)

Annex 18: HUS Partner Roles & Responsibilities Matrix

PATH

Study Title/Role	Name	Role Description
Project Director	Scott Gordon (Seattle)	Overall lead and PATH representative for the MVIP partnership
Cross-country study lead	Jessica Price (Seattle)	Lead study design and conduct, coordinate HUS cross-country partnership, and oversee reporting and deliverables.
Program Associate	Orianne Berraud (Geneva)	Provides programmatic support and coordination to all aspects of study management, oversees the workplan and M&E framework for the project.
Finance and contract	Juliette Arnaud (Geneva)	Provides overall financial management including budget review and expenses monitoring and oversight of partner agreements.
Program Assistant	Michelle Durand (Geneva)	Provides overall administrative support.
Country focal point Kenya	Chris Odero (Nairobi)	In-country point-of-contact for PATH in support of MVIP activities and coordination with governments and WHO offices in-country, providing technical support for planning and implementation of the MVIP and serving as key PATH linkage for in-country coordination bodies.
Country focal point Malawi	Esau Mkisi (Lilongwe)	
Country focal point Ghana	John Bawa (Accra)	
Senior technical advisor Ghana	Antwi-Agyei, Kwadwo Odei (Accra)	

University of Health and Allied Sciences

Study Title/Role	Name	Role Description
Principal Investigator	Margaret Gyapong (Volta)	Lead Researcher and Oversight over Central Region.
Co-PI	Evelyn Korkor Ansah (Accra)	Co-Lead and Oversight over Brong Ahafo Region.
Co-PI	Seth Owusu Agyei (Accra)	Co-Lead and Oversight over Volta Region.
Study Management & contract	Fidelis Anumu / Prof. John Owusu Gyapong will sign the Contract (Accra)	Research Manager.
Study/Field Manager	Kofi Awusabo-Asare	Project Site Manager – Central Region.
Study/Field Manager	Ellis Owusu-Dabo	Project Site Manager – Brong Ahafo Region.
Study/Field Manager	Phidelia Doegah	Project Site Manager - Volta Region.

Study Title/Role	Name	Role Description
Financial Manager	Charity Dzormeku (Accra)	Oversees and ensures financial management of the study.

Liverpool School of Tropical Medicine (LSTM & LSTM-Kenya), the Kenya Medical Research Institute (KEMRI), the US Centers for Disease Control and Prevention (CDC & KEMRI/CDC), and the London School of Hygiene and Tropical Medicine (LSHTM)

Study Title/Role	Name	Role Description
Principal Investigator	Jenny Hill (LSTM - Liverpool)	Provides overall responsibility for managing the team, delivering the study's outputs, and PATH liaison.
QL methodology Advisor	Jayne Webster (LSHTM Centre for Evaluation – London)	Supports methodological development of qualitative longitudinal study approaches and study design and analysis.
Principal Research Officer	Dr Simon Kariuki (KEMRI, Kisumu)	Oversees and provides scientific leadership and administrative support to the research team.
Site PI or Study/Field Manager	George Okello (KEMRI - Kisumu)	Leads social scientist coordinating field work in western Kenya. He will be the site co-Principal Investigator responsible for obtaining local ethical clearances, stakeholder engagement and overall study coordination including recruitment, training and regular supervision of the research team. He will be responsible for the setup of data management and storage, and for overseeing data quality assurance systems, and contribute to data analysis, report writing and dissemination.
Contract & Finance	Tracy Seddon (LSTM – Liverpool)	Provides financial management of the study.
Programme Manager	Benta Kamire (Kenya-CDC-LSTM)	Oversees and ensures financial management of the study.
Behavioral Epidemiologist	Mohammed Jalloh (DCD, Atlanta)	Provides research methodology and expertise to the study implementation.

Malawi-Liverpool-Wellcome Trust Clinical Research Programme (MLW), University of Malawi, College of Medicine, Liverpool School of Tropical Medicine

Study Title/Role	Name	Role Description
Principal Investigator	Nicola Desmond (Blantyre)	Team Leader, overall responsibility for the research, overseeing and delivering study outputs and outcomes, and serving as liaison to PATH.

Study Title/Role	Name	Role Description
Co-PI	Linda Nyondo- Mipando (Blantyre)	Leads research question I: implementation processes. Joint development of ethics review proposals, budget management, reporting and dissemination. Collection of data at district and facility level.
Co-PI	Kate Gooding (Blantyre)	Lead research question II: impact on household utilization strategies. Joint development of ethics review proposals, budget management, reporting and dissemination.
Administrative Assistant	Mtundu Khongono (MLW, Blantyre)	Provides administrative assistance to the Malawi study team.
Financial Manager	Asimenye, Lucy Kayuni (MLW, Blantyre)	Oversees and ensures financial management of the study.
Head of Grants LSTM	Helen McCormack (LSTM, Liverpool)	Oversees contracting and finance processes.

REFERENCES

1. World Health Organization, *World Malaria Report*. 2014, Geneva, Switzerland.
2. World Health Organization, *World Malaria Report 2017*. 2016: Geneva.
3. Atieli, H., et al., *Insecticide-treated net (ITN) ownership, usage, and malaria transmission in the highlands of western Kenya; 2011, 4:113.* Parasites & Vectors, 2001. **18**(4): p. 113.
4. Ernst, K.C., et al., *Comparing ownership and use of bed nets at two sites with differential malaria transmission in western Kenya.* Malaria Journal, 2016. **15**: p. 2016.
5. Pulford, J., et al., *Reported reasons for not using a mosquito net when one is available: a review of the published literature.* Malaria Journal, 2011. **10**: p. 83.
6. World Health Organization, *Global plan for insecticide resistance management in malaria vectors*. 2012, WHO: Geneva.
7. The malERA Refresh Consultative Panel on Insecticide and Drug Resistance, *malERA: An updated research agenda for insecticide and drug resistance in malaria elimination and eradication.* PLoS Med, 2017. **14**(11): p. e1002450.
8. The RTSS Clinical Trials Partnership, *First Results of Phase 3 Trial of RTS,S/AS01 Malaria Vaccine in African Children.* New England Journal of Medicine, 2011. **365**(20): p. 1863-1875.
9. The RTS,S Clinical Trials Partnership, *A Phase 3 Trial of RTS,S/AS01 Malaria Vaccine in African Infants.* New England Journal of Medicine, 2012. **367**(24): p. 2284-2295.
10. Joint Technical Working Group and WHO Secretariat, *Background Paper on teh RTS,S/AS01 Malaria Vaccine*. 2015.
11. The RTS,S Clinical Trials Partnership, *Efficacy and safety of RTS,S/AS01 malaria vaccine with or without a booster dose in infants and children in Africa: final results of a phase 3, individually randomised, controlled trial.* The Lancet, 2015. **386**: p. 31-45.
12. World Health Organization, *Malaria vaccine: WHO position paper.* Weekly Epidemiological Record, 2016. **4**(91): p. 33-52.
13. World Health Organization, *Malaira Vaccine Implementation Programme (MVIP), Briefing Document*. 2017: Geneva.
14. Bingham, A., et al., *Community perceptions of malaria and vaccines in two districts in Mozambique.* Malaria Journal, 2012. **11**.
15. PATH Malaria Vaccine Initiative, *Research on Community Perceptions of Vaccines and Malaria in Four African Countries*. 2014.
16. Meñaca, A., et al., *Factors Likely to Affect Community Acceptance of a Malaria Vaccine in Two Districts of Ghana: A Qualitative Study.* PLOS One, 2014. **9**(10): p. e109707.
17. Ojakaa, D.I., et al., *Community perceptions of malaria and vaccines in the South Coast and Busia regions of Kenya.* Malaria Journal, 2011. **10**.
18. Sallis, J., N. Owen, and E. Fisher, *Ecolgoical Models in Health Behavior*, in *Health Behavior and Education: Theory and Practice*, K. Glanz, B. Rimer, and K. Viswanath, Editors. 2008, Jolley-Bass: San Francisco. p. 464-485.
19. Bingham, A., et al., *An Approach to Formative Research in HPV Vaccine Introduction Planning in Low-Resource Settings.* The Open Vaccine Journal, 2009. **2**: p. 1-16.
20. PATH, *Conducting Formative Research for HPV Vaccination Program Planning: Practical Experience from PATH*. 2012: Seattle, WA.

21. Neale, B., *What is Qualitative Longitudinal Research?* 2018 (forthcoming), London: Bloomsbury.
22. Leung, L., *Validity, reliability, and generalizability in qualitative research.* Journal of Family Medicine and Primary Care, 2015. **4**(3): p. 324-327.
23. Onwuegbuzie, A.J. and N.L. Leech, *Sampling Designs in Qualitative Research: Making the Sampling Process More Public.* The Qualitative Report, 2007. **12**(2): p. 238-254.
24. Polit, D.F. and C.T. Beck, *Generalization in quantitative and qualitative research: Myths and strategies.* International Journal of Nursing Studies, 2010. **47**: p. 1451-1458.
25. Sandelowski, M., S. Docherty, and C. Emden, *Qualitative Metasynthesis: Issues and Techniques.* Research in Nursing & Health, 1997. **20**: p. 365-371.
26. Schofield, J.W., *Increasing the generalizability of qualitative research*, in *Qualitative inquiry in education: The continuing debate*, E. Eisner and A. Peshkin, Editors. 1993, Teachers College Press: New York. p. 201-232.
27. Herriott, R.E. and W.A. Firestone, *Multisite Qualitative Policy Research: Optimizing Description and Generalizability.* Educational Research, 1983. **12**(2): p. 14-19.
28. Barbour, R.S., *Checklists for improving rigour in qualitative research: a case of the tail wagging the dog?* British Medical Journal, 2001. **322**: p. 1115-1117.
29. Kitto, S.C., J. Chesters, and C. Grbich, *Quality in qualitative research: Criteria for authors and assessors in the submission and assessment of qualitative research articles for the Medical Journal of Australia.* Medical Journal of Australia, 2008. **188**(4): p. 243-246.
30. Meyrick, J., *What is Good Qualitative Research? A First Step towrards a Comprehensive Approach to Judging Riquor/Quality.* Journal of Health Psychology, 2006. **11**(5): p. 799-808.
31. Palinkas, L.A., et al., *Purposeful sampling for qualitative data collection and analysis in mixed method implementation research.* Adm Policy Ment Health, 2015. **42**(5): p. 533-544.
32. Sandelowski, M., *Sample Size in Qualitative Research.* Research in Nursing & Health, 1995. **18**: p. 179-183.
33. Onwuegbuzie, A.J. and K.M. Collins, *A Typology of Mixed Methods Sampling Designs in Social Science Research.* The Qualitative Report, 2007. **12**(2): p. 281-316.
34. Kleinman, A., *Patients and Healers in the Context of Culture: An Exploration of the Borderline between Anthropolog, Medicine, and Psychiatry*. 1980, Berkeley: University of California Press.
35. Agar, M.H., *The Professional Stranger: An Informal Introduction to Ethnography*. 1980, San Diego: Academic Press, Inc.
36. Spradley, J.P., *Participant Observation*. 1980, New York: Harcourt Brace Jovanovich College Publishers.
37. Maxwell, J.A., *Using Qualitative Methods for Causal Explanation.* Field Methods, 2004. **16**(3): p. 243-264.
38. Malterud, K., *Systematic text condensation: A strategy for qualitative analysis.* Scandinavian Journal of Public Health, 2012. **40**: p. 795-805.
39. Kvale, S., *InterViews: Learning the craft of qualitative research interviewing*. 3rd ed. 2015, Los Angeles: Sage Publications.
40. Ayres, L., K. Kavanaugh, and K.A. Knafl, *Within-Case and Across-Case Approaches to Qualitative Data Analysis.* Qualitative Health Research, 2003. **13**: p. 871-883.

41. Sandelowski, M., *One is the Liveliest Number: The Case Orientation of Qualitative Research.* Research in Nursing & Health, 1996. **19**: p. 525-529.
42. Neale, B., et al. *Researching the lives of young fathers: the Following Young Fathers study and dataset Working Paper no. 8*. 2015; Available from: www.followingfathers.leeds.ac.uk/findingsandpublications.
43. Saldaña, J., *Longitudinal Qualitative Research: Analyzing Change Through Time*. 2003, Walnut Creek: Altamira Press.
44. Smith, N., *Cross-sectional profiling and longitudinal analysis: Research notes on analysis in the longitudinal qualitative study, 'Negotiating Transitions to Citizenship'.* International Journal of Social Research, 2003. **6**(3): p. 273-277.
45. Mukumbang, F.C., et al., *An exploration of group-based HIV/AIDS treatment and care models in Sub-Saharan Africa using a realist evaluation (Intervention-Context-Actor-Mechanism-Outcome) heuristic tool: a systematic review.* Implementation Science, 2017. **12**(107).
46. Van Belle, S.B., et al., *How to develop a theory-driven evaluation design? Lessons learned from an adolescent sexual and reproductive health programme in West Africa.* BMC Public Health, 2010. **10**: p. 741.
47. Sandelowski, M., *Real Qualitative Researchers Do Not Count: The Use of Numbers in Qualitative Research.* Research in Nursing & Health, 2001. **24**: p. 230-240.
48. Fernald, D.H. and C.W. Duclos, *Enhance Your Team-Based Qualitative Research.* Annals of Family Medicine, 2005. **3**(4): p. 360-364.
49. Poortman, C. and K. Schildkamp, *Alternative quality standards in qualitative research?* Quality and Quantity, 2012. **46**: p. 1727-1751.
50. Laditka, S.B., et al., *Methods and Management of the Healthy Brain Study: A Large Multisite Qualitative Research Project.* The Gerontologist, 2009. **49**(1): p. S18-S22.
51. Ansah, E.K. and T. Powell-Jackson, *Can we trust measures of healthcare utilization from household surveys?* BMC Public Health, 2013. **13**: p. 853.
