# Supplementary material for: Acceptance of and Adherence to a Four-Dose RTS,S/AS01 Schedule: Findings from a Longitudinal Qualitative Evaluation Study for the Malaria Vaccine Implementation Programme
Source: Vaccines (Basel). 2023 Dec 1;11(12):1801. doi: 10.3390/vaccines11121801 (PMC10747521; doi:10.3390/vaccines11121801)
Supplement: Supplementary file 1 [file vaccines-11-01801-s001.zip › Suppl 3_Profile Sheet.pdf]

HUS Primary Caregiver Profile Sheet – ROUND 3

v. 27 January 2020

**Q1. Date of interview**				**Q2. Interview round**	1 = Round 1<br>2 = Round 2<br>3 = Round 3<br>4 = cross-sectional
	M M	D D	Y Y		

**Q3. Interviewer**	

	**Q4. Interview Language**
1	X
2	Y
3	Z
4	D
5	E

**Q5. Community**			
	X = Community name	X = Community name	X = Community name
	X = Community name	X = Community name	X = Community name
	X = Community name	X = Community name	X = Community name

**PCG ID#**	G / K / M	PCG	R2	___ ___	___ ___ ___
	Country	Group	Round	Community	Individual

Confirmation of PCG and RTS,S-eligible Child

Follow country-specific procedure to confirm:

1. That the same individual (PCG) is being interviewed.

2. That the RTS,S-eligible child is still living. **If the child is deceased, do not proceed with the interview. Follow country-specific guidance pertinent to this situation.**

3. That the mother is reminded that our questions concern the RTS,S-eligible child.

PCG Background – *ASK ALL PCGs*

1.	PCG's Sex: 1 ☐ Male 2 ☐ Female

2.	Are you the head of the household? 1 ☐ Yes 2 ☐ No
3.	Please tell me how old you are. __________ (write in completed years)
4.	How many years have you attended school? _________ (write in completed years)
5.	How long have you lived in COMMUNITY X?<br>_________ (write in completed years) _________ (write in months if less than 1 year)
6.	What is your relation to RTS,S-ELIGIBLE CHILD?<br>1 ☐ Mother<br>2 ☐ Father<br>3. ☐ Sibling<br>4. ☐ Aunt/Uncle ***(expand to capture maternal/paternal aunt/uncle if needed)***<br>5. ☐ Grandparent ***(expand to capture maternal/paternal aunt/uncle if needed)***<br>6. ☐ Other
7.	When was RTS,S-ELIGIBLE CHILD born?<br>____ ____ / ____ ____ / ____ ____ ____ ____<br>Day / Month / Year
8.	RTS,S-ELIGIBLE CHILD's Sex: 1 ☐ Male 2 ☐ Female
9.	Including RTS,S-ELIGIBLE CHILD, how many children do you have? _________
10.	Among all your children, how many are under the age of five years old? _________

PCG Background	
1.	PCG's home address has changed since the last interview:<br><br>1 ☐ yes<br><br>2 ☐ no
2.	Where do you take your child(ren) for vaccination?<br><br>1 ☐ Usually a community outreach service<br><br>2 ☐ Usually at a health facility<br><br>______________________________<br>(Write in name of the facility)
3.	How do you usually get there? *(Select only one option.)*<br><br>1 ☐ Walk<br><br>2 ☐ Bus/taxi-bus<br><br>3. ☐ Motorcycle<br><br>4. ☐ Bicycle<br><br>5. ☐ Tricycle<br><br>6. ☐ Other
4.	About how long does it take you to get to VACCINATION SITE by TRANSPORTATION MODE?<br><br>________ (write in minutes)
5.	What is your current marital status?<br><br>1 ☐ Currently married<br><br>2 ☐ Cohabitating with a regular partner<br><br>3 ☐ Divorced/separated and living apart<br><br>4 ☐ Widowed

	5 ☐ Never married
6.	In your household, what is the main source of income? I'm going to read you a list and you can tell me if any item on this applies to you:<br>1 ☐ Salaried employment by an adult household member<br>2 ☐ Fishing<br>3. ☐ Farming<br>4. ☐ Small commerce<br>5. ☐ Other
7.	What is your religion?<br>1 ☐ Christian<br>2 ☐ Islam<br>3 ☐ Traditionalist<br>4 ☐ Other<br>5 ☐ No religion
8.	Did [RTS,S-eligible child] sleep under a bed net last night?<br>1 ☐ yes<br>2 ☐ no
9.	How often does the [RTS,S-eligible child] sleep under a bed net?<br>1 ☐ Every time s/he is in bed<br>2 ☐ Every time s/he is in bed, but only in the rainy season<br>3 ☐ Most of the time s/he is in bed<br>4 ☐ Some of the time s/he is in bed<br>5 ☐ Rarely<br>6 ☐ Don't know

10.	Since the last time we visited, has [RTS,S-eligible child] had malaria? (If it is a new PCG, ask since you took over the role of caring for RTS,S eligible child, has the child had Malaria?)<br><br>1 ☐ yes<br><br>2 ☐ no → ***Go to 12.***
11.	Was the [RTS,S-eligible child] tested and confirmed to have malaria by a health worker?<br><br>1 ☐ yes<br><br>2 ☐ no
12.	Has any other of your children who are younger than five years old had malaria since the last time we visited? (If the RTS,S eligible child is the PCGs only child this question will be na)<br><br>1 ☐ yes<br><br>2 ☐ no → ***Complete the Vaccination History Sheet***<br><br>**3. ☐ na**
13.	Were the other children who had malaria tested and confirmed to have malaria by a health worker? (If the RTS,S eligible child is the PCGs only child this question will be na)<br><br>1 ☐ yes<br><br>2 ☐ no<br><br>**3. ☐ na**<br><br>***Complete the Vaccination History Sheet***
