# Supplementary material for: Acceptance of and Adherence to a Four-Dose RTS,S/AS01 Schedule: Findings from a Longitudinal Qualitative Evaluation Study for the Malaria Vaccine Implementation Programme
Source: Vaccines (Basel). 2023 Dec 1;11(12):1801. doi: 10.3390/vaccines11121801 (PMC10747521; doi:10.3390/vaccines11121801)
Supplement: Supplementary file 1 [file vaccines-11-01801-s001.zip › Suppl 4_Vaccination History Sheet.pdf]

Vaccination History Sheet – R3

**PCG ID#**	Country	____ ____ Community	PCG Group	____ ____ ____ Individual

**Date of interview**	DD	MM	YY	**Interview round**	1 = Round 1<br>2 = Round 2<br>3 = Round 3

**Child's vaccination card #:**		**Type of card seen**	1 = Child Health Record Book<br>2 = Yellow card<br>3 = Piece of paper / other documentation<br>4 = No card seen [***GO TO SECTION H2***]<br>5 = Maternal & Child Health Record Book

**H1**	**Vaccination History**							
	**Vaccine**	**Received?**	**Date indicated in Vaccination card**					
1	Malaria RTS,S (1)	1 = yes<br>0 = no	D	D	M	M	Y	Y
2	Malaria RTS,S (2)	1 = yes<br>0 = no	D	D	M	M	Y	Y
3	Malaria RTS,S (3)	1 = yes<br>0 = no	D	D	M	M	Y	Y
4	Malaria RTS,S (4)	1 = yes<br>0 = no	D	D	M	M	Y	Y
5	BCG	1 = yes<br>0 = no	D	D	M	M	Y	Y
6	Pentavalent (1)<br>DTP/Hib/HepB	1 = yes<br>0 = no	D	D	M	M	Y	Y
7	Pentavalent (2)<br>DTP/Hib/HepB	1 = yes<br>0 = no	D	D	M	M	Y	Y
8	Pentavalent (3)<br>DTP/Hib/HepB	1 = yes<br>0 = no	D	D	M	M	Y	Y
9	Measles (1)	1 = yes<br>0 = no	D	D	M	M	Y	Y
10	Measles (2)	1 = yes						

		0 = no	D	D	M	M	Y	Y

H2 Vaccination Recall – *ask only if the health card is not available*

Question	Response
1. Has this child ever been vaccinated at a health facility or site in the community?	1 = yes 0 = no<br>99 = don't know
2. How old was the child at the last vaccination visit?	__________ weeks<br>__________ months
3. As far as you know, has your child received all of the recommended vaccinations up to the current age of the child?	1 = yes 0 = no<br>99 = don't know
4. Were you ever told by the vaccination staff that the child was given the new malaria vaccine?	1 = yes 0 = no<br>99 = don't know
5. How many times has the child received the new malaria vaccine?	_____________ times
6. Other than the malaria vaccine, how many times has the child received vaccination at a health facility or site in the community?	_____________ times

H3 Perceived Adverse Events from RTS,S

Thank you for showing me [RTS,S-eligible child's] vaccination card.

H3.1 Please tell me how your child felt after receiving the malaria vaccine?

No side effects observed → ***proceed to interview guide***
Side effects observed → ***proceed with questions***

H3.2 What did you do in response to how your child felt after receiving the malaria vaccine?

- Why did you take these steps?
- What did health workers tell you to do in case any of these things happened?
- Did you take him/her to a health provider? What did they do?

H3.3 How will this experience affect your decision to vaccinate your children again? Why?/Why not?

Check the vaccination card for receipt of RTS,S vaccine.

No vaccines received → ***end the interview***
RTS,S vaccine received → ***end the interview***
No RTS,S vaccine received; other vaccines received → ***end the interview***
