# Supplementary material for: Acceptance of and Adherence to a Four-Dose RTS,S/AS01 Schedule: Findings from a Longitudinal Qualitative Evaluation Study for the Malaria Vaccine Implementation Programme
Source: Vaccines (Basel). 2023 Dec 1;11(12):1801. doi: 10.3390/vaccines11121801 (PMC10747521; doi:10.3390/vaccines11121801)
Supplement: Supplementary file 1 [file vaccines-11-01801-s001.zip › Suppl 5_R1 Interview Guide.pdf]

HUS PCG Interview Guide – v 4 February 2019

Section W: Warm Up

Thank you for your replies. Before we start talking about child health issues, I want to learn a little bit about you.

W1 Tell me about yourself and your family.

Probe: What region are you from?
How many children do you have? How old are they?
Etc.

Take around 5 min getting to know a little about the participant and allow her time to settle in and feel comfortable talking with you.

W2 What are the most common child health problems in your community?

- As a mother/caregiver of young children, what are your main concerns about your child's health?
- Which are the serious problems? How so? Why do you say this?

W3 In general, how do you learn about child health issues and services?

- From whom? How?
- Who do you trust most to learn about child health? Why?

Section M: Malaria Perceptions & Behavior

I'd now like to ask you some questions about malaria specifically. /// You just mentioned malaria as a child health concern in your community. I'd now like to ask you some questions specifically about malaria.

M1 Malaria Perceptions

M1.1 As a problem for health in your community, how would you describe the importance/significance of malaria in your community?

- Why do you say this?
- Who/which people in your community are commonly affected? How so? Why do you say this?
- What do people typically do when they have malaria? What if it doesn't go away?

M1.2 What about in your household? How would you describe the importance/significance of malaria in your own household/family?

- Why do you say this?
- Who in your family is most affected by malaria? How so? Why do you say this?

M2 Malaria Experience

M2.1 Now I want to focus on RTS,S-ELIGIBLE CHILD. Has s/he ever had malaria?

No → That's good news. I'd like you to think about another one of your children (or close relative who is a young child) who recently had malaria. Take a minute to remember the most recent incident you can recall.

- How old was the child?

Yes → I'm sorry to hear that. I'd like to learn more about this malaria episode, so please take a moment to remember it.

- How old was the child?

M2.2 I'd like to learn about the what happened during the illness of this child. Can you describe for me what happened during the illness from beginning to end. Please start from when you first noticed that you child had a problem.

Allow the participant to describe the episode fully, then proceed with questions.

- What made you think the child had malaria? [main intent: symptoms recognition; local perceptions about malaria symptoms]
- What did you do? (***Probe:*** Where did you go for help?)
- The place you went for help, what did they do
- Did your child get tested for malaria?
- ***If tested***, After the test, what did the health workers tell you?
- How long did it take for your child to feel better?

M2.3 What else did you do? // Then what happened? [main intent: elicit multiplicity of actions taken to resolve the illness]

- Did you do anything else? Please tell me more.

M2.4 What did the child's illness cost your household to take care of? [main intent: assess the material burden of treatment seeking, which may be greater with multiple treatment actions]

- At any time during the illness, did you lose wages/earnings in order to treat or take care of your child? ***If yes ask***: Please tell me more about this.
- At any time during the illness, did you have to use savings, borrow money, or sell anything in order to treat your child? ***If yes ask***: Please tell me more.
- Did you have to get a loan from anyone to care for you child?

M3 Malaria Prevention

M3.1 Thinking about this malaria episode, how do you think it could have been prevented?

- Why? / Why not?
- After the child's illness episode, what have you done to protect her/him from getting malaria?

M3.2 Of the things you mentioned that can prevent malaria, which of these were you doing before your child got malaria?

- Please elaborate. Why do you do these things?

M3.3 Tell me about how you use bed nets in your household.

- Do you have bed nets in your household? If no, why not?
- Did the [RTS,S eligible child] sleep under a bed net last night? If no, why not?

Section V: Vaccination Perceptions & Behavior

V1 Vaccination Beliefs & Information Sources

V1.1 How would you describe the purpose of vaccinations for child health?

- Why do you say that? Please tell me more.
- What are your main concerns about your child/ren getting a new vaccine?

V1.2 How do you learn about vaccines?

- Can you give me a specific example?

V1.3 Now let's talk about a vaccination for malaria. How do you feel about a vaccine that helps prevent malaria?

V2 Vaccination Visit Experience

Thank you very much for sharing those thoughts with me. Now I'd like to hear about your experiences taking your child/ren for vaccination.

V2.1 Since [RTS,S-eligible child] was born, have you taken her/him for vaccination?

yes → ***go to question V2.3***
no → ***proceed to next question***

V2.2 What about another one of your children, have you taken one of them for vaccination?

yes → ***proceed with next question***
no → ***skip to question V2.6***

V2.3 Thank you. I now want to ask you some questions about your most recent vaccination visit with [RTS,S-eligible child / your child]. Please take a moment to think about that visit. Can you please tell me about the vaccination visit from beginning to end.

Allow the caregiver to describe the experience fully then probe:

- About when did this vaccination visit happen?
- Tell me more about how you felt about the convenience and availability of services. Why? Can you please elaborate?
- How about staff attitudes? For example, how would you describe the welcome you received and staff politeness? What else?/Anything else?
- What are your thoughts about the physical conditions at the service site? Think about cleanliness and comfort, things like that. Tell me more./Please be more specific.
- What were your impressions about the vaccine providers' knowledge and skills? Why do you say this?

V2.4 In relation to this same vaccination visit, please tell me about all the health information you received during the visit.

Allow the caregiver to describe information received then proceed with questions.

- What did you learn about possible adverse events from the vaccines? ***If anything ask***: What did the provide advise you to do in the event that you observed an adverse event?
- What did the providers tell you about the benefits of vaccinations for children?
- What did they say about bringing the child back for follow-up? Please be specific.

- Did you have any questions about the vaccine(s) that didn't get answered during the visit? Please tell me what these questions are?
- Other than information about vaccinations, what other information about child health did you learn during this visit? Please elaborate.

V2.5 Overall, how satisfied are you with the care your child received during this visit?

- Why do you feel this way? Please be specific.
- What would improve the vaccination services for you? What else?
- Among all of the things you've just described, which would say is the important thing? Why did you pick that one.

Skip to Section V3

V2.6 No problem. Please tell me about what you've heard from other people about vaccines and vaccination services.

- Has anything you've heard about vaccines or vaccination services discouraged you from taking your own child for vaccination? ***If yes ask***: Please tell me more.

V2.7 What are the reasons you have never taken your child for vaccination?

V2.8 What would encourage or help you take your child for vaccination? Please elaborate.

Skip to Question V3.5

V3 Vaccination Decision Making

Thank you. I have just a few more questions about this same vaccination visit.

V3.1 Please explain to me how you arrived at the decision to take the child for vaccination.

Allow the caregiver to describe the decision-making process fully then proceed with probes for any unanswered questions.

- I want you to think about what you considered when you decided to take the child for vaccination. What were the things you considered?
- Why were these things considered?
- What were the main motivations for you to decide to take the child?

V3.2 Who was involved in making the decision?

- How were they involved?
- What would you have done if [person involved] refused to allow you to take your child for vaccination?

V3.3 Once the decision was made to take the child for vaccination, tell me how you prepared to go to the clinic for vaccination.

Allow the caregiver to describe the preparation process fully then proceed with probes for any unanswered questions.

- What did you do to remember the date?
- How did you manage the visit with work and other duties?
- What challenges did you face in getting to the health facility for vaccination?
- Did anyone help you get to the health facility for vaccination? Please explain.

V3.4 Sometimes there can be challenges and someone decides not to go for vaccination. Can you tell me about a time when this has happened?

- Why did you decide not to go?
- Who was involved in the decision not to go?
- *(if never decided not to go for vaccination):* Can you tell me about a time when you delayed going for vaccination? Why did you delay going for vaccination?

Skip to Section R

V3.5 Please explain to me how your household arrives at a decision to take the child for a health service.

Allow the caregiver to describe the decision-making process fully then proceed with probes for any unanswered questions.

- I want you to think about what you consider when you decided to take the child for a health service. What things do you consider?

- Why are these things considered?

V3.6 Who is involved in making the decision?

- How are they involved?
- What would you have done if you disagreed with [person involved] decision?

Section R: RTS,S Exposure

R.1 Have you heard about the new malaria vaccine called [RTS,S]?

No → ***Skip to Section H***
Yes → ***Proceed with questions***

R.2 What have you heard about [RTS,S]?

- What else?
- What are people in the community saying about it?
- What questions do you, or other people in your community, have about [RTS,S]?
- Anything else?

R.3 Where did you hear about [RTS,S]?

- ***Probe for exposure to local campaigns***, e.g.: radio, posters, community health workers, etc.
- Who have you talked to about the [RTS,S]?
- What did you talk about specifically?
- How did the conversation about [RTS,S] come up?

R.4 What is the best way for people in your community to learn about this new vaccine? Why do you say that?

Proceed to Section H

Section H1: Vaccination History Sheet

**PCG ID#**	Country	____ ____ Community	PCG Group	____ ____ ____ _Individual

**Date of interview**	DD	MM	YY	**Interview round**	1 = Round 1<br>2 = Round 2<br>3 = Round 3

**Child's vaccination card #:**		**Type of card seen**	1 = Child Health Record Book<br>2 = Yellow card<br>3 = Piece of paper / other documentation<br>4 = No card seen [***GO TO SECTION H2***]

	**Vaccine**	**Received?**	**Date indicated in Vaccination card**
1	Malaria RTS,S (1)	1 = yes<br>0 = no	____ ____ ____ ____ ____ ____<br>D D M M Y Y
2	Malaria RTS,S (2)	1 = yes<br>0 = no	____ ____ ____ ____ ____ ____<br>D D M M Y Y
3	Malaria RTS,S (3)	1 = yes<br>0 = no	____ ____ ____ ____ ____ ____<br>D D M M Y Y
4	Malaria RTS,S (4)	1 = yes<br>0 = no	____ ____ ____ ____ ____ ____<br>D D M M Y Y
5	BCG	1 = yes<br>0 = no	____ ____ ____ ____ ____ ____<br>D D M M Y Y
6	Pentavalent (1)<br>DTP/Hib/HepB	1 = yes<br>0 = no	____ ____ ____ ____ ____ ____<br>D D M M Y Y
7	Pentavalent (2)<br>DTP/Hib/HepB	1 = yes<br>0 = no	____ ____ ____ ____ ____ ____<br>D D M M Y Y
8	Pentavalent (3)<br>DTP/Hib/HepB	1 = yes<br>0 = no	____ ____ ____ ____ ____ ____<br>D D M M Y Y
9	Measles (1)	1 = yes<br>0 = no	____ ____ ____ ____ ____ ____<br>D D M M Y Y
10	Measles (2)	1 = yes<br>0 = no	____ ____ ____ ____ ____ ____<br>D D M M Y Y

H2 Vaccination Recall

Question	Response
1. Has this child ever been vaccinated at a health facility or site in the community?	1 = yes 0 = no<br>99 = don't know
2. How old was the child at the last vaccination visit?	__________ weeks<br>__________ months
3. As far as you know, has your child received all of the recommended vaccinations up to the current age of the child?	1 = yes 0 = no<br>99 = don't know
4. Were you ever told by the vaccination staff that the child was given the new malaria vaccine?	1 = yes 0 = no<br>99 = don't know
5. How many times were you told the child received the new malaria vaccine?	_____________ times
6. Other than the malaria vaccine, how many times has the child received vaccination at a health facility or site in the community?	_____________ times

H3 Perceived Adverse Events

Thank you for showing me [RTS,S-eligible child's] vaccination card.

H3.1 Please tell me how your child felt during the day after receiving the vaccines?

No side effects observed → ***Skip to Section H4***
Side effects observed → ***proceed with questions***

H3.2 What did you do in response to how your child felt after receiving the vaccines?

- Why did you take these steps?
- What did health workers tell you to do in case any of these things happened?
- Did you take him/her to a health provider? What did they do?

H3.3 Will this experience affect your decision to vaccinate your children again? How so? Why?/Why not?

Check the vaccination card for receipt of RTS,S vaccine.

No vaccines received → ***end the interview***
RTS,S vaccine received → ***end the interview***
No RTS,S vaccine received; other vaccines received → ***proceed with questions***

H4 Missed RTS,S vaccination

Thank you for showing me [RTS,S-eligible child's] vaccination card. I noticed that your child has received some vaccinations but has also missed some vaccinations.

H4.1 What are the reasons you did not take your child for vaccination with the new malaria vaccine?

H4.2 What would encourage or help you take your child for vaccination with the new malaria vaccine? Please elaborate.

END Is there anything else you'd like to share with me today?
