# Supplementary material for: Acceptance of and Adherence to a Four-Dose RTS,S/AS01 Schedule: Findings from a Longitudinal Qualitative Evaluation Study for the Malaria Vaccine Implementation Programme
Source: Vaccines (Basel). 2023 Dec 1;11(12):1801. doi: 10.3390/vaccines11121801 (PMC10747521; doi:10.3390/vaccines11121801)
Supplement: Supplementary file 1 [file vaccines-11-01801-s001.zip › Suppl 5_R2 Interview Guide.pdf]

PCG R2 Interview Flow and Guide, v. 3 March 2020

NOTE: Please complete the Profile Sheet and Vaccination History Sheet before starting this interview. Additionally, please be sure to re-read the previous interview transcript for the selected respondent before conducting interview.

**Warm-up**
Countries devise a warm-up question or two as they see fit. Please train interviewers to keep this section short. NOTE: Warm-up sections will not be needed for cross-country analyses if you don't wish to spend time and resources translating and transcribing this portion of the interview.

**Section 1**	**Malaria Experience**		
**1.**	**You told me earlier that [RTS,S-eligible child/other young child] has:**		
	**. . . suffered from malaria since our last visit to your household.**<br>**PROCEED WITH Q1.1**		**. . . not suffered from malaria since our last visit to your household.**<br>**SKIP TO Q1.4**
1.1	I'd now like to ask you about this last time [RTS,S-eligible child] suffered from malaria. How did you first notice that your child had a problem?<br><br>*Probe to understand what health issues specifically alarmed the mother.*		
1.2	At that time, why did you think the condition your child had was malaria?<br><br>*If mother initially thought it was not malaria, probe for what made mother begin to think it was a case of malaria.*<br><br>*Probe to understand how mothers conceptualize malaria symptoms.*		
1.3	Can you now please describe to me all the things that were done for your child while he/she was sick with malaria? Please start with the very first thing that was done for the child in the beginning and everything else that followed.<br><br>*Probe to get a complete picture of treatment action, from home remedies, drug shop cures, traditional healers, and resort to biomedical services.*		
**MALARIA EPISODE REPORTED:**		**MALARIA EPISODE <u>NOT REPORTED</u>:**	
1.4	Thinking about this episode of malaria, is there anything you could have done to prevent the child getting malaria? Can you please tell me more?	1.4	I'm happy to hear your child has not had malaria recently. Given that malaria is very common in this community, how do you think your child/ren has/have avoided it recently?
	Probe:<br>*Prevention behaviors (or lack thereof) and rationales.*<br><br>*If RTS,S comes up, probe:*<br>*Mother's perceptions about RTS,S effectiveness in preventing malaria and/or mitigating disease severity, mothers perception of partial protection.*		

1.5	Have your ideas about malaria changed in any way since children in your community started receiving the malaria vaccine? Please explain how.<br><br>*Probe for changes pertaining to their own children and malaria in the community generally.*

Section 2	Malaria Prevention		
**2.**	**Earlier you told me that [RTS,S-eligible child]:**<br>**. . . slept under a bed net last night.**		**. . . did not sleep under a bed net last night.**
**SLEPT UNDER BED NET:**		**<u>DID NOT</u> SLEET UNDER BED NET:**	
2.1	Is this typical? Please tell me more about the reason this is.<br><br>What challenges have you experienced in getting your children to consistently sleep under a bed net?<br><br>*Probe for who uses bed nets and how the decision is made, with particular emphasis on the RTS,S child.*	2.1	Please tell me the reasons the [RTS,S-eligible child] didn't sleep under a net last night?<br><br>*Probe for availability of nets, alternate uses of nets.*
2.2	What else do you do to protect your child from getting malaria?		
2.3	What, if anything, has changed in how you protect your child from getting malaria since our last visit to your household? Tell me why that is.<br><br>*Probe for challenges and reasons for any change in practice.*		

Section 3	Exposure to RTS,S Messages and Perceptions
3.1	I now want to ask you about the new malaria vaccine. Can you tell me what you've recently heard about this vaccine? *For each thing heard ask:* Where did you hear this?<br><br>*Probe for messages pertaining to:*<br>• *Benefits of the vaccine*<br>• *Eligibility of child*<br>• *Number and timing of doses*<br>• *Protection*
3.2	*Ask depending on replies to Q3.1*: Have you heard anything negative or scary about the malaria vaccine? What have you heard? From who?/Where?
3.3	From all the people/places you have heard about the malaria vaccine, tell me which do you trust the most?<br><br>*Probe for the reason for the preferred source of information.*

3.4	From everything that you've heard about the malaria vaccine, what has influenced your views about it most?<br><br>• Why has this/have these thing(s) influenced you most? *Probe each.*<br>• What else would you like know about the vaccine?<br>• What would be the best way for you to get your questions answered/ to get the information you want?
3.5	How has your understanding of the malaria vaccine changed since we last visited? What caused this change?<br><br>*Probe for details (who, where, how, what context), for example, about an encounter with someone (provider, other mother..) that caused the PCG to shift her thinking about/understanding of RTS,S.*

**Section 4**	**RTS,S Vaccination Visit**		
**4.**	**I saw from [RTS,S-eligible child's] vaccination card that s/he has received:**<br>**. . . [2 or more] malaria vaccine doses.**		**. . . [1 or fewer] malaria vaccine doses.**
**TWO OR MORE RTS,S DOSES RCV'D:**		**ONE OR FEWER RTS,S DOSES RCV'D:**	
4.1	How did you remember it was time to take [RTS,S-eligible child] for the 2nd/3rd malaria vaccine dose?<br><br>*Probe: did you hear or receive anything to remind you about when to bring the child back?*	4.1	I notice that you missed some of your visits for the malaria vaccine. Please tell me what happened.<br><br>*Probe to understand the main barriers (e.g., access, time, concerns about the vaccine) and ask if there is any difference from when we last spoke.*
4.2	Please describe your experience during the last vaccination visit when your child received the malaria vaccine.<br><br>*Probe for a full description of the visit.*<br><br>*Probe for:*<br>• *What was the overall experience like for you and for your child – positive/negative (how so and why)?*<br>• *What question(s) you asked the provider during this visit, if any?*<br>• *How did the provider answer your question(s)?*	4.2	*Re information barriers (e.g., "I didn't know I supposed to take her/him"), ask*:<br>• How do you normally know that you are supposed to take your child back to the clinic for other routine vaccination doses?<br><br>*Re access barriers (e.g., "I didn't have time"), ask:*<br>• Is this a problem you've experienced with other vaccines? What do you usually do when this happens? Why couldn't you do the same in this instance?<br><br>*Re fears/rumors involved, ask:*

			• Where did you hear this from? / Who's saying this? Do you believe them? Why do you believe them?<br>• What about what health providers are saying? How does this affect your thinking about the malaria vaccine?<br><br>**SKIP TO 4.4**
4.3	Please tell me about how the child felt after receiving the last malaria dose.<br><br>*Probe to learn what the mother did in response to adverse events, if any.*<br><br>*Probe to learn whether the mother felt concerned by the adverse events, if any.*		**SKIP TO 4.4**
4.4	What did you learn about the malaria vaccine from the health provider during your visit to the health clinic?<br><br>*Probe to understand type and key messages of information received.*		
4.5	Please tell me about any worries you have about your child receiving the vaccine?		
4.6	Please tell me about your plans for [RTS,S-eligible child] to receive additional doses of the malaria vaccine.<br><br>What are the reasons for your decision about taking the child for the next malaria vaccine dose?<br><br>When is the next malaria vaccine dose scheduled for [RTS,S-eligible child]?<br><br>How do you remember the date? Do you received anything to remind you?	4.6	Please tell me about if you have any plans to take [RTS,S-eligible child] to receive the malaria vaccine/additional doses of the malaria vaccine.<br><br>Please tell me about if you plan to have the RTS,S child catch-up on the missed malaria vaccine doses.<br><br>What are the reasons for your decision about taking the child for the next malaria vaccine doses?
4.7	Overall, how has your plan for the [RTS,S-eligible child] to receive the malaria vaccine changed or stayed the same since we last visited your household? Tell me why that is.		

Section 5-X	Other Topics (defined by the country) and Interview Closure
5.	Do you have any final thoughts about the malaria vaccine or other vaccines that you'd like to share with me now?
ADD ANY OTHER COUNTRY SPECIFIC ISSUES	

CLOSING SCRIPT:
Thank you for taking the time to discuss with me today. Your information you have provided will help us better understand the experiences that caregivers encounter as they seek the malaria vaccine for their children. This information will help inform ways to improve the delivery of the malaria vaccine.
