# Supplementary material for: Acceptance of and Adherence to a Four-Dose RTS,S/AS01 Schedule: Findings from a Longitudinal Qualitative Evaluation Study for the Malaria Vaccine Implementation Programme
Source: Vaccines (Basel). 2023 Dec 1;11(12):1801. doi: 10.3390/vaccines11121801 (PMC10747521; doi:10.3390/vaccines11121801)
Supplement: Supplementary file 1 [file vaccines-11-01801-s001.zip › Suppl 5_R3 Interview Guide.pdf]

NOTE: Please complete the Profile Sheet and Vaccination History Sheet before starting this interview.

Warm-up
Countries devise a warm-up question or two as they see fit. Please train interviewers to keep this section short. NOTE: Warm-up sections will not be needed for cross-country analyses if you don't wish to spend time and resources translating and transcribing this portion of the interview.

Section 1: RTS,S uptake

**I see from [RTS,S eligible child's] vaccination card that s/he received….**					
**…all four doses of the malaria vaccine.**		**…[1 to 3] doses of the malaria vaccine.**		**…no doses of the malaria vaccine**	
**ALL DOSES RCV'D (4)**		**PARTIAL DOSES RCV'D (1 – 3)**		**NO DOSES RCV'D (0)**	
I see from [RTS,S eligible child's] vaccination card that s/he received all four doses of the malaria vaccine.		I see from [RTS,S eligible child's] vaccination card that s/he received [1-3] doses of the malaria vaccine.		I see from [RTS,S eligible child's] vaccination card that s/he received no doses of the malaria vaccine.	
		1.1	How many doses of the malaria vaccine did you expect [RTS,S child] to receive?	1.1	Have you heard about the malaria vaccine?<br>• Yes → **continue to 1.2**<br>• No → **skip to 1.3**
1.1	When [RTSS-eligible child] received malaria vaccine doses, were you aware of it each time s/he received a dose, some of the times, or never?	1.2	When [RTSS-eligible child] received malaria vaccine doses, were you aware of it each time s/he received a dose, some of the times, or never?	1.2	Why has [RTS,S eligible child] not received any doses for the malaria vaccine?<br><br>*Probe to understand specific barriers.*
	If each and every time →<br>• In general, how did know that your child was due for another malaria vaccine dose?		***If each and every time →***<br>• In general, how did know that your child was due for another malaria vaccine dose?		***Re information barriers*** *(e.g., "I didn't know about the vaccine) ask*:<br>• How do you normally know that you are supposed to take your child for vaccination?
	If some of the times →<br>• When your child received the malaria vaccine without you knowing, did you become aware of this afterwards?<br>• How did you become aware of this?		***If some of the times →***<br>• When your child received the malaria vaccine without you knowing, did you become aware of this afterwards?<br>• How did you become aware of this?		***Re access barriers*** *(e.g., "I couldn't get to the clinic"), ask:*<br>• Is this a problem you've experienced with other vaccines? What do you usually do

I see from [RTS,S eligible child's] vaccination card that s/he received….					
**…all four doses of the malaria vaccine.**		**…[1 to 3] doses of the malaria vaccine.**		**…no doses of the malaria vaccine**	
	• Why do you think you weren't aware that you child was going to receive a malaria dose?<br>• How do you think this could it be avoided in the future?		• Why do you think you weren't aware that you child was going to receive a malaria dose?<br>• How do you think this could it be avoided in the future?		when this happens? Why couldn't you do the same in this instance?
	Never →<br>• When your child received the malaria vaccine without you knowing, did you become aware of this afterwards?<br>• How did you become aware of this?<br>• Why do you think you weren't aware that you child was going to receive a malaria dose?<br>• How do you think this could it be avoided in the future?		***Never →***<br>• When your child received the malaria vaccine without you knowing, did you become aware of this afterwards?<br>• How did you become aware of this?<br>• Why do you think you weren't aware that you child was going to receive a malaria dose?<br>How do you think this could it be avoided in the future?		***Re fears/rumors*** *involved, ask:*<br>• Where did you hear this from? / Who's saying this? Do you believe them? Why do you believe them?
1.2	Did you generally know in advance of going to the clinic that the child was due for a malaria vaccine?<br><br>How did you know?	1.3	Did you generally know in advance of going to the clinic that the child was due for a malaria vaccine?<br><br>How did you know?	1.3	If you could get [RTS,S-eligible child] vaccinated now for malaria, would you do that?<br><br>Why/why not?
1.3	Please tell me about the specific plans you made to take [RTS,S eligible child] for the malaria vaccination.	1.4	Tell me about the specific plans you made to take [RTS,S eligible child] for the malaria vaccination.		
1.4	Getting [RTS,S eligible child] all 4 doses of the malaria vaccine requires extra vaccination visits.<br><br>• Can you please tell me your specific reasons for taking [RTS,S eligible child] for all four vaccination visits?<br>• What helped you to make all four of these visits?<br>• What did the health workers do to making getting the vaccine for [RTS,S-eligible child] easier?	1.5	I notice that you missed some of your visits for the malaria vaccine.<br><br>• Can you tell me what happened that [RTS,S eligible child] has not yet received the [2nd/3rd/4th dose] of the malaria vaccine?<br>• What prevented you from making these visits?<br>*Probe to understand main barriers, such as access to the facility, time, awareness of schedule, concerns about the vaccine.*		

I see from [RTS,S eligible child's] vaccination card that s/he received….					
**…all four doses of the malaria vaccine.**		**…[1 to 3] doses of the malaria vaccine.**		**…no doses of the malaria vaccine**	
	Probe to understand any facilitators, such as reminder for when to return to the facility, called by a CHW, etc.				
1.6	Please describe your experiences at the clinic when [RTS,S eligible child received the malaria vaccine.<br><br>• What did you learn from health providers about the vaccine?<br>• Did you have any questions about the vaccine?<br>• What were they?<br>• Did you get your questions answered? Why/why not? What were you told?	1.6	Are you planning on taking the [RTS,S eligible child for another dose of the malaria vaccine?<br><br>Why/why not?		
1.7	Did the health workers tell you when [RTS,S-eligible child] received her/his last dose?<br><br>• What did they specifically tell you about this last dose?<br>• Anything else?	1.7	Please describe your experiences at the clinic when [RTS,S eligible child received the malaria vaccine.<br><br>• What did you learn from health providers about the vaccine?<br>• Did you have any questions about the vaccine?<br>• What were they?<br>• Did you get your questions answered? Why/why not? What were you told?		

Section 2: RTS,S Perceptions and Attitudes

**I now want to ask you about your perception of the malaria vaccine.**
2.1 Please tell me what you know about the malaria vaccine.<br><br>• Who is talking about the malaria vaccine within your community?<br>• What are the views of people who have taken their children for the malaria vaccine? Can you give me a specific example?<br>• What are the views of people who have not taken their child for the malaria vaccine? Can you give me a specific example?<br>• What have you heard about the malaria vaccine from others in your community?<br>• What else have you heard about the malaria vaccine from people in your community? *Probe to understand fully.*
2.2 I'm going to read several statements. After each statement, I will pause and I would like you to tell me what you think about the statement.<br><br>a. There is less malaria among children in the community ever since the malaria vaccine started to be given.<br>Do you agree or disagree with this statement?<br>Why do you agree with it? / Why don't you agree with the statement?<br>***Probe as needed:*** Can you elaborate? Can you describe what you mean? Can you give me an example?<br><br>b. Even though children in the community still get sick with malaria, the cases have been less severe than it used to be since the malaria vaccine started to be given.<br>Do you agree or disagree with this statement?<br>Why do you agree with it? / Why don't you agree with the statement?<br>***Probe as needed:*** Can you elaborate? Can you describe what you mean? Can you give me an example?<br><br>c. There is less malaria among children **in my household** ever since the malaria vaccine started to be given.<br>Do you agree or disagree with this statement?<br>Why do you agree with it? / Why don't you agree with the statement?<br>***Probe as needed:*** Can you elaborate? Can you describe what you mean? Can you give me an example?<br><br>d. Even though children **in my household** still get sick with malaria, it has been less severe than it used to be since the malaria vaccine started to be given.<br>Do you agree with this statement?<br>Why do you agree or disagree with it? / Why don't you agree with the statement?<br>***Probe as needed:*** Can you elaborate? Can you describe what you mean? Can you give me an example?
2.3 Has your understanding of the malaria vaccine changed in any way since we last visited?<br>• How so?<br>• What caused this change/these changes?

Section 3: Malaria Prevention

I now want to ask you about how you prevent malaria in your household.			
**ALL DOSES (4) or PARTIAL DOSES (1 – 3) RCV'D**		**NO DOSES RCV'D (0)**	
3.1	Did [RTS,S eligible child] sleep under a bed net last night?<br><br>Yes → Are there times s/he doesn't sleep under a net? Why does this occur?<br>No → Why doesn't s/he sleep under a net?	3.1	Did [RTS,S eligible child] sleep under a bed net last night?<br><br>Yes → Are there times s/he doesn't sleep under a net? Why does this occur?<br>No → Why doesn't s/he sleep under a net?
3.2	Has anything changes about how you use bed nets in your household since we last visited you?<br><br>What has changed? Why?	3.2	Has anything changes about how you use bed nets in your household since we last visited you?<br><br>What has changed? Why?

Section 4: Malaria Treatment

4. You told me earlier that [RTS,S-eligible child/other young child] has:			
**. . . suffered from malaria since our last visit to your household.**		**. . . not suffered from malaria since our last visit to your household.**	
4.1	I'd now like to ask you about this last time [RTS,S-eligible child] suffered from malaria. How did you first notice that your child had a problem?<br><br>*Probe to understand what health issues specifically alarmed the mother.*	4.1	I'm happy to hear your child has not had malaria recently. Given that malaria is very common in this community, how do you think your child/ren has/have avoided it recently?
4.2	At that time, why did you think the condition your child had was malaria?<br><br>*If mother initially thought it was not malaria, probe for what made mother begin to think it was a case of malaria. Probe to understand how mothers conceptualize malaria symptoms.*		

	Probe to understand the entire course of the disease from suspected diagnosis to confirmation, treatment, and resolution.		
4.3	Can you now please describe to me all the things that were done for your child while he/she was sick with malaria? Please start with the very first thing that was done for the child in the beginning and everything else that followed.<br><br>*Probe to get a complete picture of treatment action, from home remedies, drug shop cures, traditional healers, and resort to biomedical services.*		
4.4	Thinking about this episode of malaria, is there anything you could have done to prevent the child getting malaria? Can you please tell me more?		

Section 5: COVID-19 Perceptions and Attitudes

**I now want to ask you about how COVID-19 has affected your ability to receive vaccination services.**
5.1 Has COVID affected your community?<br><br>• How so?<br>• What about your own household?<br><br>5.2 How has COVID affected the way routine vaccinations are provided in your community (e.g. restriction in number of participants visiting clinics, no health talks etc.)?<br><br>5.3 During the COVID-19 outbreak, did you attend any vaccination clinics?<br><br>• Yes → What made you decide to take your child for a vaccination clinic?<br>• No → Why not? *[use probes in 5.2 as needed]*<br><br>5.4 Tell me about any difficulties you had in accessing the vaccination clinic during the COVID-19 outbreak.<br><br>*Probe for reasons that made it difficult to seek vaccination services, such as, facility closures, curfews, fear of COVID, lack of money or transport to reach the facility.*

Re facility closure or lack of staff (e.g. "The vaccination clinic was closed"), ask:

- How did you find out that the vaccination clinic was not available? What did you do when this happened? Did you go back to the clinic at another time?

Re access barriers (e.g., "I couldn't get to the clinic", "there were curfews", etc), ask:

- Tell me about what made it difficult to reach the clinic. What did you do when this happened? Did you go back to the clinic at another time?

  Probe:
  - What about travel restrictions or transit closures? Was this a problem for you? Why/how so?
  - What about curfews or stay at home orders? Was this a problem for you? Why/how so?
  - What about wearing a mask? Was this a problem for you? Why?/How so?
  - Did you have any concerns about being fined or detained? Please describe these for me.

Re fears of COVID (e.g. "I didn't want to get COVID"), ask:

- Tell me specifically about your concerns about getting COVID. What did you do about these concerns? Did the health workers do anything about your concerns?

5.5 Did the health services do anything that made it easier to attend the vaccination clinic during the COVID-19 outbreak?

Section 6: Other Topics (as defined by the country)

6.1 Do you have any final thoughts about the malaria vaccine or other vaccines that you'd like to share with me now?

Add any other country specific topics here
